# Supplementary material for: BCL11A Haploinsufficiency Causes an Intellectual Disability Syndrome and Dysregulates Transcription
Source: Am J Hum Genet. 2016 Jul 21;99(2):253–74. doi: 10.1016/j.ajhg.2016.05.030 (PMC4974071; doi:10.1016/j.ajhg.2016.05.030)
Supplement: Document S1. Figures S1–S11 and Tables S1 and S2 [file mmc1.pdf]

The American Journal of Human Genetics, Volume *99*

Supplemental Data

BCL11A Haploinsufficiency Causes an Intellectual Disability Syndrome and Dysregulates Transcription

Cristina Dias, Sara B. Estruch, Sarah A. Graham, Jeremy McRae, Stephen J. Sawiak, Jane A. Hurst, Shelagh K. Joss, Susan E. Holder, Jenny E.V. Morton, Claire Turner, Julien Thevenon, Kelly Mellul, Gabriela Sánchez-Andrade, Ximena Ibarra-Soria, Pelagia Deriziotis, Rui F. Santos, Song-Choon Lee, Laurence Faivre, Tjitske Kleefstra, Pentao Liu, Mathew E. Hurles, DDD Study, Simon E. Fisher, and Darren W. Logan

Supplemental Figures

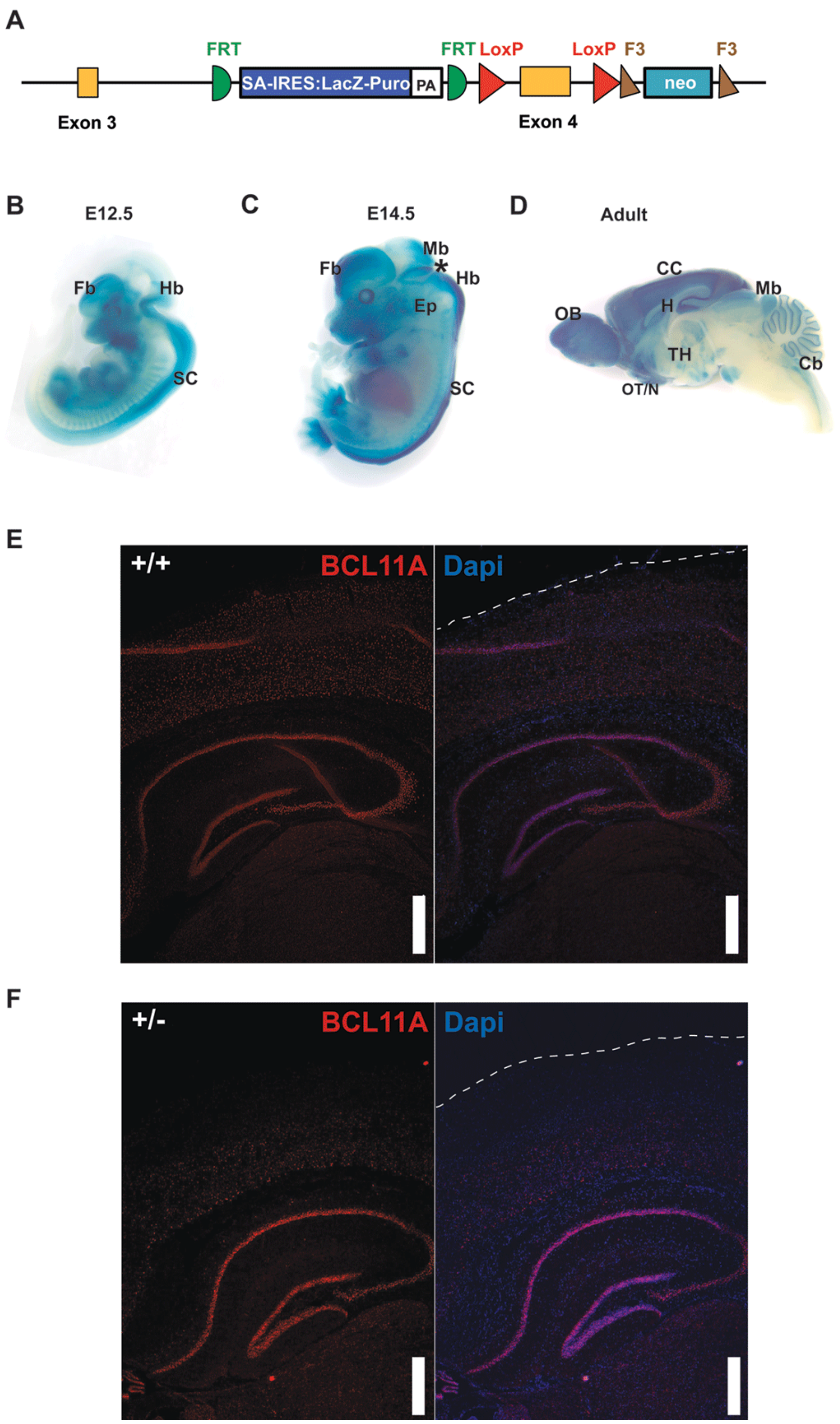

Figure S1. ***Bcl11a*** **expression in embryo and adult brain of** ***LacZ*** **knockout first reporter mouse.**

(A) Diagram of the knockout-first *LacZ* reporter allele with conditional potential. An *IRES:LacZ:Puro* promoter driven reporter cassette is inserted upstream of exon 4. **(B-D)** Whole mount images of X-gal staining patterns of $Bcl11a^{lacZ/+}$ mice at different developmental time

points. Expression of *Bcl11a* is first observed in the forebrain, derivatives of the first and second branchial arches and limb buds at E10.5 (not shown). **(B)** At E12.5 *Bcl11a* is high in the brain and neural tube. Specifically, it is highly expressed in the forebrain (Fb), hindbrain (Hb) and spinal cord (SC). *Bcl11a* is also seen in the somite derivatives, developing limbs, and maintains expression in the derivatives of the first two branchial arches. **(C)** At E14.5 *Bcl11a* maintains high expression in the brain, namely forebrain (Fb), midbrain (Mb) and hindbrain (Hb), with marked expression in the mid to hindbrain junction (*). Interestingly, high expression is seen in the ear pinnae (Ep). **(D)** In the adult brain, high expression of *Bcl11a* is detected in the cerebral cortex (CC), hippocampus (H), olfactory bulb (OB), olfactory tubercule and nucleus accumbens (OT/N). Parts of the thalamus (T) and a restricted area on the ventral aspect of the pons also show expression. The cerebellum (Cb), especially the purkinje cell layer, also show expression of *Bcl11a*.

E,F) Immunohistochemistry of wildtype ($Bcl11a^{+/GFP}$) and heterozygous mutant ($Bcl11a^{LacZ/GFP}$) GFP-reporter mice respectively. BCL11A localization is determined by immunolabelling using an anti-BCL11A antibody (red). Localization is identified throughout the cortical layers with exception of outer layer I, the hippocampus (specifically the pyramidal cell layer and dentate gyrus), and less intensely in the thalamus. Nuclei are counterstained with DAPI (blue). Bars: 500 µm.

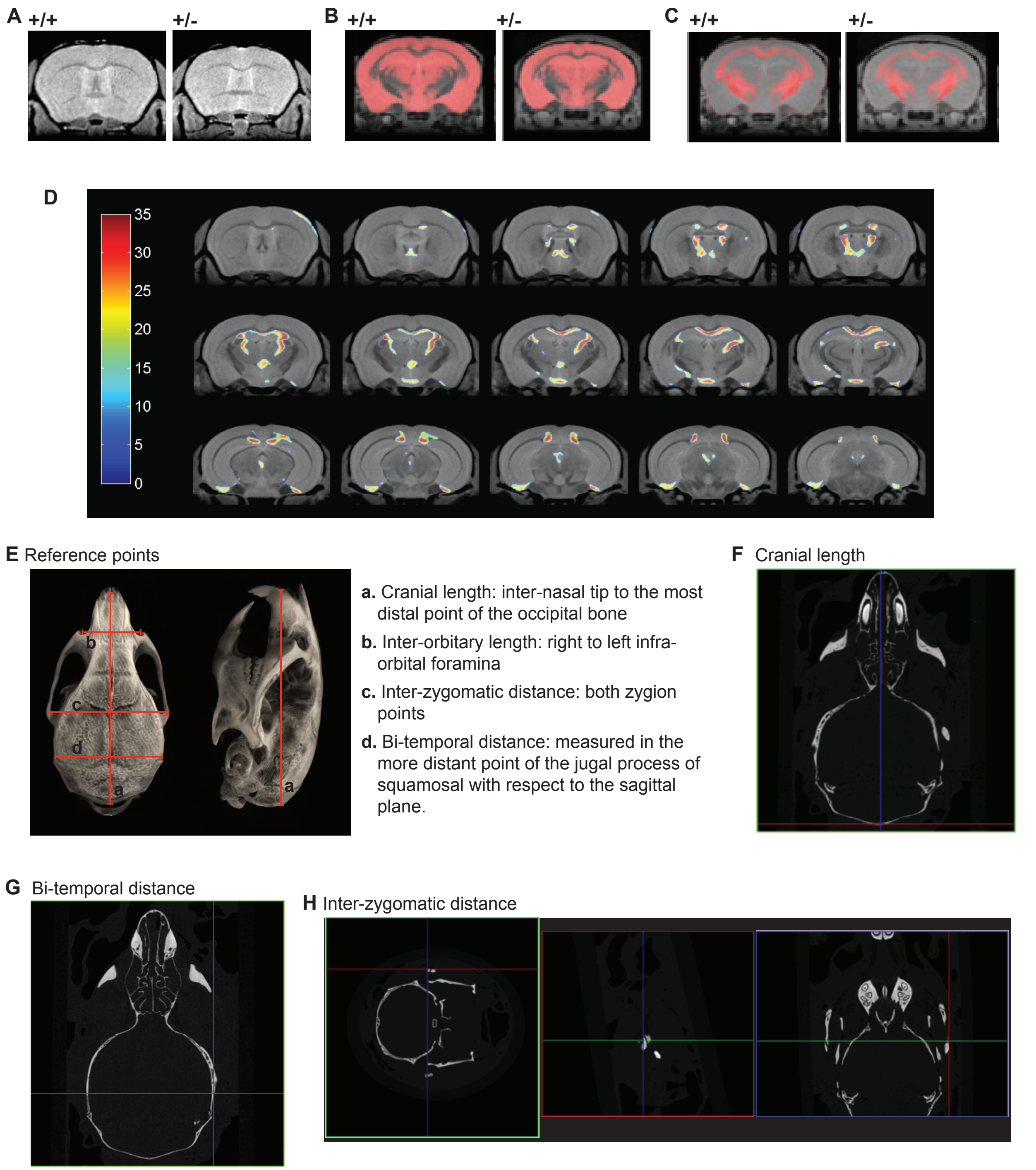

Figure S2. Neuroimaging shows microcephaly in *Bcl11a*$^{+/-}$ mice.

(A) MRI coronal sections of wildtype (**+/+**) and *Bcl11a*$^{+/-}$ **(+/-)** mice show decreased overall brain size in mutants. Representative images per genotype showing significant reduction in grey matter **(B)** and white matter **(C)** volume. **(D)** Significant changes in *Bcl11a*$^{+/-}$ mice after normalization of overall brain size are shown on representative pseudocoloured coronal

sections. Color bar indicates two-tailed F-test corrected for multiple comparisons by controlling the false-discovery rate at $q<0.05$. All changes shown are significant at $q<0.05$.

(E) to **(H)** represent 3D cranial µCT measurements methods. **E)** Landmarks used for measurements. **(F)** Cranial length, measured between the internasal (tip of the nose) and the occipital (the most distal point of the occipital bone) points. **(G)** Bi-temporal distance, measured in the most distant point of the jugal process of the squamosal bone with respect to the sagittal plane. **(H)** Inter-zygomatic distance, measured between both zygion points. As demonstrated in **(H)**, all measurements were corrected using a multiplanar visualization of reference points, in sagittal, axial and coronal planes.

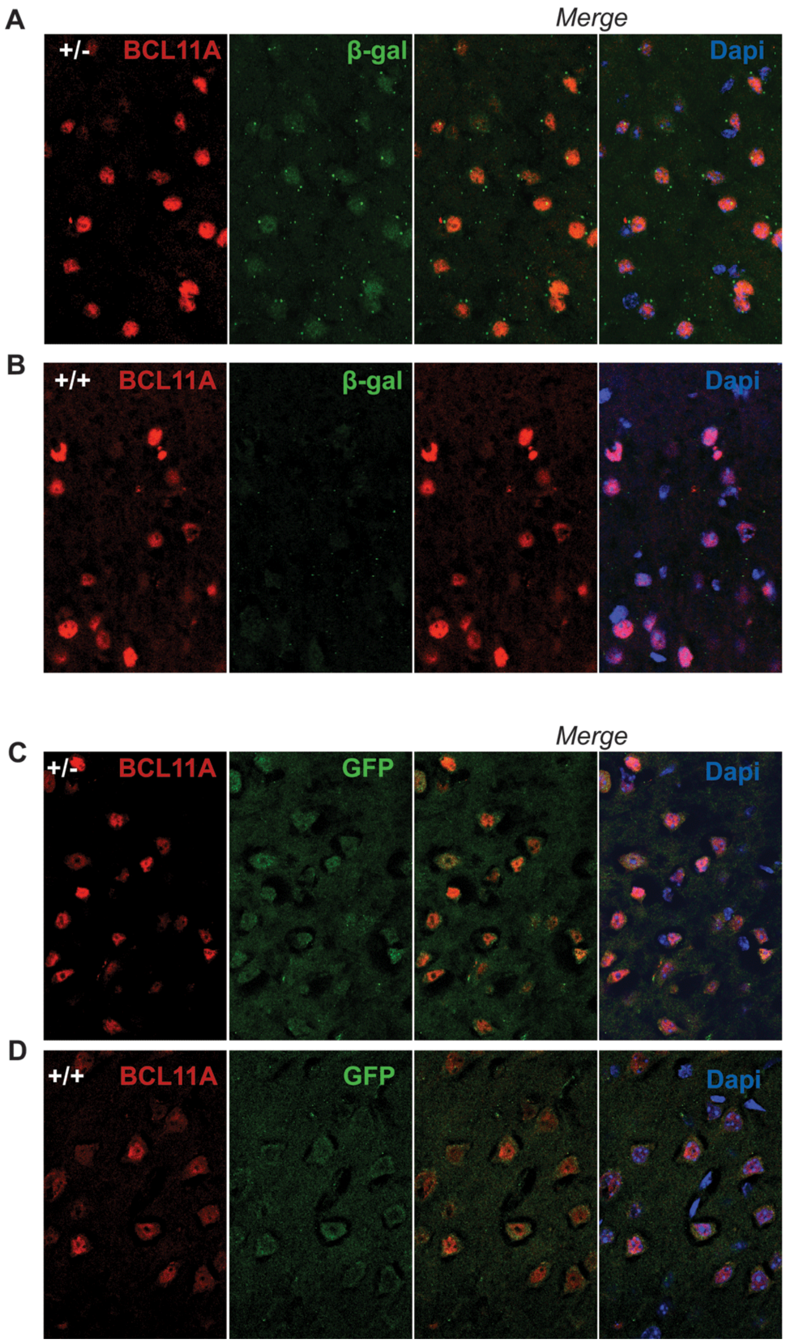

Figure S3. BCL11A antibody immunostaining.

Immunostaining of cortical cryosections in mutant (*Bcl11a*$^{LacZ/GFP}$, represented by +/-) and wildtype (*Bcl11a*$^{+/GFP}$, represented by +/+) GFP reporter mice (with a primary antibody predicted to detect the L and XL isoforms) shows nuclear staining. **(A)** An anti-BCL11A Ab (red) co-localizes with anti-β-GAL (β-GAL, green) in the mutant (heterozygote for the knockout-first *LacZ* reporter allele), which is absent in the wildtype **(B)**. Anti-GFP Ab (green) was employed to detect the functional GFP reporter allele **(C,D)**, which co-localizes with anti-BCL11A (red) in nuclei. Nuclei are counterstained with DAPI (blue).

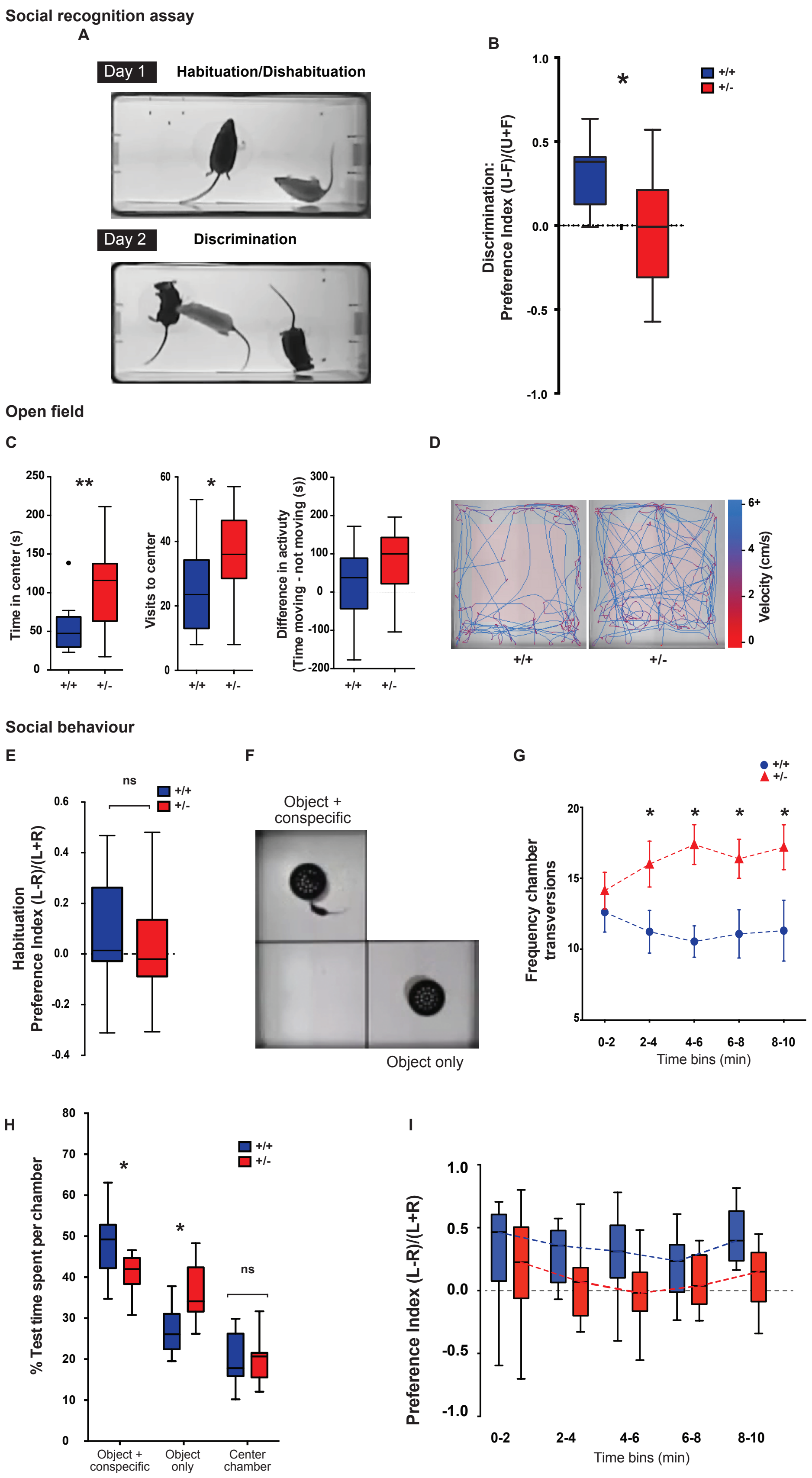

Figure S4. Cognitive and behavioral phenotyping of *Bcl11a*$^{+/-}$ mice.

(A) Overhead image of a representative test arena for days 1 (top), and 2 (bottom) social recognition assay. In the example presented, the test *Bcl11a*$^{+/-}$ mice are a light coat color (albino), and the stimuli are dark. On both days prior to testing mice are habituated for ≥1 hour in the behavioral test room and ≥ 10 minutes to the empty test cage; habituation and test are performed under red light. On day 1, test animals are presented with an anaesthetized conspecific for 1 minute, at 10-minute intervals, for 4 trials. On the 5th trial, a novel stimulus is presented. Investigation time, identified as close proximity sniffing, oronasal contact, or approaching within 1-2cm, is recorded over 2 minutes. **(B)** Social discrimination preference index (PI) values greater than 0.5 (dotted line) indicate that the test mice spent greater amount of time investigating the unfamiliar stimulus compared with the familiar stimulus. *Bcl11a*$^{+/+}$, *n*=11; *Bcl11a*$^{+/-}$, *n*=12. *Bcl11a*$^{+/-}$ mice have a significantly decreased preference for the unfamiliar stimulus compared to the wildtype (t-test, **p*=0.012). Boxes indicate 25th, median and 75th percentiles; whiskers indicate minimum and maximum values.

(C,D) Open field activity. *Bcl11a*$^{+/-}$ mice spent more time in the center (Mann-Whitney, ***p*=0.003) than wildtype littermates. The number of visits to the center was also increased (t-test, **p*=0.025). Increased difference in activity (defined as the difference between the time moving and not moving) was just below statistical significance (t-test, *p*=0.091), as was velocity (t-test, *p*=0.0892). Tukey boxplots; black dots indicate outliers (*Bcl11a*$^{+/+}$, *n*=14; *Bcl11a*$^{+/-}$, *n*=17). **(D)** Examples of trajectories (representative tracks closest to the mean) of a wildtype (+/+) and *Bcl11a*$^{+/-}$ mouse.

(E-G) Three chamber social behavior test. Mice are habituated for ≥1 hour in the behavioral test room; habituation and test are performed under red light. Test mice are habituated to the center chamber for 5 minutes, and to the three chambers (by opening doors to left and right chambers) for another 5 minutes. During habituation, movements are tracked with an automated recording system. The preference index for left vs. right chamber is calculated by the following equation: [PI=(time in left chamber – time in right chamber)/ (time in left chamber + time in right chamber)]. No significant difference between genotype was detected (t-test, *p*=0.414). Boxes indicate 25th, median and 75th percentiles; whiskers indicate minimum and maximum values. **(F)** A cylindrical metal container with holes that fit a mouse nose tip is placed in the two side chambers: one containing a live conspecific stimulus, the other empty. An automated recording system is used to track the movements of the test mouse and record time spent in each chamber during a 10 minute test. **(G)** Number of transversions from one chamber to another over the test time in 2 minute time bins is significantly increased after the first 2 minutes in *Bcl11a*$^{+/-}$ mice (t-test per time bin, *p*<0.05 for minutes 2 to 10; 2-way ANOVA *p*=0.0068 for genotype). Values are mean number of transversions ± S.E.M.

(H) The boxplot represents the percentage of time spent in each chamber (boxes indicate 25th, median and 75th percentiles; whiskers indicate minimum and maximum values). There is a significant difference between genotypes for time spent in the object + conspecific chamber (**p*=0.003, t-test) and for time spent in object only chamber (**p*=0.0007), but not for the empty center chamber (*p*=0.826). **(I)** Boxplots of preference index across 2 minute time bins (boxes indicate 25th, median and 75th percentiles; whiskers indicate minimum and maximum values; *Bcl11a*$^{+/+}$, *n*=12; *Bcl11a*$^{+/-}$, *n*=16. Black dots indicate outliers).

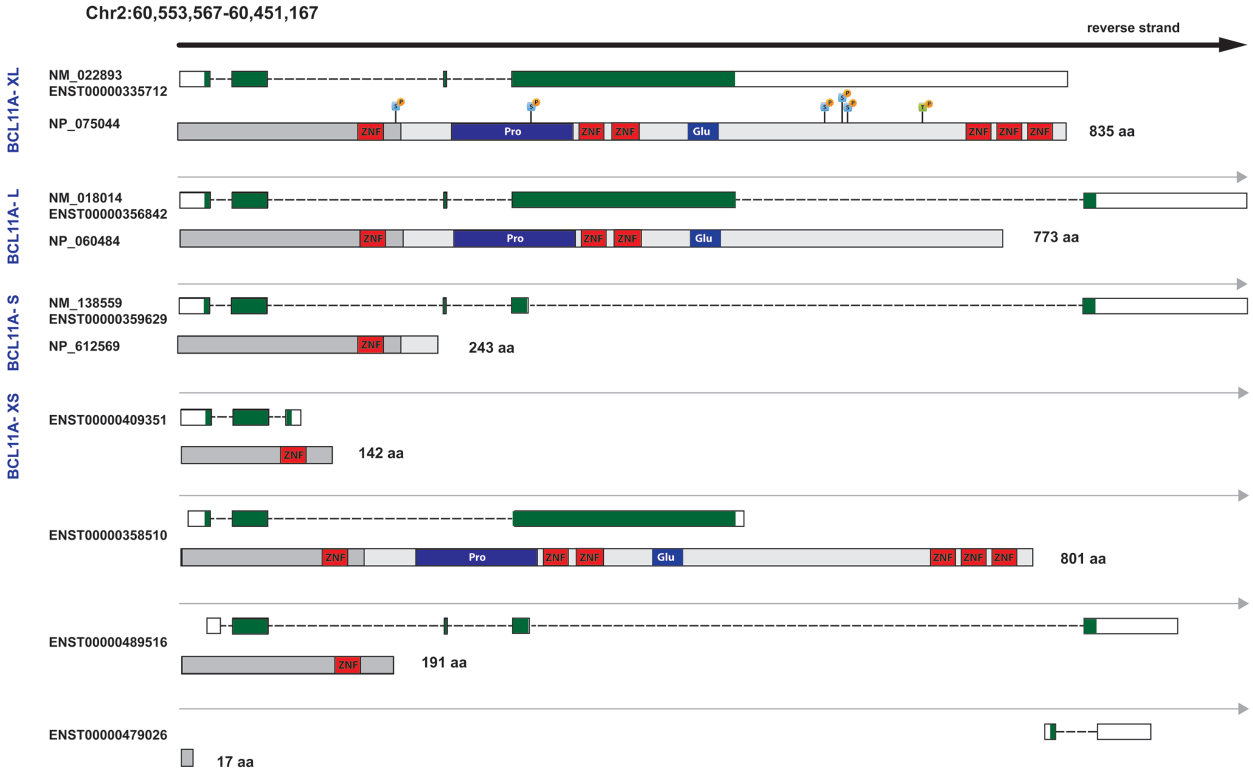

Figure S5. BCL11A isoforms. Isoforms putatively encoded by *BCL11A* and annotated in RefSeq and Ensembl. Accession numbers are indicated in black, and "common" name for the 4 isoforms reported are in blue. A schematic diagram of alternative exon usage is provided above each predicted protein. Top: Exons are shaded dark green; UTRs are white. Bottom: C2H2 zinc finger domains are represented in red. Putative region required for SUMO1 recruitment is shaded grey. Proline (Pro) and Glutamate (Glu) rich region are shaded blue. aa, aminoacids. On the XL isoform, post-translational modifications annotated in UniProt are indicated: phosphorylated serine in blue, phosphorylated threonine in green.

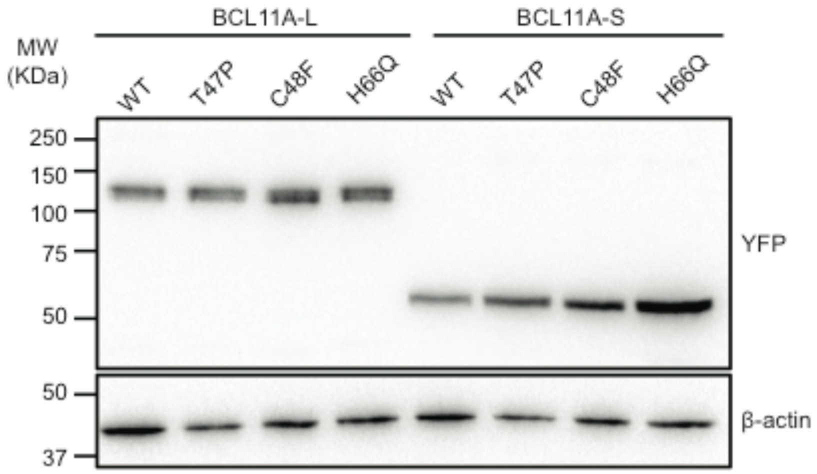

Figure S6. Western blot of mutant and wildtype BCL11A-L and BCL11A-S. HEK293 cells were transfected with mutant or wildtype (WT) *BCL11A* fused to YFP. Blots of whole cell lysates were probed with anti-YFP to detect BCL11A and with anti-β-ACTIN to confirm equal loading. BCL11A-L: NM_018014.3, ENST00000356842; BCL11A-S: NM_138559, ENST00000359629.

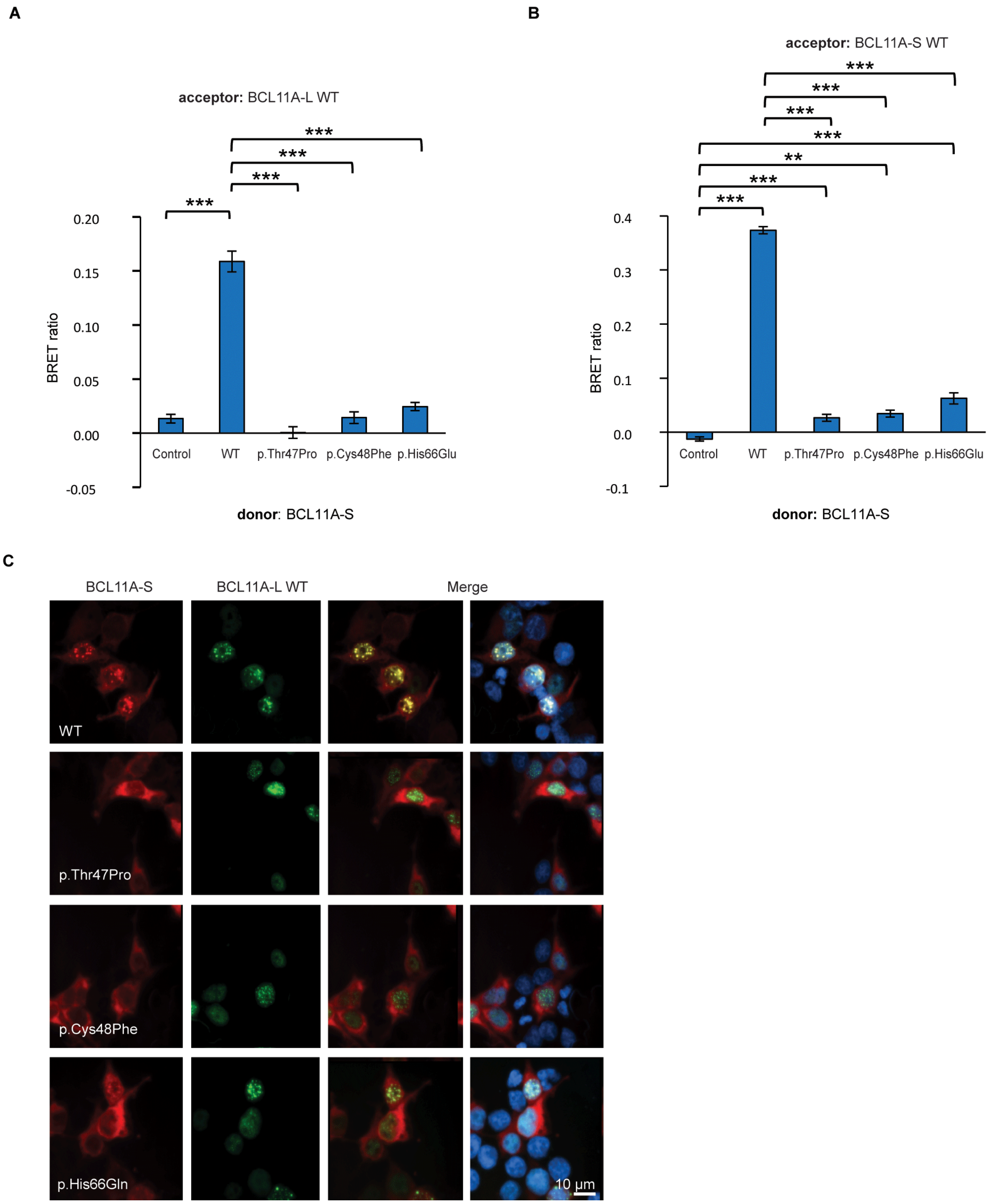

Figure S7. *BCL11A* missense mutations identified in affected individuals disrupt protein dimerization. (A) BRET assay for interaction of mutant BCL11A-S with wildtype (WT) BCL11A-L. HEK293 cells were transfected with wildtype or mutant BCL11A-S fused to *Renilla* luciferase (donor) and wildtype BCL11A-L fused to YFP (acceptor). Values are mean corrected BRET ratios ± S.E.M. (*n*= 3, **p*<0.05, ***p*<0.01, ****p*<0.001, *one-way ANOVA* followed by *Bonferroni* post-hoc correction). **(B)** BRET assay for interaction of mutant BCL11A-S with wildtype BCL11A-S. HEK293 cells were transfected with wildtype or mutant BCL11A-S fused to *Renilla*

luciferase (donor) and wildtype BCL11A-S fused to YFP (acceptor). Values are mean corrected BRET ratios ± S.E.M. (*n*= 3, *$p<0.05$, **$p<0.01$, ***$p<0.001$, *one-way ANOVA* followed by *Bonferroni* post-hoc correction). **(C)** Fluorescence micrographs of HEK293 cells transfected with wildtype or mutant BCL11A-S fused to mCherry and wildtype BCL11A-L fused to YFP. Nuclei were stained with Hoechst 33342 (blue). BCL11A-S: NP_612569.1; BCL11A-L: NP_060484.2.

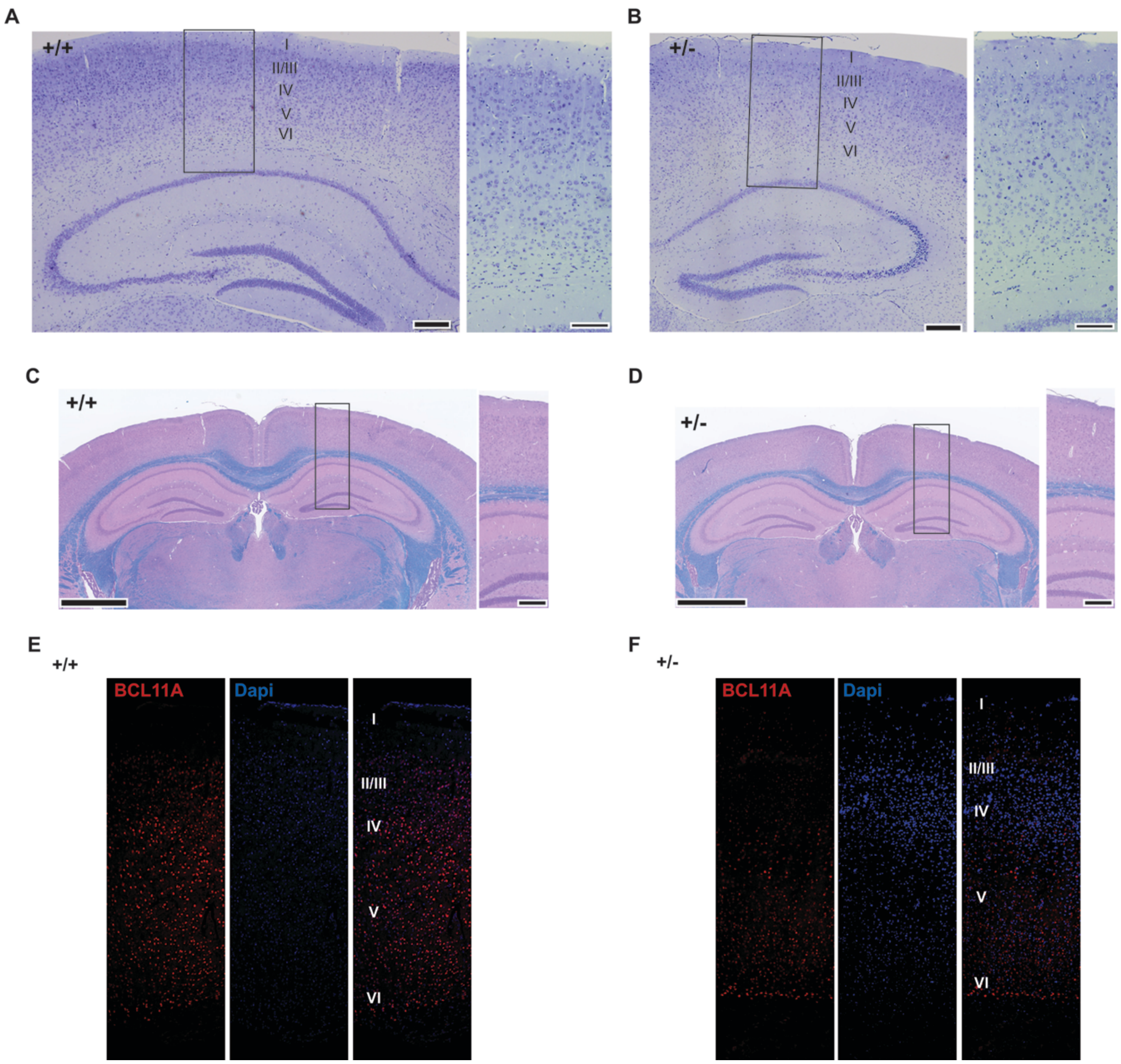

Figure S8. Cortical morphology in wildtype and *Bcl11a*$^{+/-}$ mice. *Bcl11a* haploinsufficient mice (+/-, **B,D**) do not present gross morphological defects in comparison to wildtype littermates (+/+, **A,C**). Nissl and luxol fast blue staining of paraffin embedded sections of brains of *Bcl11a*$^{+/-}$ mice **(B,D)** displayed brain architecture similar to wildtype littermates **(A,C)** at 16 weeks, specifically with normal structure of the neocortex and hippocampus. Higher magnifications are delimited by inset boxes. Bars: A,C: 200 μm (inset amplification, 100 μm); B,D: 1mm (inset amplification, 200 μm).

E,F: Immunostaining of the cortex for BCL11A (first panels, red) in mutants (+/-, **F**) mice and wildtype littermates (+/+, **E**) and DAPI nuclear counter-stain (second panels, blue); the third panels are the merged images. BCL11A localization is detected in layers II to VI in both genotypes. Unlike wildtype, *Bcl11a*$^{+/-}$ mice show more BCL11A stained nuclei in deep layers in comparison to upper layers.

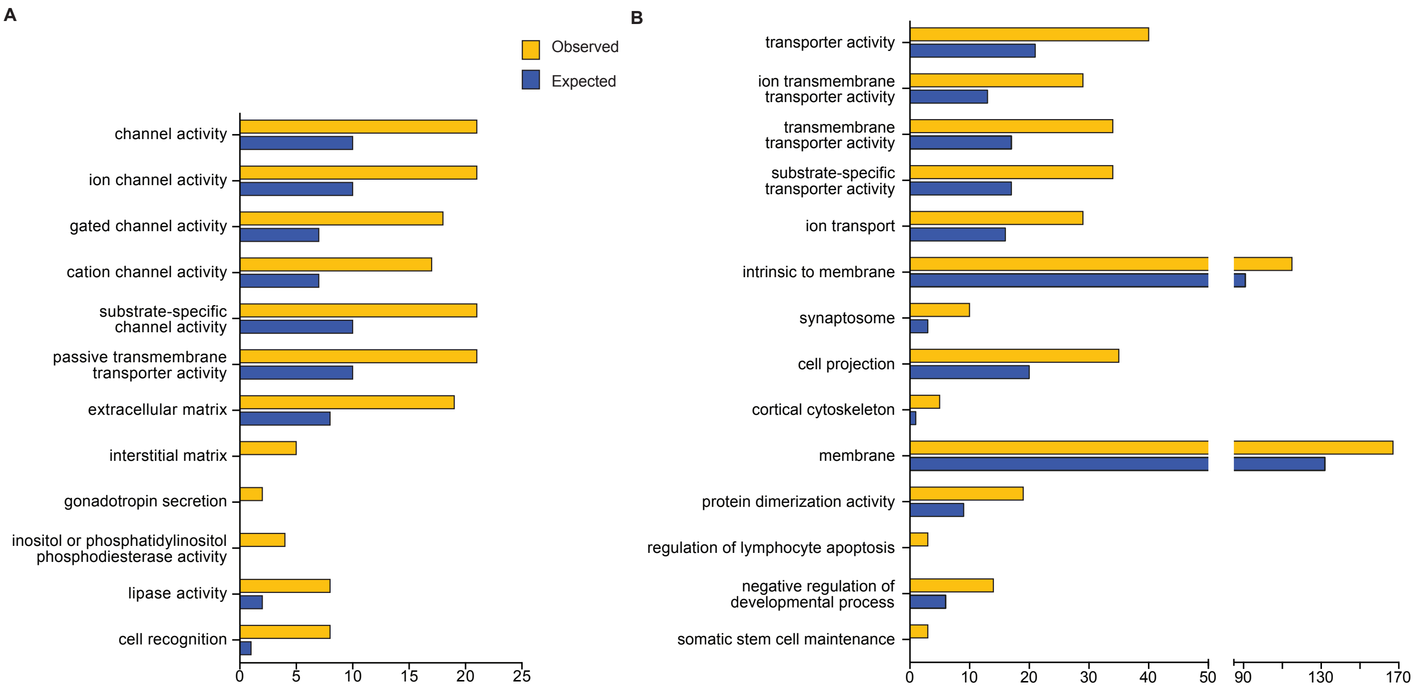

Figure S9. Gene ontology enrichment analysis of differentially expressed genes in *Bcl11a*$^{+/-}$ mice.

Selected categories of the GeneTrail over-representation analysis of differentially expressed genes in the cortex **(A)** and hippocampus **(B)** compared to a reference set of genes expressed in each wildtype tissue. The blue bar indicates the expected number of genes; the yellow bars indicate the observed number of genes for gene ontology terms. Significance threshold p<0.1 Hypergeometric test with FDR adjustment (Benjamini Hochberg, 1995) for multiple testing (complete analysis results and individual *p-value*s available in Supplemental Table 2).

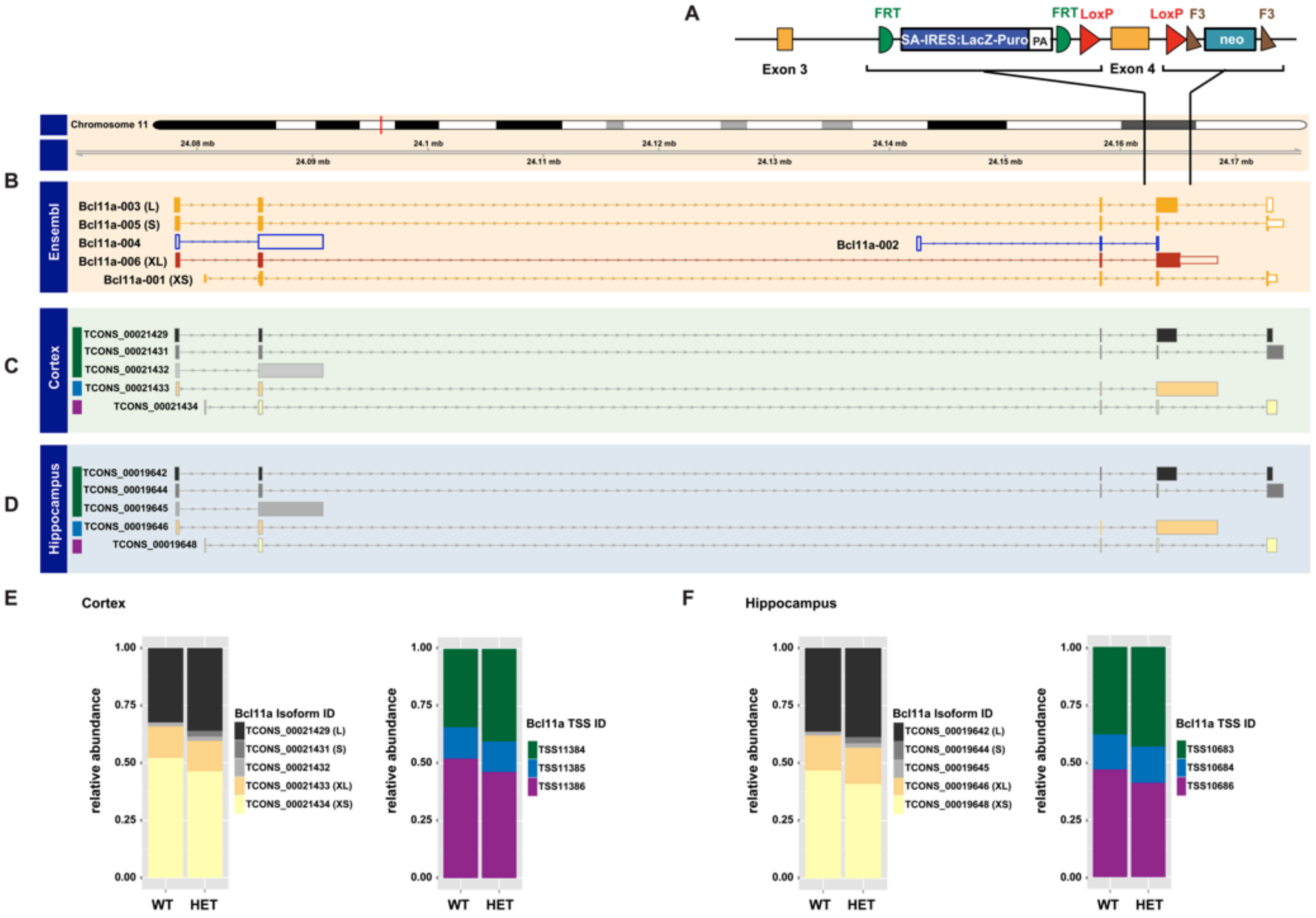

Figure S10. Bcl11a *LacZ* reporter allele and isoform expression.

(A) Diagram of the knockout-first *LacZ* reporter allele with conditional potential described in Figure S3. The cassette disrupts transcription of all reported protein-coding isoforms as represented in **(B)** (chromosome 2 ideogram and Ensembl release 82 isoforms shown; graphical representation of isoforms created with CummeRbund v2.12.0). Isoforms represented in yellow and red are protein coding; blue isoforms do not produce protein. Bcl11a-001: isoform XS, NM_001159290, ENSMUST00000118955; Bcl11a-002: processed transcript ENSMUST00000127494; Bcl11a-003: isoform L, NM_016707, ENSMUST00000000881; Bcl11a-004: retained intron ENSMUST00000124148; Bcl11a-005: isoform S, NM_001159289, ENSMUST00000109516; Bcl11a-006: isoform XL, NM_001242934, ENSMUST00000109514. Isoforms assembled in Cufflinks v.2.2.1 with fpkm≥0.1 in cortex and hippocampus (both genotypes pooled per tissue) are represented in **(C)** and **(D)** respectively. Differential expression (without correction for batch effect) of each isoform per genotype and respective transcription start site usage are represented in **(E)** and **(F)**. The four major mouse isoforms (L, S, XL and XS) are expressed in both tissues in similar proportions. There is a relative decrease of isoform XS in favor of L and S in mutants (HET) when compared to wildtype (WT).

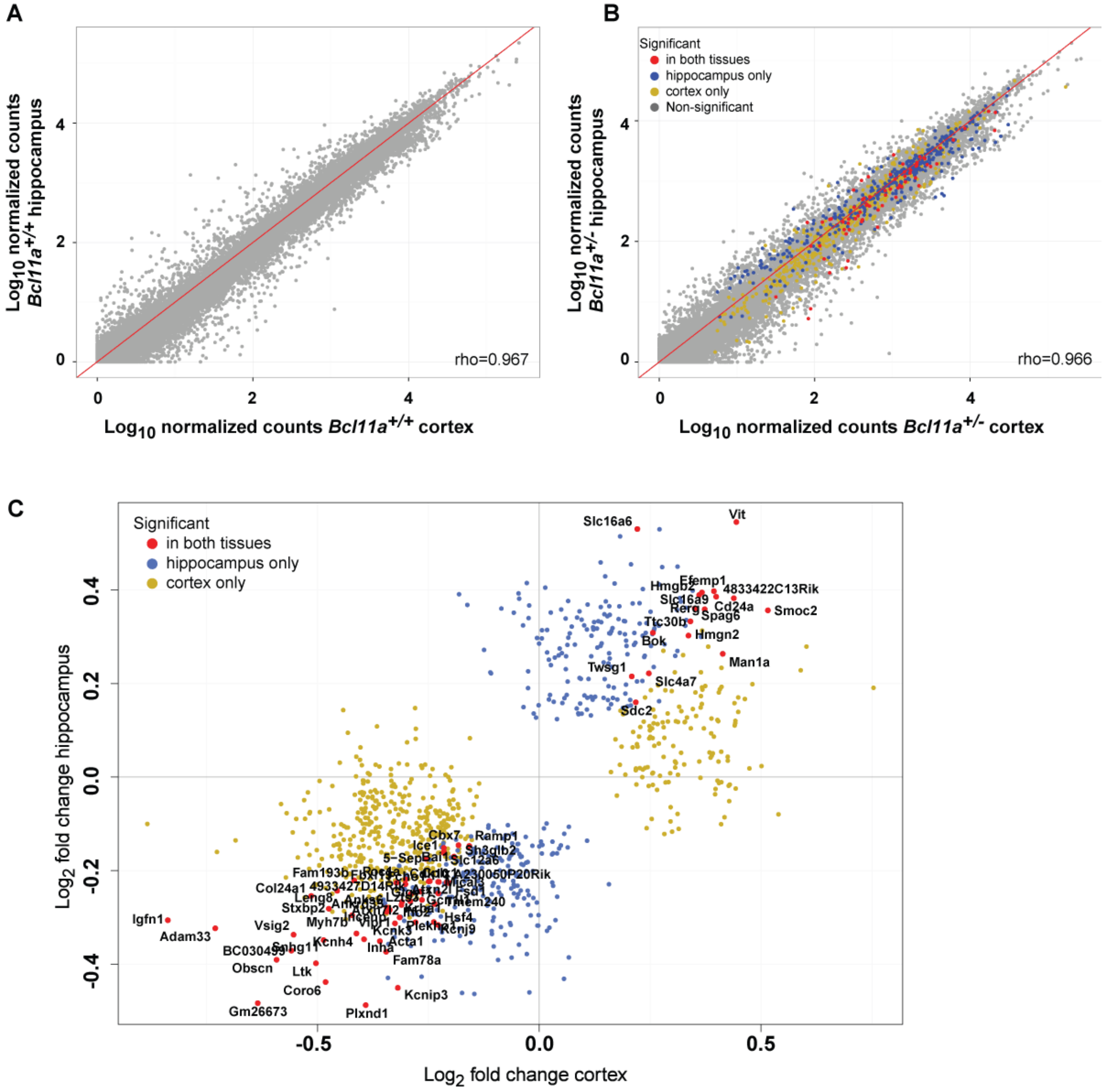

Figure S11. Comparison of gene expression per tissue.

A) Distribution of $\log_{10}$+1 normalized counts in wildtype cortex vs. hippocampus. Spearman's correlation value in bottom right corner ($p<2.2e^{-16}$). **B)** Distribution of $\log_{10}$+1 normalized counts in *Bcl11a*$^{+/-}$ cortex vs. hippocampus. Spearman's correlation value in bottom right corner ($p<2.2e^{-16}$). Differentially expressed genes are represented in color. **C)** Comparison of DESeq2's shrinkage estimation of log fold changes of genes in both tissues as in Figure 6. Red dots represent genes with significant differential expression in both tissues, labeled by gene name. Yellow dots represent genes differentially expressed in the cortex only, and blue in the hippocampus only (BH-adjusted *p*-value <0.1). Non-differentially expressed genes are not represented.

Gene	Forward primer	Site	Reverse primer	Site
BCL11A-S	GGATCCTGTCTCGCCGCAAGCAAGGC	BamHI	GCTAGCTCAAATTTTCTCAGAACTTAAGGGC	NheI
BCL11A-L	GGATCCTGTCTCGCCGCAAGCAAGGC	BamHI	GCTAGCTCAGAACTTAAGGGCTCTCG	NheI
NONO	GGATCCAGAGTAATAAAACTTTTAACTTGG	BamHI	TCTAGATTAGTATCGGCGACGTTTGTTTGGG	XbaI

Table S1. Primers used for human fetal brain cDNA amplification. Restriction sites are underlined. NheI and XbaI have compatible overhangs.

Variant	Primer 1	Primer 2
BCL11A T47P	GCACTGCCCACAGGGGAGGAGGTCATGAT	ATCATGACCTCCTCCCCTGTGGGCAGTGC
BCL11A C48F	GGCACTGCCCAAAGGTGAGGAGGTCATGATCC	GGATCATGACCTCCTCACCTTTGGGCAGTGCC
BCL11A H66Q	CTGCCATTGCATTGTTTCCGTTTTTGCTCGATAAAAATAA GAATGTC	GACATTCTTATTTTTATCGAGCAAAAACGGAAACAATGCAAT GGCAG

Table S2. Primers used for introduction of missense mutations identified in affected individuals in DNA constructs.

Table S3. RNA-Seq of *Bcl11a*$^{+/-}$ *and Bcl11a*$^{+/+}$ mouse cortex and hippocampus.

Provided as a separate .xlsx file

Excel file with sheets containing RNASeq analysis of cortex and hippocampus: RNASeq normalized read counts, results tables from DESeq2 differential expression analysis, gene ontology enrichment analysis and *Bcl11a* isoform predictions determined with Cufflinks.

Table S4. Gene lists used in specific gene enrichment analysis.

Provided as a separate xlsx file

Excel file with sheets containing gene subset for each enrichment analysis.
