# Supplementary figures and images for: BCL11A Haploinsufficiency Causes an Intellectual Disability Syndrome and Dysregulates Transcription
Source: Am J Hum Genet. 2016 Jul 21;99(2):253–74. doi: 10.1016/j.ajhg.2016.05.030 (PMC4974071; doi:10.1016/j.ajhg.2016.05.030)

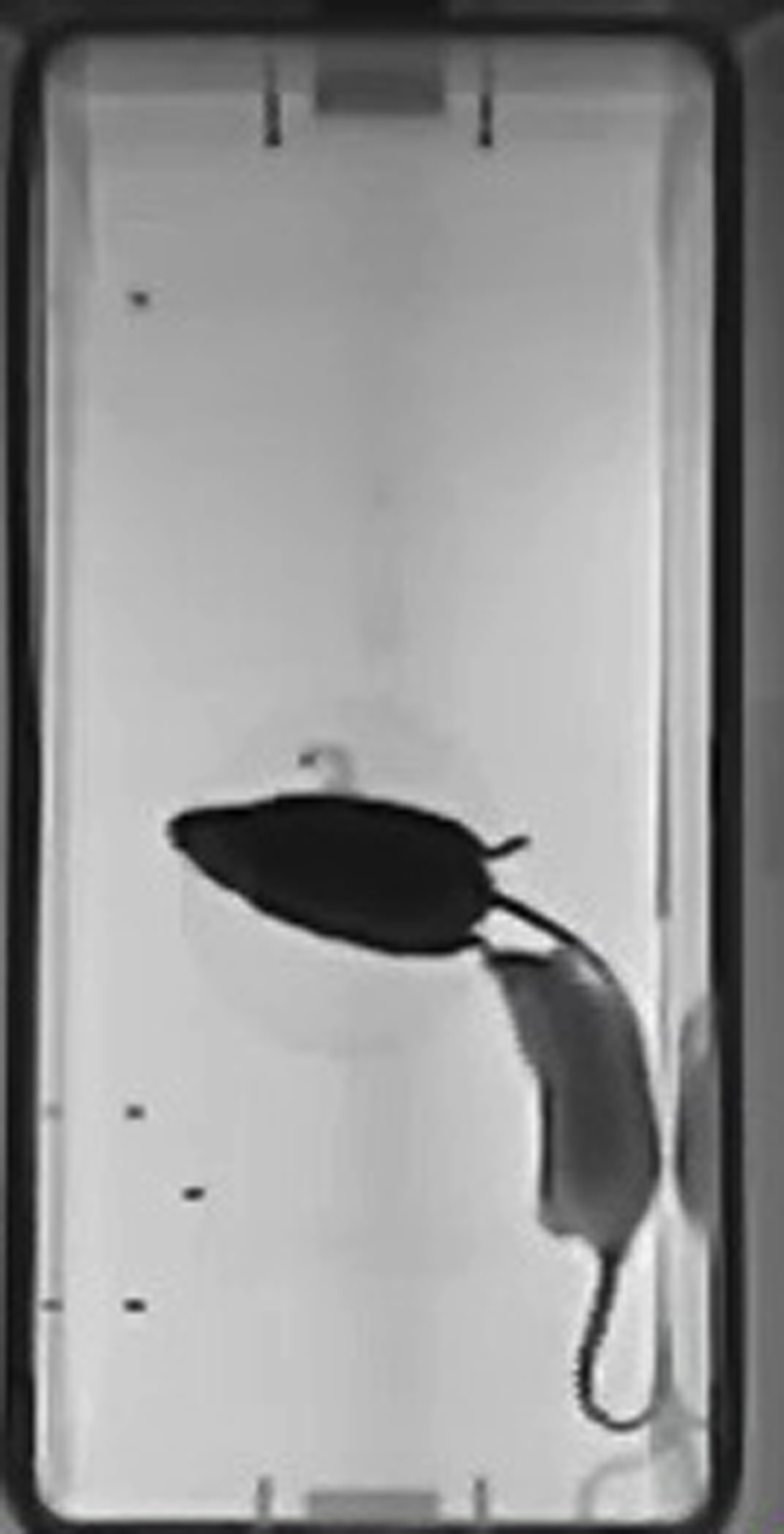

Supplement: Movie S1. Habituation-Dishabituation on Day 1 of Social Recognition Assay — The video shows a representative trial of the habituation-dishabituation test on day 1 of the social recognition assay captured by an overhead infer-red video camera. An anaesthetized stimulus mouse was placed in the center of the test arena; the amount of time the test animal spent investigating by close proximity sniffing, oronasal contact, or approaching within 1-2 cm, was recorded. [file mmc4.jpg]

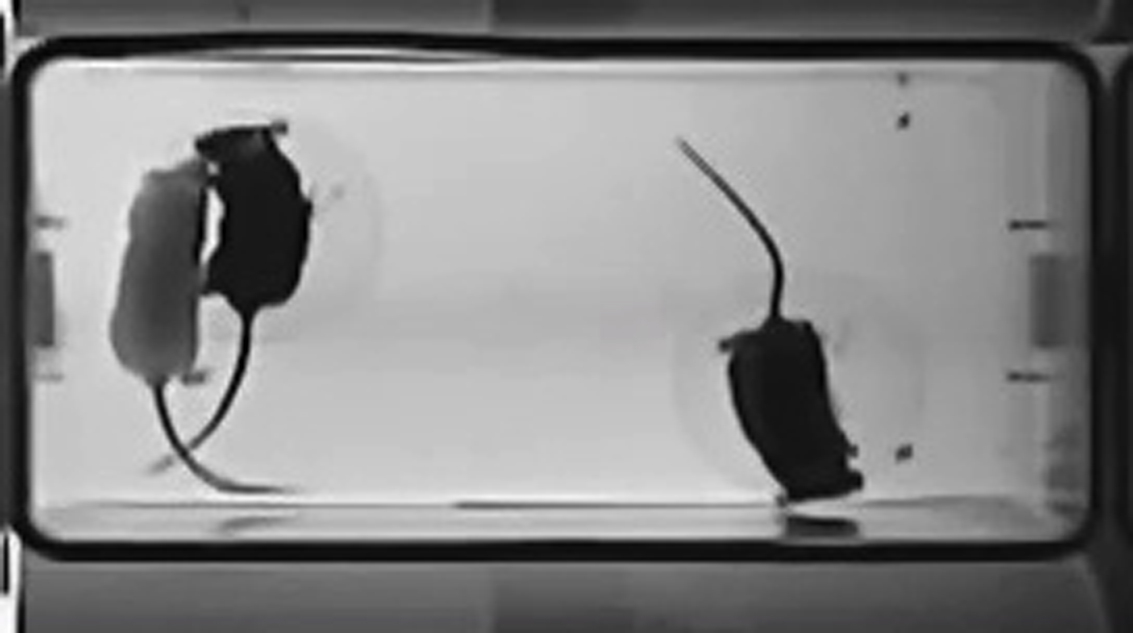

Supplement: Movie S2. Discrimination Test on Day 2 of Social Recognition Assay — The video shows a representative trial of the discrimination test on day 2 of the social recognition assay captured by an overhead infra-red video camera. A familiar stimulus animal from trials 1-4 and a new unfamiliar mouse were placed on opposite sides of the test arena; the time the test animal spent investigating each stimulus mouse was recorded. [file mmc5.jpg]

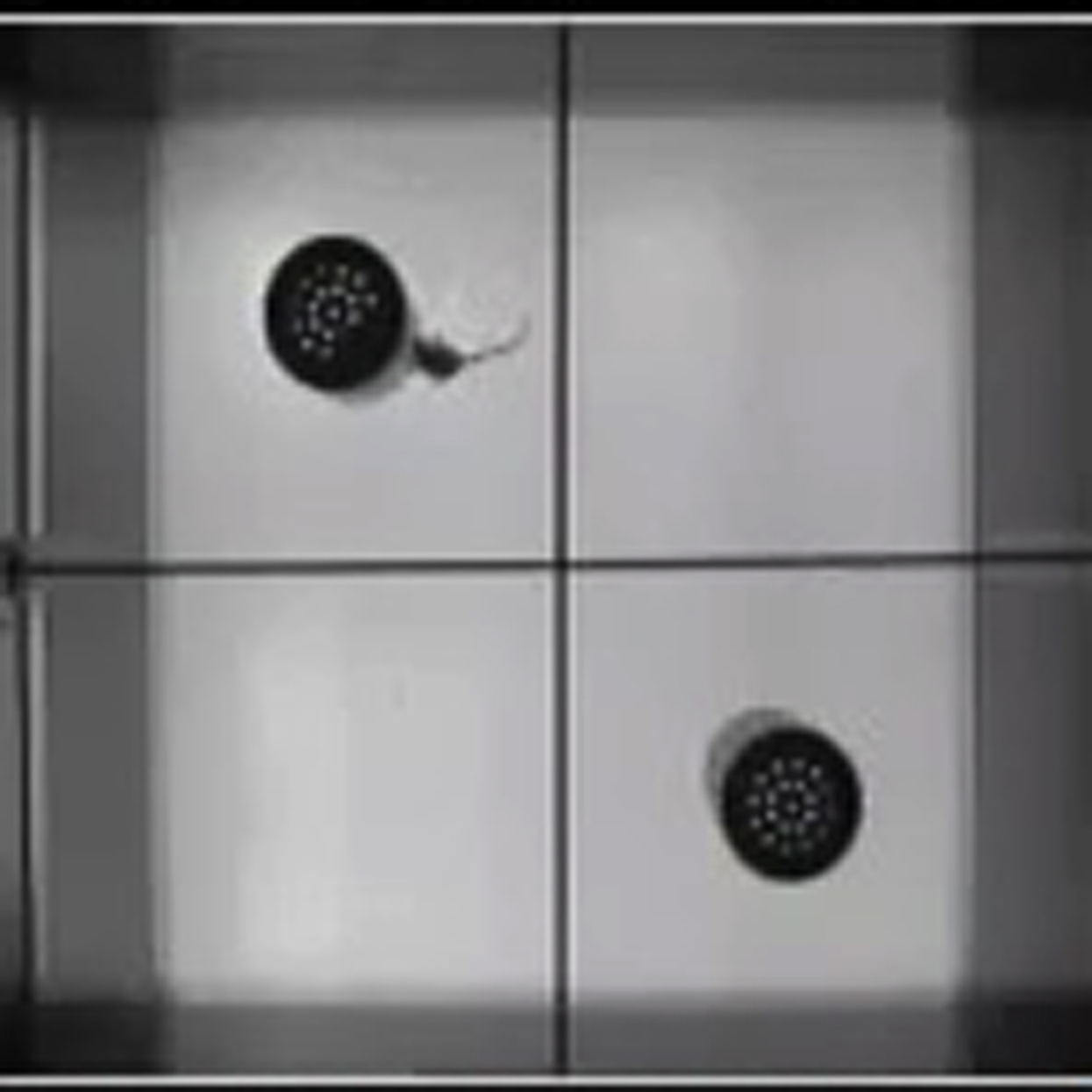

Supplement: Movie S3. Three-Chamber Social Behavior Assay Test — The video shows a segment of a representative trial of the three chamber social behavior assay, recorded by an overhead infra-red video camera. Automated tracking is used to detect the test mouse and record time spent in each chamber. [file mmc6.jpg]

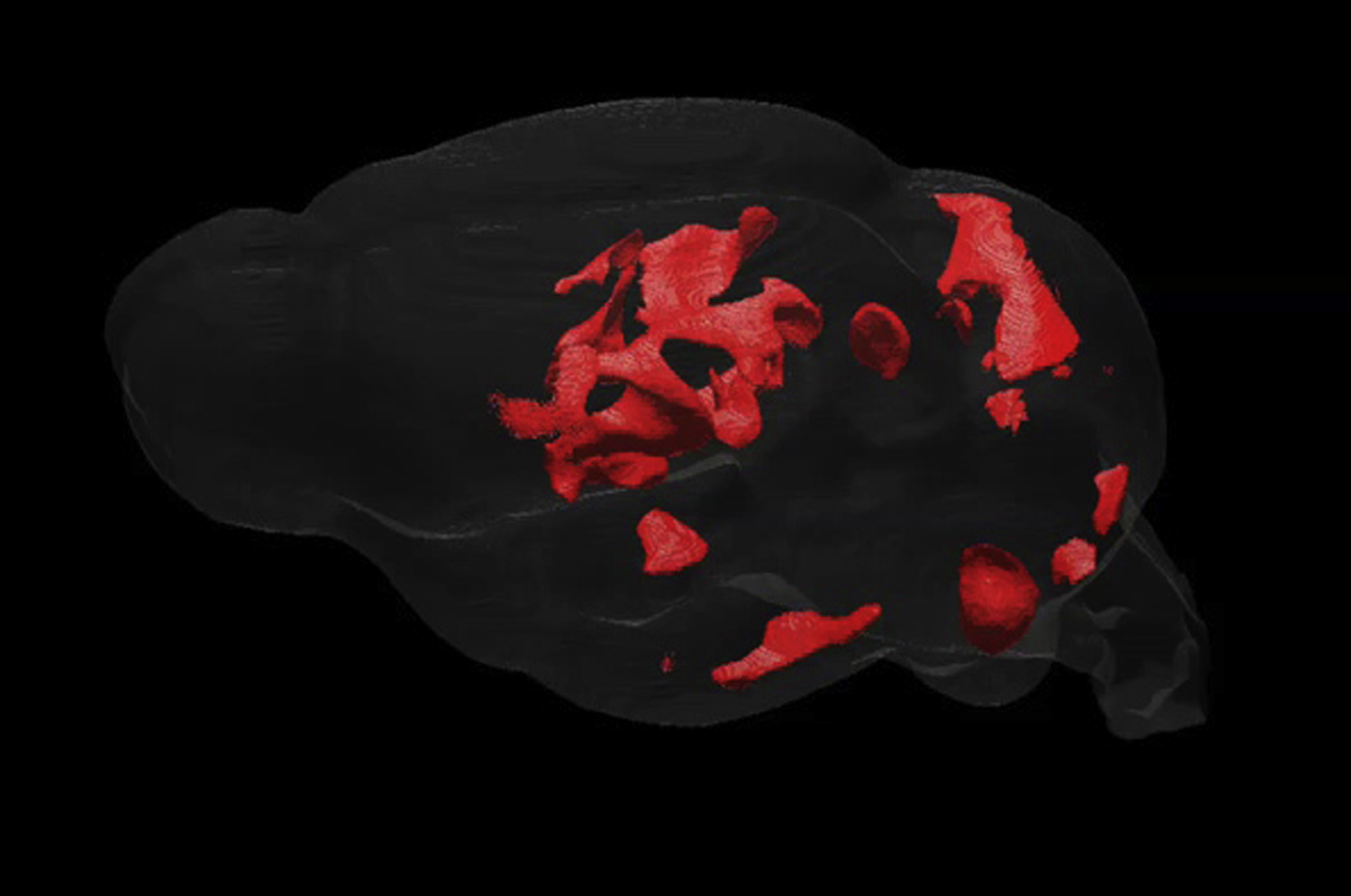

Supplement: Movie S4. 3D Reconstruction of Mouse Brain MRI Volumetrics — Three-dimensional reconstruction of mouse brain MRI with pseudocoloring for significant changes (decreased volume) in Bcl11a+/− mice after normalization for overall brain size. [file mmc7.jpg]

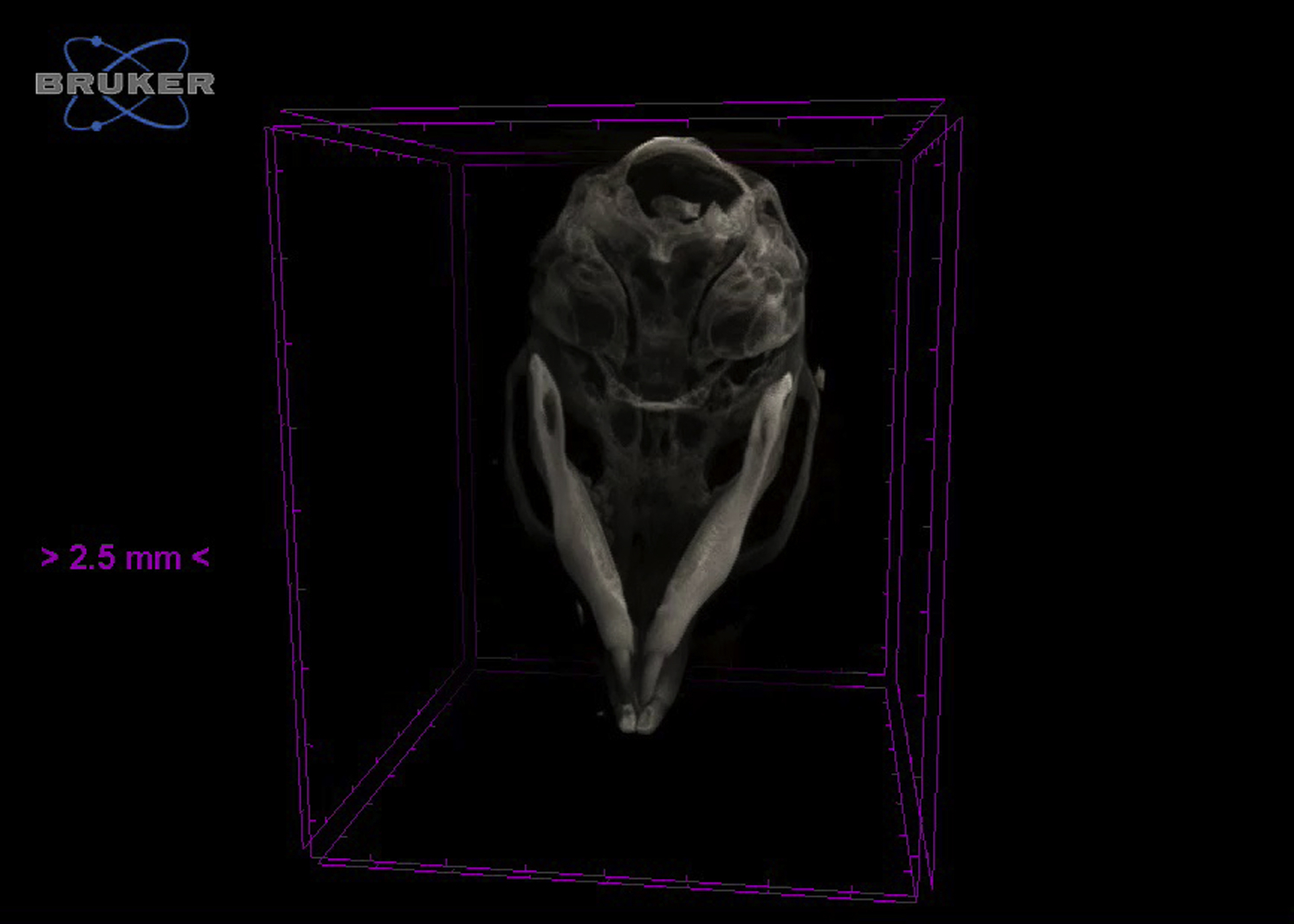

Supplement: Movie S5. 3D Reconstruction of Cranial μCT in a Bcl11a+/− Mouse — The video shows a representative 3D reconstruction of cranial μCT in a Bcl11a+/− mouse. Axis ticks measure 2.5 mm. [file mmc8.jpg]

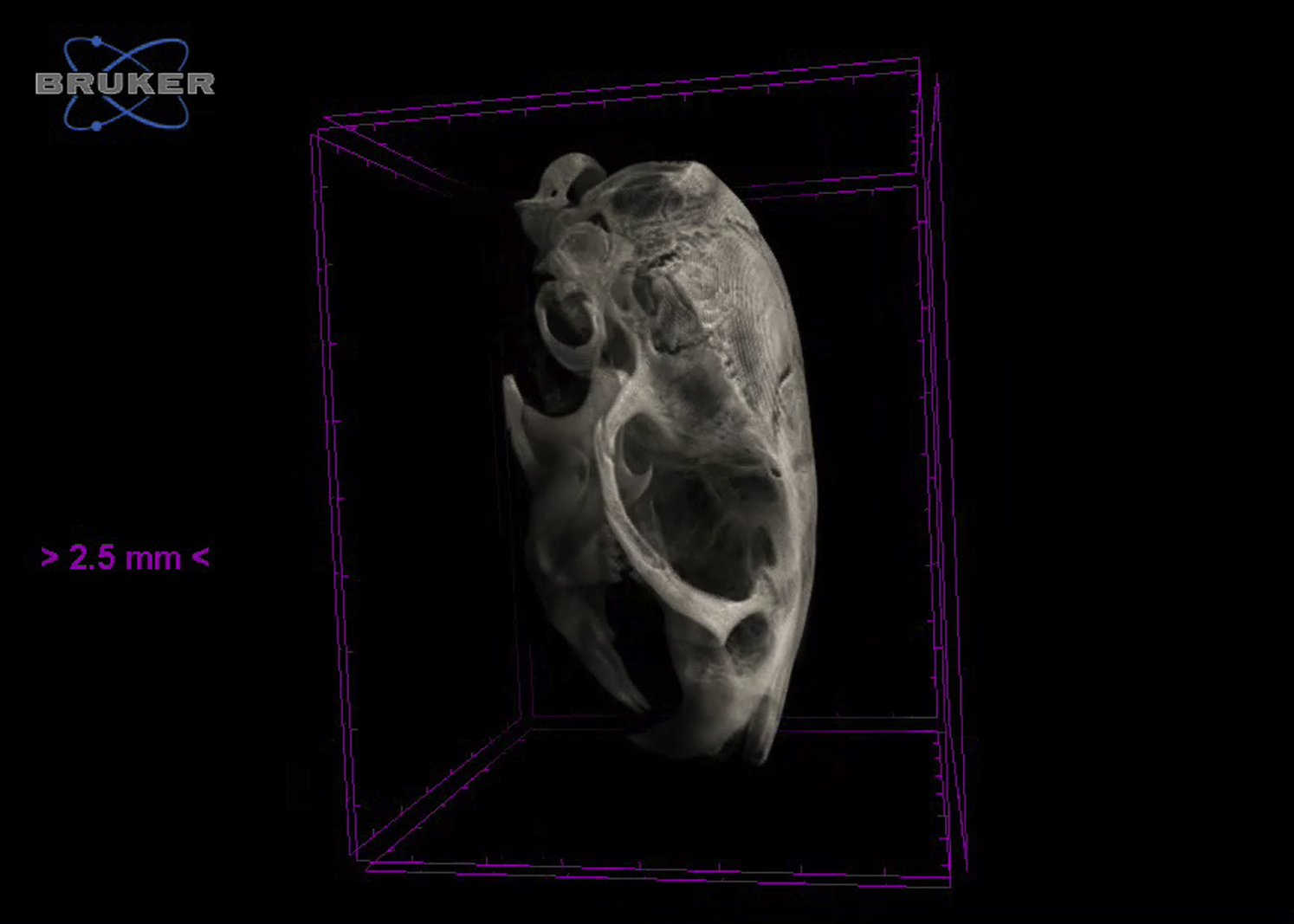

Supplement: Movie S6. 3D Reconstruction of Cranial μCT in a Wild-Type Mouse — The video shows a representative 3D reconstruction of cranial μCT in a Bcl11a+/+ mouse. Axis ticks measure 2.5 mm. [file mmc9.jpg]
