# Supplementary material for: BCL11A Haploinsufficiency Causes an Intellectual Disability Syndrome and Dysregulates Transcription
Source: Am J Hum Genet. 2016 Jul 21;99(2):253–74. doi: 10.1016/j.ajhg.2016.05.030 (PMC4974071; doi:10.1016/j.ajhg.2016.05.030)
Supplement: Document S2. Article plus Supplemental Data [file mmc10.pdf]

BCL11A Haploinsufficiency Causes an Intellectual Disability Syndrome and Dysregulates Transcription

Cristina Dias,[1,17] Sara B. Estruch,[2,17] Sarah A. Graham,[2] Jeremy McRae,[1] Stephen J. Sawiak,[3,4] Jane A. Hurst,[5] Shelagh K. Joss,[6] Susan E. Holder,[7] Jenny E.V. Morton,[8] Claire Turner,[9] Julien Thevenon,[10,11] Kelly Mellul,[12] Gabriela Sánchez-Andrade,[1] Ximena Ibarra-Soria,[1] Pelagia Deriziotis,[2] Rui F. Santos,[13] Song-Choon Lee,[1,14] Laurence Faivre,[10,11] Tjitske Kleefstra,[15] Pentao Liu,[1] Mathew E. Hurles,[1] DDD Study,[1] Simon E. Fisher,[2,16,18,*] and Darren W. Logan[1,19,*]

Intellectual disability (ID) is a common condition with considerable genetic heterogeneity. Next-generation sequencing of large cohorts has identified an increasing number of genes implicated in ID, but their roles in neurodevelopment remain largely unexplored. Here we report an ID syndrome caused by de novo heterozygous missense, nonsense, and frameshift mutations in *BCL11A,* encoding a transcription factor that is a putative member of the BAF swi/snf chromatin-remodeling complex. Using a comprehensive integrated approach to ID disease modeling, involving human cellular analyses coupled to mouse behavioral, neuroanatomical, and molecular phenotyping, we provide multiple lines of functional evidence for phenotypic effects. The etiological missense variants cluster in the amino-terminal region of human BCL11A, and we demonstrate that they all disrupt its localization, dimerization, and transcriptional regulatory activity, consistent with a loss of function. We show that *Bcl11a* haploinsufficiency in mice causes impaired cognition, abnormal social behavior, and microcephaly in accordance with the human phenotype. Furthermore, we identify shared aberrant transcriptional profiles in the cortex and hippocampus of these mouse models. Thus, our work implicates *BCL11A* haploinsufficiency in neurodevelopmental disorders and defines additional targets regulated by this gene, with broad relevance for our understanding of ID and related syndromes.

Introduction

Currently, there are more than 820 genes known to contribute to intellectual disability (ID) and associated childhood neurodevelopmental disorders.[1] Up to 2.5% of individuals are diagnosed with mild to severe ID,[2] characterized by low cognitive ability and impaired adaptive behavior with onset during early development. Genes disrupted in ID and other neurodevelopmental disorders are enriched for those involved in chromatin remodeling and transcriptional regulation.[3,4] Remarkably, more than 1% of cases are attributed to mutations disrupting genes of the BRG1/BRM-associated factor (BAF) swi/snf chromatin-remodeling complex, suggesting that BAFopathies represent an important recurrent cause of ID.[2,5,6]

The Deciphering Developmental Disorders (DDD) study has contributed significantly to the understanding of ID through the identification of novel associated genes on a large scale.[6] The DDD identified de novo missense mutations in *BCL11A* (MIM: 606557, also known as *CTIP1* and *EVI9*) in several individuals with ID. Its protein product has been implicated as a member of the mammalian BAF swi/snf chromatin remodeling complex in human T cells and post-natal mouse brain.[7] BCL11A, a transcriptional factor with C2H2 zinc finger DNA-binding motifs, has been extensively studied for its role in hematopoiesis and malignancy[8–12] and as a transcriptional repressor of fetal hemoglobin,[13] but its contributions to neurodevelopment are more poorly understood.[14–17] A role for BCL11A in normal human brain function is inferred by the presence of chromosomal microdeletions at 2p15–p16.1

[1]Wellcome Trust Sanger Institute, Wellcome Genome Campus, Hinxton CB10 1SA, UK; [2]Language and Genetics Department, Max Planck Institute for Psycholinguistics, PO Box 310, 6500 AH Nijmegen, the Netherlands; [3]Behavioural and Clinical Neuroscience Institute, University of Cambridge, Cambridge CB2 3EB, UK; [4]Wolfson Brain Imaging Centre, University of Cambridge, Cambridge CB2 0QQ, UK; [5]North East Thames Regional Genetics Service, Great Ormond Street Hospital for Children NHS Trust, London WC1N 3JH, UK; [6]West of Scotland Regional Genetics Service, Level 2 Laboratory Medicine Building, Queen Elizabeth University Hospital, Glasgow G51 4TF, UK; [7]North West Thames Regional Genetics Service, London North West Healthcare NHS Trust, Watford Rd, Harrow HA1 3UJ, UK; [8]West Midlands Regional Genetics Service, Birmingham Women's NHS Foundation Trust, Birmingham Women's Hospital, Edgbaston, Birmingham B15 2TG, UK; [9]Peninsula Clinical Genetics Service, Department of Clinical Genetics, Royal Devon and Exeter NHS Foundation Trust, Clinical Genetics Department, Royal Devon & Exeter Hospital (Heavitree), Gladstone Road, Exeter EX1 2ED, UK; [10]Fédération Hospitalo-Universitaire Médecine Translationnelle et Anomalies du Développement (TRANSLAD), Centre Hospitalier Universitaire Dijon, 21079 Dijon, France; [11]Centre de Génétique et Centre de Référence Anomalies du Développement et Syndromes Malformatifs de l'Interrégion Est, Centre Hospitalier Universitaire Dijon, 21079 Dijon, France; [12]Service de Génétique, Hôpital Necker-Enfants Malades, APHP, Institut Imagine, INSERM UMR1163, University Sorbonne-Paris-Cité, 75015 Paris, France; [13]Children's Radiology Department, Royal Manchester Children's Hospital, Manchester M13 9WL, UK; [14]Science Centre Singapore, 15 Science Centre Road, Singapore 609081, Singapore; [15]Department of Human Genetics, Radboud University Medical Center, 6500 HB Nijmegen, the Netherlands; [16]Donders Institute for Brain, Cognition and Behaviour, 6525 EN Nijmegen, the Netherlands
[17]These authors contributed equally to this work
[18]Twitter: @Genes_Speak
[19]Twitter: @darrenlogan
*Correspondence: simon.fisher@mpi.nl (S.E.F.), dl5@sanger.ac.uk (D.W.L.)
http://dx.doi.org/10.1016/j.ajhg.2016.05.030.
© 2016 The Author(s). This is an open access article under the CC BY license (http://creativecommons.org/licenses/by/4.0/).

encompassing the gene in individuals with speech sound disorder[18] and with more severe ID,[19] as well as by a recent association with autism spectrum disorder (ASD).[20] Moreover, biallelic ablation of *Bcl11a* in murine model brains at mid-gestation leads to deficient migration of cortical projection neurons.[16,21] Notably, the microdeletions thus far associated with neurodevelopmental phenotypes have the potential to affect coding regions and non-coding regulatory elements of *BCL11A*[18,19,22,23] and might also have effects on neighboring genes.[24] Therefore, sufficiency of monoallelic *BCL11A* disruption alone has not been demonstrated to be a cause of ID to date, nor has its effects on brain development and regulation been explored.

In addition to missense mutations, we also identified several de novo nonsense and frameshift mutations in *BCL11A* in individuals with ID in the DDD and other cohorts. In this study, we aimed to address the following questions. What are the features of a putative clinical syndrome associated with disruption of *BCL11A*? What are the effects of *BCL11A* missense mutations on functions of the encoded proteins? Are the identified mutations sufficient to cause the clinical phenotype seen in affected individuals? And, as a transcriptional regulator, what are the molecular effects of heterozygous *BCL11A* mutations?

We identify shared clinical features in all individuals, including persistence of fetal hemoglobin (HbF). Our assays in cell-based models support the hypothesis that the missense mutations result in loss of function of the mutated proteins in vivo. Hence, we determine that ID is the result of haploinsufficiency of *BCL11A* through different mutational mechanisms. We show that haploinsufficiency of *Bcl11a* alone in a mouse model is sufficient to recapitulate key cognitive, behavioral, and neuroanatomical phenotypes present in affected individuals. In this haploinsufficient mouse model, we identify transcriptional dysregulation of the hippocampus and cortex, brain regions that correlate with neuroanatomical and behavioral phenotypes.

Together, we present an integrated approach to disease modeling in rare ID syndromes including cellular, behavioral, neuroanatomical, and molecular characterization, and further implicate the BAF complex in neurodevelopmental disorders.

Subjects and Methods

Human Subjects

The Deciphering Developmental Disorders (DDD) study has UK Research Ethics Committee approval (10/H0305/83, granted by the Cambridge South REC, and GEN/284/12 granted by the Republic of Ireland REC). Written informed consent was received from participants prior to inclusion in the study.

The PARI (Regional Action Plan for Innovation) 2011 study was approved by the regional ethics committee and funded by the Regional Council of Burgundy and Dijon University Hospital. Informed consent was received from individuals prior to inclusion in the study.

Where exome sequencing was performed in the context of routine healthcare services, informed consent for the diagnostic procedure was obtained.

Exome Sequencing and Statistical Assessment

Affected individuals were identified through the DDD project (individuals 1–6), through clinical exome sequencing (individuals 7 and 8), and through the Dijon University Hospital PARI 2011 study (individual 9). Whole-exome sequencing and de novo variant annotation were performed as previously described.[6,25] Functional annotation of variants was performed using the Ensembl Variant Effect Predictor (VEP)[26] consequence predictions.

The *BCL11A* de novo variants studied here, together with two loss-of-function de novo variants identified in individuals with ASD,[20] were analyzed for significance in the context of 4,295 DDD study trios, 2,206 clinical exome trios, 50 PARI 2011 trios, and 6,138 trios from other reported exome-sequencing studies of developmental disorders.[20,25,27–36] Statistical assessment of the *BCL11A* variants was performed using an analytical method combining evidence for enrichment for de novo mutations over that expected for gene mutation rate and cohort size,[6] clustering of mutations,[6] and Human Phenotype Ontology (HPO) term similarity between DDD individuals, as described.[37]

Assessment of Enrichment of De Novo Mutations

Expected null mutation rates for *BCL11A* for different functional classes of variants were obtained from reported estimates.[38] The loss-of-function mutation rate was estimated by summing the mutation rates of nonsense, canonical splice sites, and frameshift variants. The functional mutation rate was estimated by summing the loss-of-function mutation rate with the rate of missense variants and in-frame indels. The loss-of-function and functional mutation rates were multiplied by the number of gene transmissions (twice the number of probands) to give the total expected number of mutations given the number of probands sequenced. The expected number of mutations in each class was assumed to be the mean of a Poisson distribution, and the probability of drawing from that distribution a number of mutations equal or greater than the observed number of mutations was calculated. A combined functional de novo statistic was calculated by using Fisher's method to combine p values from enrichment of functional mutations with clustering of missense mutations. The loss-of-function enrichment and the combined functional statistic were compared and the better performing model was selected. The resulting p value was adjusted for the use of two models by Bonferroni correction.

Assessment of Clustering of De Novo Mutations

Exon coordinates and sequences for *BCL11A* were retrieved from Ensembl. Nucleotide mutation rates in trinucleotide contexts were provided by Kaitlin Samocha and Mark Daly. De novo missense mutations were randomly sampled, weighted by the context-specific mutation rates, matching the number of sampled mutations to the number of known de novos. The de novos were assessed for their tendency to cluster within close proximity to each other. The proximity was calculated as the geometric mean coding distance between all the possible de novo pairs. The expected distribution of proximity was assumed as the distribution of proximity for sampled de novos from 1,000,000 simulations. The p value was estimated as the proportion of simulated proximities less than or equal to the observed proximity.

Assessment of Phenotypic Similarity

Probands in the DDD study had phenotypes systematically recorded by clinical geneticists using terms from the Human Phenotype Ontology (HPO). For pairs of HPO terms, we determined the information content (IC) for the most informative common ancestor of the two terms. The IC was calculated as the negative logarithm of the probability of the terms' usage within the 4,295 DDD probands (including descendant terms' usage). The similarity of terms between two individuals was estimated as the maximum IC from pairwise comparisons of the individuals' HPO terms. The score for a set of n probands was estimated as the sum of all the pairwise scores. The null distribution was simulated by randomly sampling 100,000 sets of n probands and calculating scores as above. The p value was estimated as the proportion of simulated scores greater than or equal to the observed score.

DNA Constructs

The coding sequences of *BCL11A*-S (GenBank: NM_138559), *BCL11A*-L (GenBank: NM_018014), and *NONO* (GenBank: NM_001145408) were amplified from human fetal brain cDNA using the primers in Table S1 and cloned into pCR2.1-TOPO (Invitrogen). The missense mutations were introduced using the QuikChange Lightning SDM kit (Agilent) and the primers in Table S2. For expression of fusion proteins with *Renilla* luciferase, YFP, and mCherry, cDNAs were subcloned into the pLuc, pYFP, and pmCherry expression vectors, respectively, which have been described previously, using the BamHI and XbaI sites.[39,40] For the mammalian one-hybrid assay, a vector for expression of BCL11A fused in frame with the yeast GAL4 DNA-binding domain was created by cutting and re-ligating pBIND (Promega) at the ClaI sites to remove the *Renilla* luciferase expression cassette. Wild-type and mutant forms of *BCL11A*-L were subcloned into the BamHI and XbaI sites of this vector. A reporter plasmid was generated by inserting a KpnI-NcoI fragment of pG5luc (Promega) containing five GAL4 binding sites and a minimal adenovirus major late promoter into the vector pGL4.23 (Promega), which contains a codon-optimized firefly luciferase gene. A plasmid containing Renilla luciferase downstream of the herpes simplex virus thymidine kinase promoter (pGL4.74, Promega) was used for normalization.

Cell Culture

HEK293 cells (ECACC cat# 85120602, RRID: CVCL_0045) were cultured in DMEM supplemented with 10% FBS. Transfections were performed using GeneJuice (Merck-Millipore) according to the manufacturer's instructions.

Western Blotting

Cells were transfected in 6-well plates and cultured for 48 hr. Cells were lysed for 10 min at 4°C with 100 mM Tris (pH 7.5), 150 mM NaCl, 10 mM EDTA, 0.2% Triton X-100, 1% PMSF, and protease inhibitor cocktail. Cell lysates were cleared by centrifugation at 10,000 × *g* for 3 min at 4°C. Proteins were resolved on 10% SDS-polyacrylamide gels and transferred to PVDF membranes using a TransBlot Turbo blotting apparatus (Bio-Rad). Membranes were blocked in phosphate-buffered saline containing 5% non-fat milk powder and 0.1% Tween-20 and then incubated overnight at 4°C with primary antibody. The following antibodies were used: anti-GFP (Clontech cat# 632380, RRID: AB_10013427; 1:8,000, for YFP constructs) and anti-β-actin (Sigma cat# A5441, RRID: AB_47644; 1:10,000). After washing, membranes were incubated with horseradish peroxidase-conjugated goat anti-mouse or anti-rabbit IgG for 45 min at room temperature. Proteins were visualized using Novex ECL Chemiluminescent Substrate Reagent Kit (Invitrogen) and a ChemiDoc XRS+ imaging system (Bio-Rad).

Cellular Assay Fluorescence Microscopy

Cells were seeded on coverslips coated with poly-L-lysine. Cells were cultured for 30 hr post-transfection and then fixed with methanol. Fluorescence images were acquired using Zeiss Axiovert A-1 or Axio Imager 2 fluorescence microscopes with ZEN Image software.

BRET Assay

The BRET assay has been described in detail elsewhere.[40] In brief, cells were transfected in white, clear-bottomed 96-well plates, in triplicate, with 6 fmol *Renilla* luciferase fusion expression plasmid and 6 fmol YFP fusion expression plasmid (total mass of DNA was adjusted to 60 ng with filler plasmid). *Renilla* luciferase or YFP with a nuclear localization signal were used as controls. Cells were cultured for 48 hr post-transfection. Enduren luciferase substrate (Promega) was added at a final concentration of 60 μM, and cells were cultured for a further 4 hr. Luminescence was measured in a TECAN Infinite F200PRO microplate reader using the Blue1 and Green1 filters.

Mammalian One-Hybrid Assay

Cells were transfected in white, clear-bottomed 96-well plates, in triplicate, with 8.5 fmol pBIND-BCL11A, 5 fmol firefly luciferase reporter plasmid, and 2 fmol *Renilla* luciferase normalization plasmid (total mass of DNA was adjusted to 60 ng with filler plasmid). Cells were cultured for 48 hr post-transfection. Firefly and *Renilla* luciferase activities were measured using the Dual Luciferase Reporter Assay (Promega).

Animal Models

Housing and breeding of mice and experimental procedures were carried out under the authority of a UK Home Office license (80/2472), after review by the Animal Welfare and Ethical Review Body of the Wellcome Trust Sanger Institute.

Molecular, behavioral, and imaging studies were performed using mutant mice harboring a *Bcl11a* gene trap upstream of exon 4, disrupting all major isoforms: knockout first, conditional-ready LacZ reporter allele ($Bcl11a^{LacZ}$; Figures S1A and S1B).[12,41] Mice were kept on a C57BL/6(50%);129S5(50%) background. Immunohistochemistry was performed on F1 mice from a cross between $Bcl11a^{+/LacZ}$ mice and mice harboring the $\mathrm{Bcl11a^{tm1Peli}}$ eGFP reporter allele ($\mathrm{Bcl11a^{+/tm1Peli}}$ and $Bcl11a^{LacZ/\mathrm{tm1Peli}}$).[42] All mice used were from colonies maintained at the Research Support Facility of the Wellcome Trust Sanger Institute.

Imaging

Adult mice (aged 16 ± 1 week) were anesthetized and then transcardially perfused with 20 mL of ice-cold PBS followed by 4% paraformaldehyde (PFA). The skull was detached and skin removed. The brain was kept in loco. Skulls were transferred into PBS after 24 to 48 hr and kept at 4°C before imaging.

MRI Image Acquisition

Brains were scanned using a Bruker PharmaScan 47/16 system at 4.7T with a manufacturer-provided birdcage transmit-receive coil. The imaging protocol was fast spin echo (scan parameters: repetition time 2,000 ms, effective echo time 16 ms, echo train

length 4, bandwidth 32 kHz, matrix 256 × 192 × 128, field of view 1.79 × 1.34 × 0.90 cm^3, resolution 70 μm isotropic with two averages).

MRI Tensor-Based Morphometry

Brains were segmented into gray and white matter portions and registered using the SPMMouse toolbox[43] with SPM8 (Wellcome Trust Centre for Neuroimaging, University College London) and the DARTEL registration toolbox.[44] Jacobian determinants from the registration process were smoothed with a 400 μm Gaussian kernel and tested with an *F*-test between groups to produce voxel-wise maps for tensor-based morphometry. The scaling factor from the affine matrix was used as a covariate to find differences in volume that could not be explained by overall brain size. To control the type I error rate due to multiple comparisons, an adjusted p value was used for a false-discovery rate at $p < 0.05$.

μCT

Image acquisition and reconstruction were performed on the Skyscan 1172 high-resolution micro-CT (μCT) using the standard software provided by the manufacturer (Bruker micro-CT). 3D image and video generation was performed with DataViewer software (Brucker, v.1.5.1.2, May 27, 2014) using the same parameters for all skulls (opacity adjusted, luminance adjusted, 96% red, 90% green, 84% blue, 5% shadows, 100% emission, 50% diffuse, 25% specular, 40° camera viewing). Measurements were obtained blinded to genotype as described by de Carlos et al.[45] and corrected using a multiplanar visualization of reference points in sagittal, axial, and coronal planes (described in Figure S2). Comparison of lengths and statistical analyses (Mann-Whitney test) were performed using GraphPad Prism v.6.00 (GraphPad Software).

Histology and Immunohistochemistry

All brain tissue samples were fixed through transcardial perfusion of 4% PFA as described above. Samples were dehydrated, paraffin embedded, and sectioned before hematoxylin and eosin, luxol fast blue, and cresyl violet staining using standard histological techniques.

For immunohistochemistry, perfused brain samples were cryoprotected in 30% sucrose, embedded in OCT, and stored at −80°C. Samples were sectioned at 16 μm using a LeicaCM3050S cryostat. Sections were permeabilized with 1% Triton X and blocked with BlockAid Blocking Solution (Thermo Fisher Scientific cat# B10710) or rabbit serum (β-gal). Specificity of BCL11A staining was confirmed by co-detection of β-gal and GFP (Figure S3). Because endogenous GFP expression was below detection, a specific primary antibody was utilized. Primary antibodies used were anti-BCL11A (Abcam cat# ab19489, RRID: AB_2063996; 1:250), anti-β-gal (Abcam cat# ab9361, RRID: AB_307210; 1:1,000), and anti-GFP (Torrey Pines Biolabs cat# TP-401, RRID: AB_10013661; 1:500). Secondary antibodies included Alexa Fluor-564 goat anti-mouse-IgG1 (Thermo Scientific cat# A-21123, RRID: AB_2535765), Alexa Fluor-488 goat anti-rabbit IgG (Thermo Scientific cat# R-37116, RRID: AB_2556544), and Alexa Fluor-488 rabbit anti-chicken-IgY (Jackson Immuno Research cat# 303-545-003, RRID: AB_2339327). Slides were mounted with ProLong Gold Antifade reagent with DAPI nuclear counterstain (Life Technologies cat# P36935). Sections were visualized and photographed on a Leica TCS SP5/DM6000 confocal microscope with Leica Application Suite Advanced Fluorescence software or a Zeiss Axiovert 200M microscope with Axiovision software.

Social Recognition Assay

For all behavioral assays described, mice were habituated to the behavior test room for ≥1 hr under same light conditions as the test. For the social recognition assay,[46] group-housed test mice (mutant and littermate wild-type controls) were habituated to the 39 × 21 cm test arena for 10 min. On day 1 (habituation-dishabituation test), a conspecific anesthetized stimulus was placed on the center of the test arena for 1 min, repeated 4 times at inter-trial intervals of 10 min (Movie S1). On the 5th trial, a new stimulus mouse was presented. On day 2, after a 24 hr interval, the discrimination test was performed (Figure S4). The familiar stimulus animal from trials 1–4 and a new unfamiliar mouse were placed on opposite sides of the test arena for 2 min (Movie S2). The amount of time the test animal spent investigating by close-proximity sniffing, oronasal contact, or approaching within 1–2 cm was recorded. For day 2, social discrimination preference index was calculated according to the following equation: preference index (PI) = (investigation time [s] of novel unfamiliar stimulus − familiar stimulus)/(investigation time [s] of familiar + unfamiliar stimulus).

Trials were performed under red light and recorded with an overhead camera. The trials were scored blind to genotype by two observers, and the average of both observations was used as the time. Stimulus animals were subject to non-terminal anesthesia with ketamine/xylazine (i.p. 1 g/0.1 g per kg of body weight). Familiar stimuli were recovered with atipamezole for use in the 24 hr discrimination test. The stimulus animals were gender matched, equal or lower weight, and different strain. 129P2/OlaHsd,129S5/SvEvBrd mice were used for trials1–4 and 24 hr discrimination familiar stimulus; C57BL/6 or C57BL/6;129 mice from different breeding colonies were used for trial 5 and 24 hr discrimination unfamiliar stimulus. Two-way ANOVA and unpaired t test (after a D'Agostino & Pearson omnibus normality test) were employed where indicated. Statistical analysis for all mouse behavior experiments was performed with GraphPad Prism v.6.00 (GraphPad Software).

Open Field Assay

Mice were placed in a 37 × 37 cm open field for 5 min under red light. Their movements were tracked by detection of the mouse center point using overhead infrared video cameras and automated video tracking software (Ethovision XT 8.5, Noldus Information Technology). A 24 × 24 cm "center zone" was designated with equidistant borders to the open field walls. The frequency and time each mouse was within this center zone was recorded. A period of movement was defined when the mouse reached a velocity of 2 cm/s over two frames; a period of non-movement was defined when velocity was lower than 1.75 cm/s over two frames.

Three-Chamber Social Behavior

The three-chamber social approach task was performed under red light, modified from Yang et al.[47] The chamber layout is presented in Figure S4F. In brief, a test arena was divided into four quadrants; one quadrant was sealed off, and the remaining three chambers formed the L-shaped arena used for the test. Innate chamber side preference was controlled for during the habituation phase and did not show difference between genotypes (t test, $p = 0.2144$). Two arenas were used for increased throughput of behavior experiments, and mice were randomly assigned to the test arena. Differences between arenas were tested. In one arena, mice showed an innate preference for the left chamber (0.15

increase in preference index in habituation) independent of genotype (2-way ANOVA, per genotype $p = 0.2004$; per arena $p = 0.0262$). Subsequent times were normalized to the innate preference index. Mouse movements were tracked via overhead infrared video cameras and automated video tracking software.

Test mice were habituated to the center chamber for 5 min. Doors were then opened to the two empty side chambers and mice were allowed to explore all three chambers for a further 5 min. Doors were re-closed and the mice were contained briefly in the center chambers as objects were placed in the side chambers. Identical objects—stainless steel cylindrical containers with holes sufficiently large to contain a mouse nose—were placed upside down in the side chambers. One object contained a live conspecific (novel sex-matched stimulus previously habituated to the object) that could freely move inside the object. Test mice then explored all three chambers for 10 min with automated movement tracking (Movie S3). The preference index was calculated as follows: PI = (time in chamber with object containing a conspecific stimulus − time in chamber with object only)/(time in chamber with object containing a conspecific stimulus + time in chamber with object only).

RNA-Sequencing Processing and Analysis

Mice were sacrificed at 16 weeks of age. The cortex and hippocampus were dissected from male animals, snap frozen in liquid nitrogen, and stored at −80°C. Tissue was homogenized in buffer RLT plus with β-mercaptoethanol (10 μL/mL) using the QIAGEN TissueLyser LT. RNA was pre-treated on gDNA eliminator columns and then extracted on RNeasy Plus columns as per manufacturer's protocol (QIAGEN). Multiplexed libraries were prepared for sequencing using Illumina RNA Library Preparation Kits as per manufacturer's protocol. Paired end sequencing was performed on the Illumina HiSeq 2000 or V4 generating 75 bp reads.

Using STAR v.2.4,[48] sequenced reads were realigned to an altered version of the mouse reference genome GRCm38 (Ensembl annotation release 78, December 2014) containing a pseudo-chromosome with the mutant neomycin cassette sequence for genotype. The number of reads mapped to each gene was counted using the HTSeq (v.0.6.1) count function in mode intersection-nonempty. HTSeq count data was used as input for differential gene expression analysis using the R (v.3.2.2) DESeq2 package (v.1.9.29).[49] Because samples were sequenced in three different sequencing experiments (including both genotypes per batch), the R/Bioconductor sva package (v.3.16.0) was used to estimate the technical variation (see Web Resources). The covariates ("surrogate variables" generated by sva) were added into the DESeq2 test design. DESeq2 uses the Benjamini-Hochberg procedure to control for multiple testing, returning an adjusted p value (padj) for the differential gene expression. Given the exploratory nature of the analysis, the DESeq2 default significance cut-off of BH-adjusted p value < 0.1 was used.

The top most highly expressed mitochondrial genes were excluded from the normalized read count generation in DESeq2, because these were highly expressed and variable between biological replicates (Ensembl: ENSMUSG00000064351, ENSMUSG00000064370, ENSMUSG00000064341, ENSMUSG 00000064339, ENSMUSG00000064367, ENSMUSG00000064337, ENSMUSG00000064363). For the hippocampal tissues, two wild-type and three *Bcl11a*$^{+/-}$ samples were found to be extreme outliers on principal component analysis and were excluded from the differential gene expression analysis. Six samples per genotype were used for the hippocampus and nine samples per genotype for the cortex.

Gene Ontology

Gene ontology enrichment analysis was performed with GeneTrail.[50] Over-representation analysis of the differentially expressed genes was performed using parameters in Table S3 with significance threshold, 0.1 and p values adjusted for multiple testing using the Benjamini-Hochberg FDR adjustment. In all analyses a background comprised of only the expressed genes used for the relevant analysis was provided.

Gene Enrichment Analysis

Enrichment for voltage gated ion channels (VGIC) was performed using genes annotated as one of the 141 mouse VGIC in the IUPHAR/BPS database (accessed 30 September 2015).[51] ASD-related genes were selected from the SFARI gene web portal.[52] Of the 740 genes downloaded from The Human Gene Module of SFARI gene database (September 2015), 485 were selected based on exclusion of genes with the following levels of evidence annotated in the SFARI database: functional (only), functional negative association (>1), genetic association (only). Of these, 481 mouse orthologs were identified through Ensembl.[53] We generated a second subset of 75 unique human genes and 1 pseudogene from the Gene Scoring Module of SFARI, which were annotated in the following categories defined in the database: S (syndromic), 1 (high confidence), and 2 (strong candidate). 75 mouse orthologs were identified through Ensembl. Analysis was performed in R v.3.2.2.; for statistical analysis, hypergeometric test was employed with a significant p value < 0.05. Genes used in gene enrichment analyses above are available in Table S4.

Transcriptome Assembly

For a qualitative analysis of *Bcl11a* isoform and transcription start site usage, the paired end reads aligned using STAR v.2.4.0 were assembled into transcripts via Cufflinks v.2.2.1.[54] Assembled gtf files from different replicates for each tissue were merged via cuffmerge including the reference annotation. Cuffquant was employed for isoform quantification. Data visualization and graphical representation were performed with CummeRbund v.2.12.0.[54]

Results

Identification of Mutations in BCL11A in Individuals with ID

We identified nine individuals with intellectual disability with de novo mutations in *BCL11A*: three missense and six loss of function (LoF). In the DDD study[6] we identified six individuals with de novo heterozygous variants in *BCL11A*, from a total of 4,295 affected individuals studied using whole-exome sequencing (Figure 1A; Table 1). Three missense variants were first identified; they cluster together in exon 2 of *BCL11A* (individuals 1, 2, and 3).[14,15] Subsequently, we identified three novel variants (one nonsense [individual 4] and two frameshift [individuals 5 and 6]) classified[26] as LoF (Figure 1C). Individual 6 also has a probably pathogenic 4.3 Mb duplication but

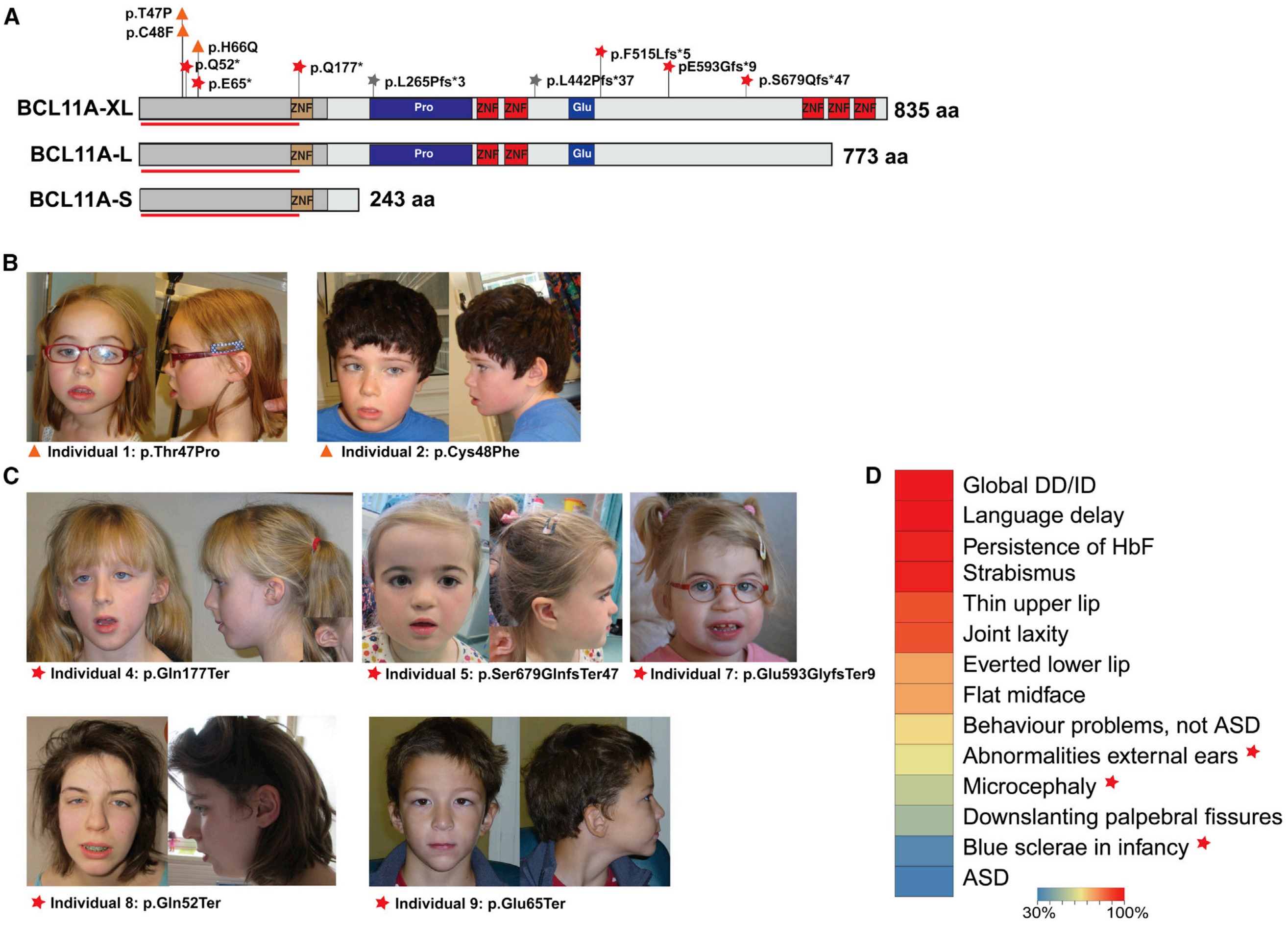

Figure 1. Clinical and Molecular Features of Individuals with *BCL11A* Mutations

(A) Schematic representation of the three major isoforms of BCL11A (GenBank and Ensembl transcript identifiers indicated): BCL11A-XL (NM_022893.3, ENST00000335712), BCL11A-L (NM_018014.3, ENST00000356842), and BCL11A-S (NM_138559, ENST00000359629). Mutations are represented on and annotated according to the BCL11A-XL isoform (NM_022893.3); predicted protein variants are represented as follows: orange triangles, missense variants; red stars, truncating variants identified in the current study; gray stars, variants in previously reported individuals with autism spectrum disorder. C2H2 DNA binding zinc finger domains are represented in red; non-DNA binding zinc finger is shaded brown. The red line indicates the putative dimerization region. Putative region required for SUMO1 recruitment is shaded gray. Proline (Pro)- and glutamate (Glu)-rich regions are shaded blue. Abbreviation is as follows: aa, amino acids.

(B–D) Clinical features of individuals 1, 2, 4, 5, 7, 8, and 9. Individual number and predicted protein variant are indicated below the respective photograph. Various and partly overlapping facial features of individuals with missense (B) and loss-of-function (C) mutations in *BCL11A*, including strabismus, downslanting palpebral fissures, synophrys, flat midface, thin upper lip, and full lower lip. Shared core clinical features of all affected individuals (detailed in Table 1) are summarized in (D). The heatmap colors represent the frequency of the feature seen, from 100% (red) to 30% (blue); frequency is calculated based on information available for each feature. Features that are present predominantly (microcephaly, abnormal external ears) or exclusively (blue sclerae in infancy) in individuals with LoF mutations are indicated by a red star. Abbreviations are as follows: DD, developmental delay; ID, intellectual disability; ASD, autism spectrum disorder.

was unavailable for further investigation (Table 1). Additional individuals with de novo LoF variants were identified in other developmental disorder cohorts (individuals 7–9). To evaluate their pathogenicity, we performed a statistical enrichment analysis of all these variants, along with two further LoF variants recently identified in ASD[20,36] (individuals 10 and 11; Table 1). We find compelling statistical support for an excess of mutations in *BCL11A* ($p = 6.4 \times 10^{-15}$; see Subjects and Methods).

All individuals that we identified with *BCL11A* mutations presented with global delay in developmental milestones, including speech and language delay. Most individuals exhibited moderate ID, though cognitive dysfunction varies from mild (individual 1) to severe (individual 2) (Table 1). One of the two individuals recently ascertained through an ASD study[20,36] also has severe ID (individual 10); intellectual capacity was not reported in the other. Individual 2 has likewise received a diagnosis of ASD, and four other individuals present a spectrum of behavior abnormalities including repetitive behavior and sensory problems. Shared physical features among affected individuals (Figure 1D) include joint laxity (87%), strabismus

(100%), microcephaly (55%), and thin upper lip and flat midface (Figures 1B and 1C). 62% of affected individuals have external ear abnormalities (Table 1), which are more severe in individuals with nonsense and frameshift mutations. Individuals 4, 5, and 6, all of whom present nonsense or frameshift mutations, had blue sclerae in infancy. As well as the *BCL11A* variant, individual 6 also carries a 15q15.3q21.1 duplication, and a contribution of this duplication to the phenotype, specifically to delayed skeletal maturation and short stature, cannot be excluded.[55] HbF was significantly elevated in all affected individuals in whom it was assessed, including all those with missense mutations in *BCL11A* (Figure 1D, Table 1). Notably, individual 1, carrying a missense mutation, had HbF levels similar to those seen for individual 5, carrying a frameshift mutation.

All LoF variants are predicted to deleteriously affect isoforms L (GenBank: NM_018014.3, Ensembl: ENST00000356842) and XL (NM_022893.3, ENST00000335712) of BCL11A (Figure 1A) through premature truncation or frameshift and nonsense-mediated decay (annotated isoforms are summarized in Figure S5). All missense variants are located in the N-terminal region of BCL11A, which is required for homo- and heterodimerization of BCL11A isoforms,[56] as well as for interaction with repressive nucleosome remodeling complexes.[57] Given the known role of the gene as a regulator of stage-specific hemoglobin expression,[13] we hypothesized that the missense mutations impair function of BCL11A and investigated functional consequences using cell-based assays.

Missense Mutations Disrupt BCL11A Function

Our cellular assays focused on BCL11A-L and BCL11A-S (GenBank: NM_138559, Ensembl: ENST00000359629), the two isoforms of BCL11A reported in human brain.[10] We generated mCherry and YFP-tagged versions of these isoforms containing each of the three missense mutations identified in affected individuals and expressed them in HEK293 cells (Figure 1A). BCL11A-L binds to DNA in a sequence-specific manner via two C2H2 zinc finger domains (Figure 1A)[8] and localizes to the nucleus. BCL11A-S lacks the zinc finger domains and is unable to bind to DNA, but can form heterodimers with BCL11A-L and -XL that mediate the translocation from its predominantly cytoplasmic localization into the nucleus.[8,14,15,56] BCL11A-L has predominant nuclear localization, whereas isoform S localizes to the cytoplasm in the absence of interaction with L and XL isoforms.[56]

The mutant forms of BCL11A-L and BCL11A-S show similar protein levels to the wild-type (Figure S6). We found that both wild-type and mutant BCL11A-S isoforms are predominantly localized to the cytoplasm (Figure 2A).[8,15,58] In contrast, wild-type BCL11A-L is found in nuclear paraspeckles, as demonstrated by its co-localization with the paraspeckle-specific protein NONO,[56] also recently implicated in ID.[59] Strikingly, all three missense mutations disrupt the paraspeckle distribution of BCL11A-L, as well as its co-localization with NONO (Figures 2B and 2C). In addition, using a bioluminescence resonance energy transfer (BRET) assay, we found that the mutations reduce, but do not completely abolish, the interaction of BCL11A-L with NONO (Figure 2D).[40]

The three missense mutations identified in affected individuals all lie within a region encoding a putative dimerization site in BCL11A (Figure 1A).[8] We confirmed that wild-type BCL11A-L and BCL11A-S form homo- and heterodimers (Figures 3A, 3B, S7A, and S7B)[15,56] and that co-expression of BCL11A-L causes BCL11A-S to translocate from the cytoplasm into nuclear paraspeckles (Figure 3C).[56] We found that BCL11A-L isoforms carrying mutations have a substantially reduced interaction with both L and S wild-type BCL11A isoforms (Figures 3A and 3B) and a reduced capacity to translocate wild-type BCL11A-S into the nucleus (Figure 3C). Similar effects were observed when the mutations were introduced into BCL11A-S (Figure S7).

We used a mammalian one-hybrid assay to examine the effects of the missense mutations on the capacity of BCL11A to regulate transcription. BCL11A-L was fused to the DNA-binding domain of yeast GAL4 and co-transfected with a reporter plasmid containing five sequential GAL4 binding sites upstream of a luciferase gene. Wild-type BCL11A-L produced a ~2.5-fold activation of reporter transcription, an effect that was significantly reduced for all three mutant isoforms ($p < 0.05$, one-way ANOVA followed by Bonferroni post hoc test; Figure 3D).

In summary, all three missense mutations identified in affected individuals are associated with persistence of HbF and show consistent deleterious effects on multiple aspects of BCL11A molecular function, including localization, dimerization, and transcriptional regulatory activity. These findings indicate that the mutations yield a loss of function of the mutant proteins in vivo, suggesting that the associated neurodevelopmental syndrome may result from a haploinsufficiency mechanism.

Mice with *Bcl11a* Haploinsufficiency Phenocopy Features of the Human ID Syndrome

To test the hypothesis that haploinsufficiency of *BCL11A* is sufficient to cause a specific neurodevelopmental syndrome, and to gain further insights into pathogenic mechanisms, we generated a *Bcl11a* heterozygous mouse line containing a LacZ reporter (Figure S1, herein termed $Bcl11a^{+/-}$; see Methods).[12] Mouse *Bcl11a* (also known as *Evi9* and *Ctip1*) is highly homologous to human *BCL11A* (the XL isoform shows 97% cDNA sequence identity and 100% protein sequence identity between species).[53] Germline biallelic loss of *Bcl11a* leads to perinatal lethality in mouse models,[9] though its neurodevelopmental phenotype has not been explored. In utero knockdown experiments and conditional knockout models show that *Bcl11a* is necessary for normal mouse cortical development.[9,16] However, global haploinsufficiency of *Bcl11a* in

Table 1. Genetic and Clinical Characteristics of Individuals with De Novo *BCL11A* Mutations

**Individual**	**1**	**2**	**3**	**4**	**5**	**6**[a]	**7**	**8**	**9**	**10**[b]	**11**[c]	**Summary of Features**
BCL11A mutation[d]	c.139A>C (p.Thr47Pro)	c.143G>T (p.Cys48Phe)	c.198C>A (p.His66Gln)	c.529C>T (p.Gln177Ter)	c.2035_2037delinsC (p.Ser679GlnfsTer47)	c.1545delinsGGCTTC (p.Phe515LeufsTer5)	c.1775_1776insTGG CTCAGCGG (p.Glu593GlyfsTer9)	c.154C>T (p.Gln52Ter)	c.193G>T (p.Glu65Ter)	c.1325_1325del (p.Leu442ProfsTer37)	c.792_793insC (p.Leu265ProfsTer3)	3 missense; 8 nonsense/ frameshift
Decipher ID	262471	262952	261658	268026	275695	280953	NA	NA	NA	NA	NA	
Mutation class	missense	missense	missense	loss of function	loss of function	loss of function	loss of function	loss of function	loss of function	loss of function	loss of function	
Sex	F	M	F	F	F	F	F	F	M	F	M	3 M, 8 F
Microcephaly	–[e]	–[e]	+	+	+	+	+	–[e]	–[e]	NA	NA	5/9
Intellectual disability	mild-moderate	moderate-severe	moderate	moderate	moderate	+[f]	moderate	moderate	moderate	severe	NA	10/10 (average moderate)
**Developmental Milestones (Age of Achievement in Months)**												
Sat independently	7	10	12	NA	11	NA	NA	14	12	NA	NA	~11
Walked independently	20	36	24	22	45	NA	NA	23	36	30	NA	~29.5
First words	22	27	36	16	NA[g] (80 words at 100 months)	24–30	NA (few words at 33 months)	60	36	NA (2 words at 6 years)	NA	~32
**Craniofacial Features**												
Downslanting palpebral fissures	+	–	–	+	–	NA	+	+	–	NA	NA	4/8
Epicanthus	–	–	–	–	+	NA	+	–	+	NA	NA	
Strabismus	+	+	+	+	+	NA	+	+	+	NA	NA	8/8
Blue sclera in infancy	–	–	–	+	+	+	–	–	–	NA	NA	3/9
Flat midface	+	+	+	+	+	NA	–	–	+	NA	NA	6/8
Thin upper lip	+	+	+	+	+	NA	–	+	+	NA	NA	7/8

(*Continued on next page*)

Table 1. *Continued*

Individual	1	2	3	4	5	6[a]	7	8	9	10[b]	11[c]	Summary of Features
Everted lower lip	+	+	–	+	–	NA	+	+	+	NA	NA	6/8
Nose	anteverted	small nares	anteverted; full tip	–	–	NA	full tip	small nares; full tip	small nares; full tip	NA	NA	4/8
Micro/ retrognathia	–	–	–	–	–	retro	–	retro	micro	NA	NA	3/9
Additional craniofacial features	frontal upsweep, cleft uvula	coarse hair	–	small mouth,[h] high palate, pointed chin	small mouth, plagiocephaly, synophrys	–	large tip of the nose, broad bridge, flared eyebrows, telecanthus	large tip of the nose, high palate	high nasal bridge	NA	NA	
External ear anomalies	–	–	+[i]	+[j]	+[k]	NA	+[l]	–	+[m]	NA	NA	5/8
**Additional Physical and Neurologic Features**												
Joint hypermobility	+	–	+	+	+	NA	+	+	+	NA	NA	7/8
Short stature	–	–	–	–	–	+	+	–	–	NA	NA	2/9
Gait abnormalities	broad based	broad based, truncal ataxia	–	–	–	NA	–	–	ataxia	NA	NA	3/8
Other	anteriorly placed anus, dyspraxia		fetal pads, bilateral coxa valga, valgus foot deformity		hernia repair	congenital hip dislocation, delayed bone age	GE reflux	large 2nd metacarpals, scoliosis	pectus excavatum	NA	NA	
**Behavioral Features**												
ASD	–	+	–	–	–	NA	–	–	–	+	+	3/10
Repetitive behavior	+	+	+	+	–	NA	–	–	–	–	NA	4/9
Other behavior problems	emotional lability	recurrent hand flapping	sensory abn., self-injurious behavior	recurrent hand flapping/ biting	none reported	NA	none reported	anxiety, eating disorder	none reported	attention deficit	NA	6/9
Sleep disturbance	–	+	+	+	–	NA	–	–	–	+	NA	4/9

(*Continued on next page*)

Table 1. *Continued*

Individual	1	2	3	4	5	6[a]	7	8	9	10[b]	11[c]	Summary of Features
**Additional Investigations**												
MRI	slightly reduced WM volume	small CV	NA	normal	VM	NA	NA	atrophy of the superior CV	mild hypoplasia of the CC	NA	NA	
Hemoglobin F %	20.8%	8%	8.7%	NA	26.3%	NA	NA	3.1%	8.6%	NA	NA	6/6

Abbreviations are as follows: GE, gastroesophageal; ASD, autism spectrum disorder; Abn, abnormalities; WM, white matter; VM, ventriculomegaly; CV, cerebellar vermis; CC, corpus callosum; NA, not available. Ascertainment: individuals 1 to 6, the DDD study (1 to 3 reported in previous DDD study,[6] 4 to 6 identified subsequently); individuals 7 and 8, clinical exome sequencing; individual 9, PARI 2011 study.

[a] Individual 6 has an additional probable pathogenic copy number variation (4.3 Mb duplication: dup15q15.3q21.1).

[b] Mutation reported by de Rubeis et al.[20]

[c] Mutation reported by Iossifov et al.[36]

[d] *BCL11A* mutations are annotated to transcript NM_022893.3, ENST00000335712 (GRCh37).

[e] OFC measured between percentiles 9 and 25.

[f] Severity unknown.

[g] No words at 28 months.

[h] Microstomia, with intercomissural distance ≤ -2 SD.

[i] Unilateral (left) flat helix, prominent anti-crus.

[j] Bilateral microtia (< -2 SD), asymmetric low-set ears, with overfolded and cupped helix, attached earlobes.

[k] Low-set ears with overfolded helixes and attached earlobes.

[l] Thick, overfolded helixes.

[m] Dysplastic, posteriorly rotated ears.

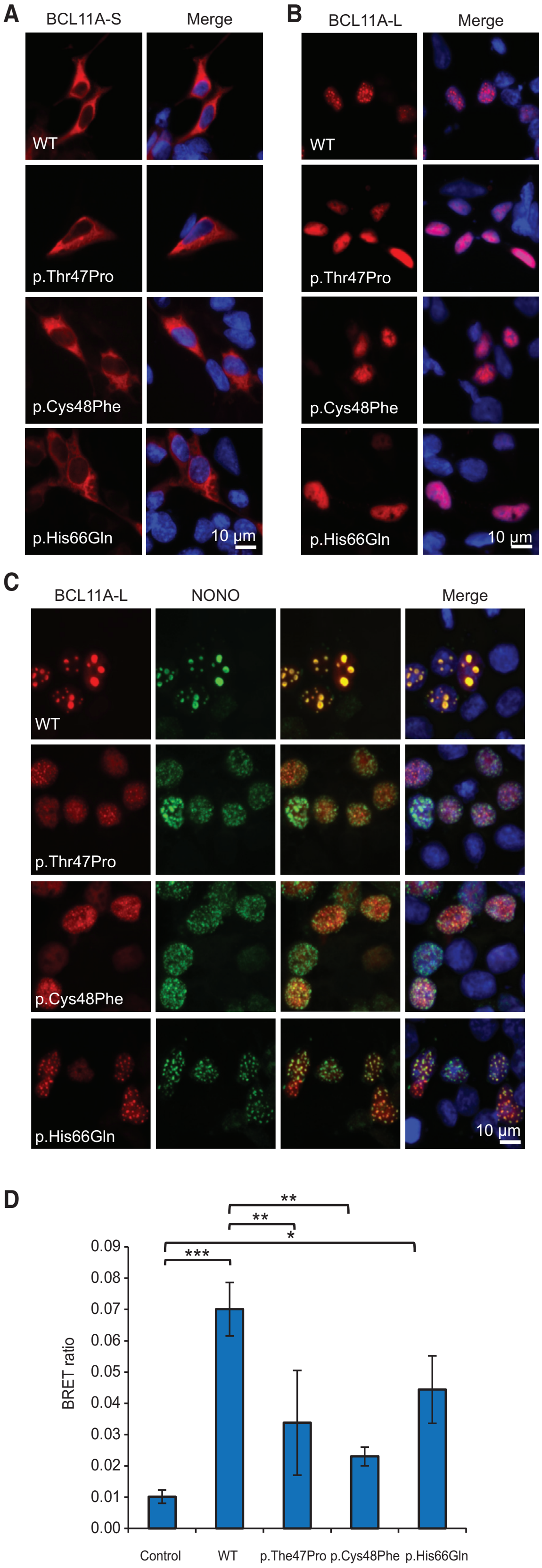

Figure 2. BCL11A Missense Substitutions Disrupt Protein Localization
(A) Fluorescence micrographs of HEK293 cells transfected with wild-type (WT) or mutant short isoform of BCL11A (BCL11A-S; GenBank: NM_138559, Ensembl: ENST00000359629) fused to mCherry (red). Nuclei were stained with Hoechst 33342 (blue).
(B) Fluorescence micrographs of HEK293 cells transfected with wild-type or mutant long isoform of BCL11A (BCL11A-L; NM_018014.3, ENST00000356842) fused to mCherry (red). Nuclei were stained with Hoechst 33342 (blue).
(C) Fluorescence micrographs of cells transfected with wild-type or mutant BCL11A-L fused to mCherry (red) and NONO fused to YFP (green). Nuclei were stained with Hoechst 33342 (blue).
Scale bars represent 10 μm.
(D) Bioluminescence resonance energy transfer (BRET) assay for interaction of wild-type or mutant BCL11A-L with NONO. HEK293 cells were transfected with BCL11A-L fused to *Renilla* luciferase, and NONO fused to YFP. Values are mean corrected BRET ratios ± SEM ($n = 3$, *$p < 0.05$, **$p < 0.01$, ***$p < 0.001$, one-way ANOVA followed by Bonferroni post hoc correction).

brain development and function, most relevant to the human disorder described here, has not been previously investigated in animal models. We first used our reporter mouse model to confirm *Bcl11a* expression in developing brain from embryonic day E10.5, with highest expression in the forebrain between E12.5 and E14.5 (Figures S1A and S1B). Central nervous system expression persists throughout the post-natal period in the cortex, hippocampus, olfactory bulb, and, to a lesser extent, in the cerebellum (Figure S1C). In the hippocampus, BCL11A localizes intensely to granule cell layers of the dentate gyrus and principal cell layers CA1, CA2, and CA3 (Figures S1D and S1E). Within the adult neocortex, localization spans layers II to VI. Although they do not show overt morphological differences, *Bcl11a*$^{+/-}$ mice appear to have a shift in *Bcl11a* localization toward deeper cortical layers relative to more superficial layers (Figure S8).

We next investigated whether murine haploinsufficiency of *Bcl11a* phenocopies the human individuals carrying *BCL11A* mutations. Microcephaly is a feature of BCL11A-associated ID (Figure 1, Table 1), particularly in individuals heterozygous for complete LoF mutations. We performed ex vivo imaging of whole mouse heads using magnetic resonance imaging (MRI) tensor-based morphometry followed by voxel-based quantification of brain volume (Figures 4A and 4B). Overall brain volume is significantly decreased in *Bcl11a*$^{+/-}$ mice, affecting both gray and white matter ($p = 1.3 \times 10^{-5}$, two-tailed F-test corrected for multiple comparisons with FDR-adjusted $p < 0.05$) (Figures 4A and 4B). Next, we normalized by overall brain volume per genotype to investigate whether specific substructures had a greater effect.[43] We found a greater decrease in the volume of regions that anatomically correspond to the limbic system—the hippocampus (corresponding to CA1, CA2, CA3, and the fimbria), corpus callosum, the posterior cortical amygdaloid nuclei, regions of the ventral and midline thalamic nuclei—and parts of the cerebellum, including regions of the superior vermis (see Allen Brain Atlas in Web Resources) (Figures 4C and S2 and Movie S4). Micro-computed tomography (μCT) revealed that skull morphology is similar in *Bcl11a*$^{+/-}$ mice and their

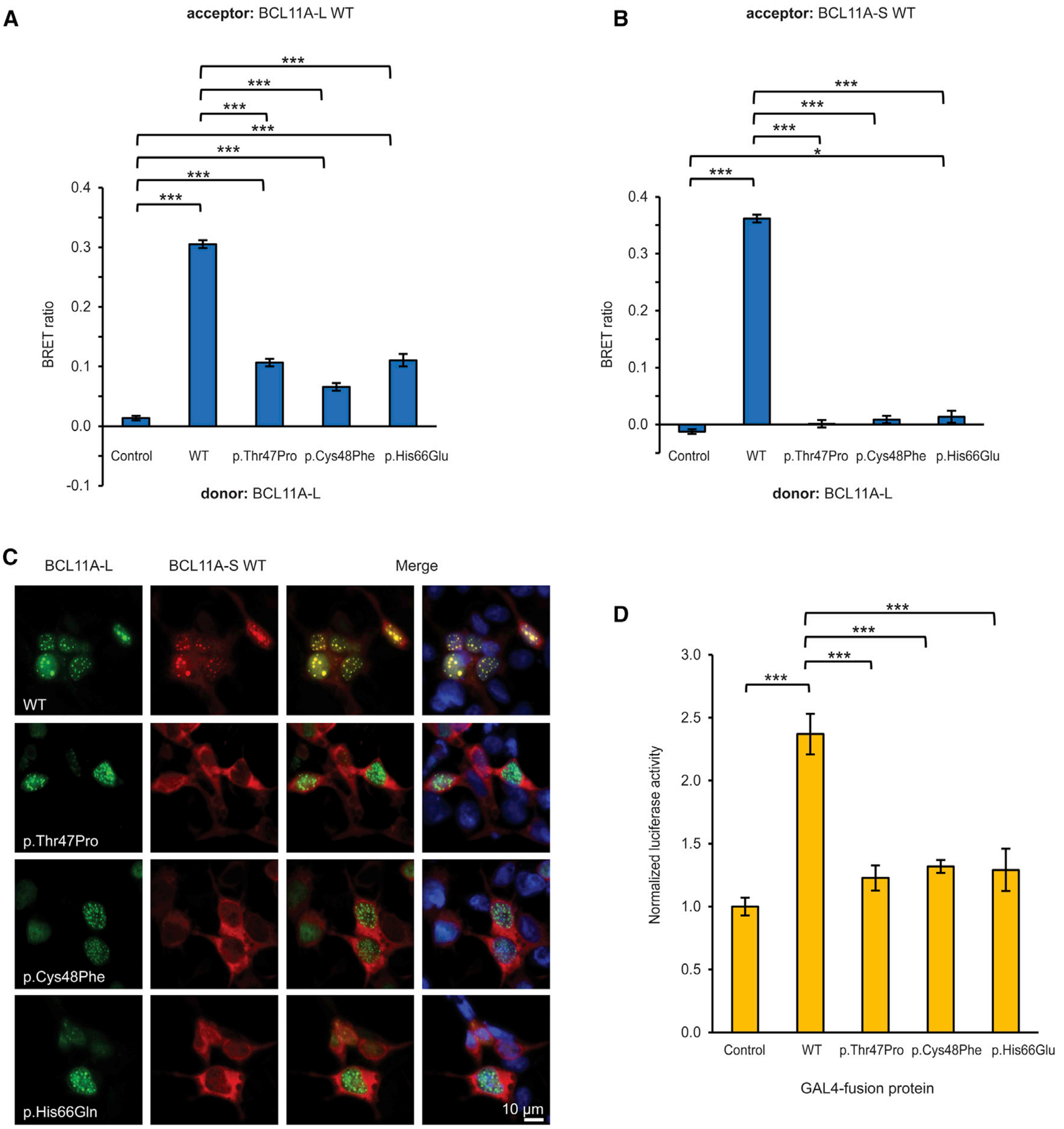

Figure 3. BCL11A Missense Substitutions Disrupt Protein Dimerization and Transcriptional Regulation

(A) BRET assay for interaction of mutant BCL11A-L (GenBank: NM_018014.3, Ensembl: ENST00000356842) with wild-type (WT) BCL11A-L. HEK293 cells were transfected with wild-type or mutant BCL11A-L fused to *Renilla* luciferase (donor) and wild-type BCL11A-L fused to YFP (acceptor). The control donor protein is a nuclear-targeted luciferase. Values are mean corrected BRET ratios ± SEM (n = 3, ***$p < 0.001$, one-way ANOVA followed by Bonferroni post hoc correction).

(B) BRET assay for interaction of mutant BCL11A-L with wild-type BCL11A-S (NM_138559, ENST00000359629). HEK293 cells were transfected with wild-type or mutant BCL11A-L fused to *Renilla* luciferase (donor) and wild-type BCL11A-S fused to YFP (acceptor). Values are mean corrected BRET ratios ± SEM (n = 3, *$p < 0.05$, ***$p < 0.001$; one-way ANOVA followed by Bonferroni post hoc correction).

(C) Fluorescence micrographs of HEK293 cells transfected with wild-type or mutant BCL11A-L fused to YFP (green), together with wild-type BCL11A-S fused to mCherry (red). Nuclei were stained with Hoechst 33342 (blue). Scale bar represents 10 μm.

(D) Mammalian one-hybrid assay for BCL11A-L transcriptional regulatory activity. HEK293 cells were transfected with wild-type or mutant BCL11A-L fused to the DNA-binding domain of GAL4, together with a firefly luciferase reporter plasmid containing GAL4 binding sites, and a *Renilla* luciferase normalization plasmid. Values are mean firefly luciferase activity normalized to *Renilla* luciferase activity ± SEM (n = 3), expressed relative to the control (***$p < 0.001$, one-way ANOVA followed by Bonferroni post hoc correction).

wild-type littermates, consistent with the absence of major congenital malformations (Figure 4D, Movies S5 and S6). However, the *Bcl11a*$^{+/-}$ skulls are significantly smaller in width (Mann Whitney $p = 0.008$ and $p = 0.032$, for bi-temporal and interzygomatic distances, respectively) but not in length ($p = 0.31$) (Figure 4E).

To investigate potential impairments in behavior, learning, and memory, we tested the *Bcl11a*$^{+/-}$ mice in

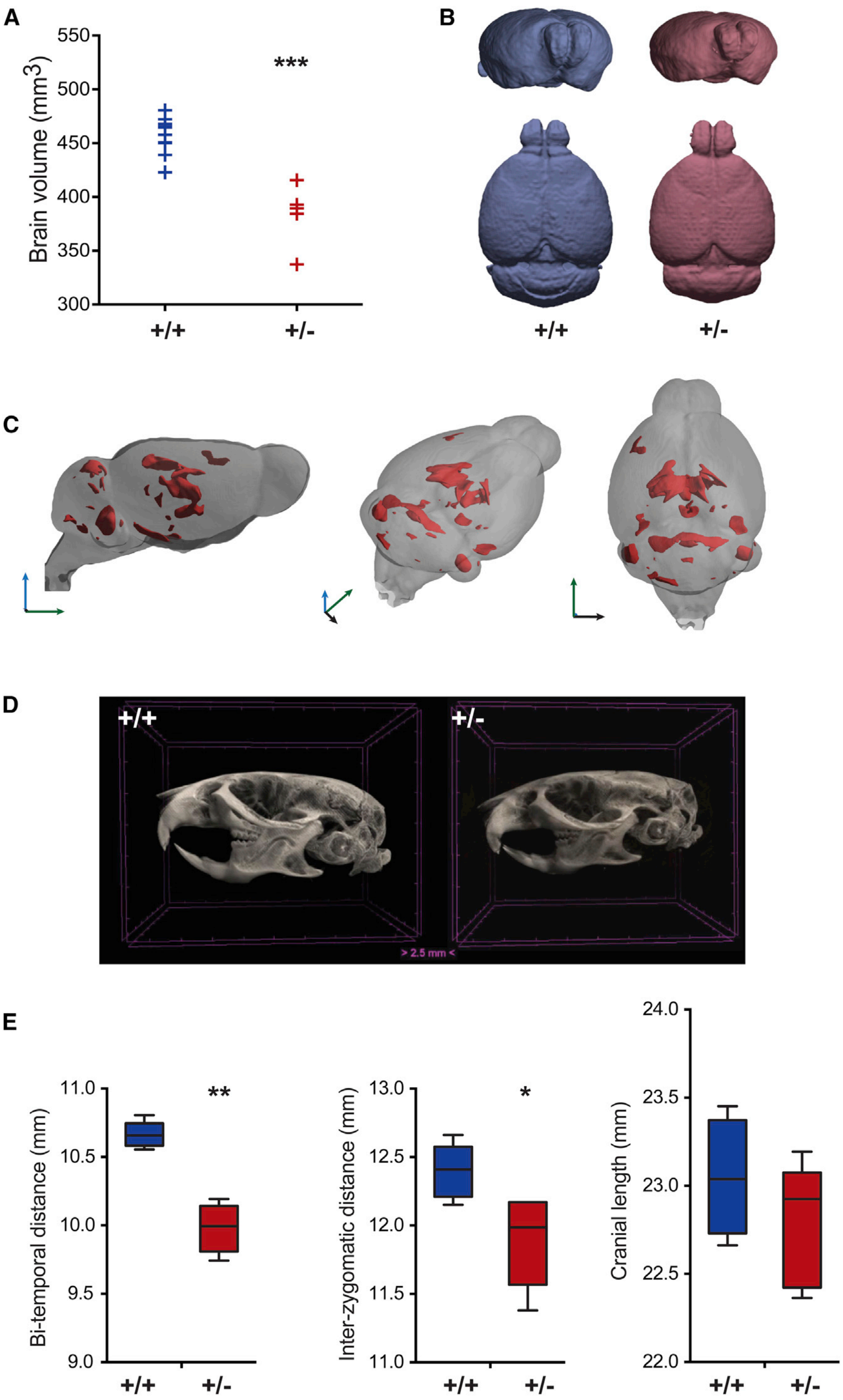

Figure 4. Neuroanatomical Imaging of Mouse Brain Reveals Microcephaly
Abbreviations are as follows: +/+, wild-type; +/−, *Bcl11a*$^{+/-}$.
(A) Total brain volume in mm^3 is significantly decreased in mutant mice (mean volumes: 457 mm^3 in wild-type; 384 mm^3 in *Bcl11a*$^{+/-}$ mice; $p = 1.3 \times 10^{-5}$ two-tailed F test corrected for multiple comparisons by controlling the false-discovery rate at $q < 0.05$).
(B) 3D reconstructions of average MRI volumes for wild-type (blue) and *Bcl11a*$^{+/-}$ (pink) mice.
(C) 3D reconstruction of heterozygous brain MRI; regions depicted in pink are significantly smaller in *Bcl11a*$^{+/-}$ mice after normalization for total brain volume. Arrows indicate orientation: blue, dorsal; green, rostral; black, ventral.
(D) 3D reconstruction of representative wild-type and *Bcl11a*$^{+/-}$ skulls demonstrating normal cranial morphology and reduced cranial size.
(E) Cranial measurements in mm are decreased in heterozygous mice (Mann-Whitney test, $^*p < 0.05$; $^{**}p < 0.01$). Boxes indicate 25th, mean, and 75th percentiles; whiskers indicate minimum and maximum values.
MRI: *Bcl11a*$^{+/+}$, n = 11; *Bcl11a*$^{+/-}$, n = 5.
μCT: *Bcl11a*$^{+/+}$, n = 5; *Bcl11a*$^{+/-}$, n = 5.

social and activity paradigms.[46] In mouse models a conspecific recognition assay can be used to test hippocampal-mediated cognitive processes,[60] using their natural propensity to investigate unfamiliar over familiar animals (Figures 5A–5C and S4A; see Subjects and Methods). On day 1 of the assay, both *Bcl11a*$^{+/-}$ mice and wild-type littermates investigated, then habituated and dishabituated to anesthetized stimulus mice (Figure 5B). In a discrimination test 24 hr later, unlike wild-type mice, the *Bcl11a*$^{+/-}$ mice were unable to differentiate between the familiar and unfamiliar stimulus mice (t test p = 0.710; mean preference index [PI] = −0.0204 ± 0.099), indicating impairment of long-term social memory (Figures 5C and S4B).

Some affected individuals with heterozygous *BCL11A* mutations have exhibited increased physical activity (repetitive behavior and hand flapping), so we tested *Bcl11a*$^{+/-}$ mice in an open field environment. The *Bcl11a*$^{+/-}$ mice displayed hyper-locomotion across the open field, with significant increase in the number of transversals and time in center (Figures S4C and S4D).

Because 30% of individuals show ASD (and overall 67% present some type of behavioral phenotype; Table 1), we sought to investigate the effects of haploinsufficiency on social behavior in our mouse model. The conspecific recognition assay showed that the mutant mice did not display an overall decreased investigation time for novel stimuli (Figures 5B and 5C), and dishabituation is consistent with intact olfaction (Figure 5B). Therefore, we performed a three-chamber social approach assay, widely used as a test for ASD-like phenotypes in rodent models.[61] Consistent with their behavior in the open field assay (Figures S4C and S4D), *Bcl11a*$^{+/-}$ mice show

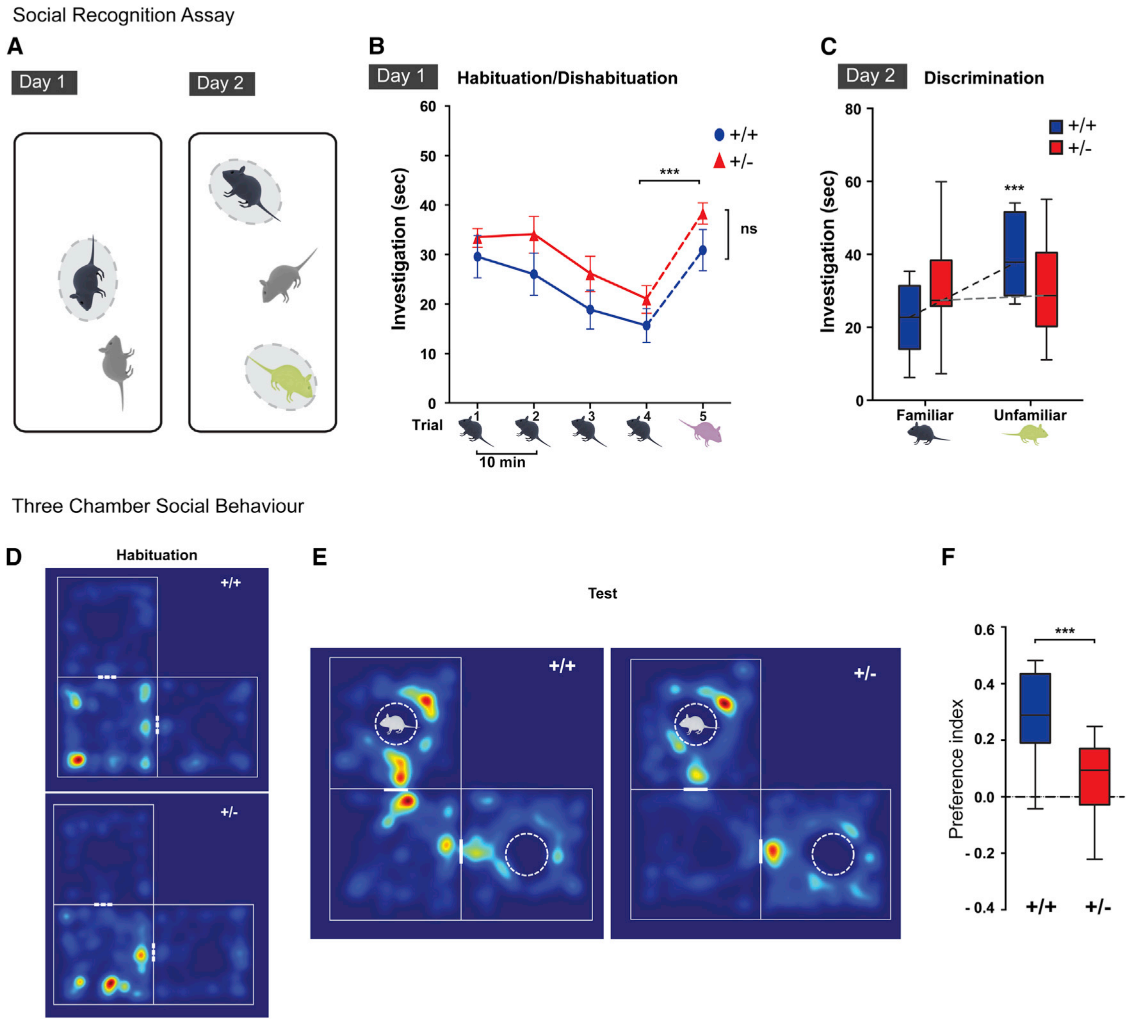

Figure 5. ***Bcl11a*$^{+/-}$ Mice Present with a Cognitive and Behavioral Phenotype**
(A) Social recognition assay: diagram of test arenas.
(B) On day 1, mice were tested for habituation-dishabituation to a conspecific (*Bcl11a*$^{+/+}$, n = 11; *Bcl11a*$^{+/-}$, n = 12). Both genotypes show decline in investigation of the same stimulus mouse (black) over four trials, with recovery on presentation of a novel stimulus mouse on trial 5 (pink): two-way ANOVA for trial 4 versus 5, ***$p < 0.0001$ for trial, with no significant interaction or difference per genotype ($p = 0.112$, ns). No significant difference between genotypes on post hoc t test (*Bcl11a*$^{+/+}$ versus *Bcl11a*$^{+/-}$, $p = 0.637$). Values are mean time in seconds (s) ± SEM.
(C) On a discrimination test 24 hr later, *Bcl11a*$^{+/-}$ animals are unable to discriminate between the familiar (presented on trials 1–4 of day 1) and novel unfamiliar (green) stimulus, unlike wild-types (2-way ANOVA, significant interaction, $p = 0.007$; post hoc t test per genotype: *Bcl11a*$^{+/+}$, ***$p = 0.0005$; *Bcl11a*$^{+/-}$, $p = 0.71$, ns).
(D and E) Three-chamber social behavior test. Heatmaps of automated tracking recordings of a representative *Bcl11a*$^{+/+}$ and a *Bcl11a*$^{+/-}$ mouse; the color gradient represents time in that location: red (maximum) to blue (minimum).
(D) On habituation to empty chambers (5 min in center + 5 min in all three), there is no preference for left or right chamber in either genotype ($p = 0.414$).
(E) Mice were then tested for preference for a novel object (cylinder) with a conspecific versus novel object only. The dashed white circles indicate the novel objects introduced: one empty cylinder, one containing a conspecific mouse represented in light gray.
(F) *Bcl11a*$^{+/-}$ mice show decreased preference for the chamber containing the object with a conspecific compared to wild-type. Boxplot of the preference index (PI) shows significantly less preference for the novel object with the conspecific in *Bcl11a*$^{+/-}$ mice. Unpaired t test, ***$p = 0.0005$; *Bcl11a*$^{+/+}$, n = 12; *Bcl11a*$^{+/-}$, n = 16.
For box and whisker plots (C and F), boxes indicate 25th, median, and 75th percentiles; whiskers indicate minimum and maximum values.

increased activity throughout the length of the test, with increased number of transversions between chambers (Figure S4G). Both genotypes have similar number of transversion in the first 2 min of the test, however, showing equal motivation to investigate novelty. Although overall time spent in the side chambers is not different between genotypes (Figure S4H), wild-type mice significantly prefer a chamber with a conspecific compared to an empty object (mean PI = 0.285 ± 0.04; paired t test per chamber for wild-types $p < 0.0001$; Figure 5). In contrast, *Bcl11a*$^{+/-}$

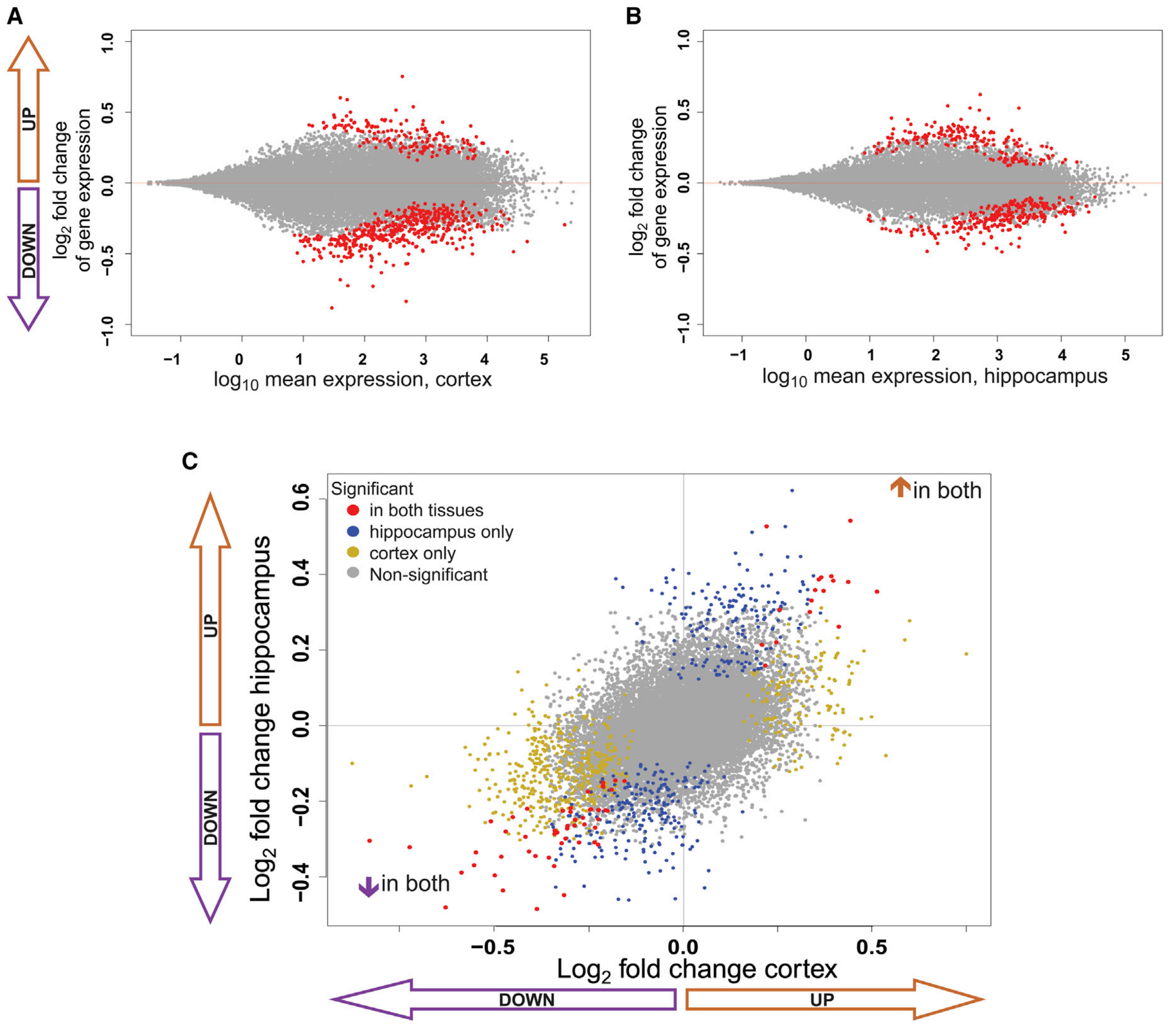

Figure 6. *Bcl11a*$^{+/-}$ Mice Have Altered Gene Expression Profiles in Cortex and Hippocampus
(A and B) MA plots of differential gene expression between *Bcl11a*$^{+/-}$ and *Bcl11a*$^{+/+}$ cortex (A) and hippocampus (B). The *x*-axis is the $\log_{10}$ average expression of all samples (normalized counts); the *y*-axis is DESeq2's shrinkage estimation of $\log_2$ fold changes between genotypes. Each gene is represented as a dot; significantly differentially expressed genes (BH-adjusted p value < 0.1) are highlighted in red. $n = 9$ per genotype for cortex; $n = 6$ per genotype for hippocampus.
(C) Comparison of DESeq2's shrinkage estimation of $\log_2$ fold changes of genes in both tissues. Red dots represent genes with significant differential expression in both tissues; yellow dots represent genes differentially expressed in the cortex only, and blue in the hippocampus only; gray dots represent genes not differentially expressed in either tissue (BH-adjusted p value < 0.1).

mice continue equal exploration throughout the test; they have a significantly decreased preference (t test for PI, mutants versus wild-types; $p = 0.0005$), spending a similar amount of time in both chambers (mean PI $= 0.017$; paired t test per chamber for mutants $p = 0.063$; Figures 5F, S4H, and S4I).

Mice with *Bcl11a* Haploinsufficiency Have Significant Transcriptional Deregulation in the Cortex and Hippocampus

Next, we used a genome-wide transcriptomic approach to obtain evidence that BCL11A is involved in transcriptional regulation in the mouse brain and to identify candidate pathways involved in disease pathophysiology. We performed RNA sequencing of cortex and hippocampus, two brain structures that are enriched for *Bcl11a* expression and are implicated in the neuroanatomical and behavioral phenotypes that we describe above. Results of the transcriptomic analysis are detailed in Table S3. For the cortex, we identified 608 differentially expressed (DE) genes in *Bcl11a*$^{+/-}$ mice compared to wild-type littermates (Benjamini-Hochberg [BH] adjusted p value with 10% FDR), of which 157 (25.8%) were upregulated and 451 (74.2%) downregulated (Figure 6A) as a consequence of haploinsufficiency. Among the DE genes, we identified significant enrichment of genes involved with syndromic ASD ($p = 0.047$) from the Gene Scoring Module of SFARI, and genes with functional evidence for ASD from the SFARI Human Gene Module ($p = 0.029$; see Methods).

For the hippocampus we identified 442 DE genes, including 183 (41.4%) upregulated and 259 (58.6%) downregulated (Figure 6B, Table S3) due to haploinsufficiency. Gene ontology analysis revealed overlapping categories in both tissues (Figure S9), including ion channel activity and transport. We analyzed specific enrichment for genes encoding voltage gated ion channels,[51] as they are associated with both ASD and ID,[2,62] and found significant enrichment in the hippocampus ($p = 0.004$) and an increase just below the significance threshold in the cortex ($p = 0.051$). These changes were predominantly downregulation (Table S3). DE genes from the BAF complex were also identified in the hippocampus: downregulation of *Smarcd1* (padj = 0.0020) of the neural progenitors-specific BAF, and of *Actl6b* (padj = 0.0095), which encodes a neuronal specific BAF complex protein.[63]

Within the gene ontology-enriched category of "cell recognition" in the cortex, we identified reciprocal upregulation of the Roundabout (Robo) receptor gene *Robo1* (padj = 0.0744) and downregulation of *Robo3* (padj = 0.002). In both tissues we also found evidence for involvement of other genes of the semaphorin-plexin pathway, putatively regulated by *Bcl11a* and involved in neuronal migration and polarity and development of neuronal circuitry.[16,64] Upregulation of semaphorins *Sema3d* (cortex, padj = 0.0037) and *Sema3e* (hippocampus, padj = 0.0047) was accompanied by reciprocal downregulation of the class3 semaphorin cell surface receptor *Plxnd1* in both tissues (padj = 0.0246, padj = 0.0038, respectively).

We performed de novo transcript assembly to test whether haploinsufficiency of *Bcl11a* alters the relative expression of its different gene isoforms. We identify transcription start sites (TSS) and sequence corresponding to the annotated isoforms: L (GenBank: NM_016707, Ensembl: ENSMUST00000000881), XL (NM_001242934, ENSMUST00000109514), XS (NM_001159290, ENSMUST00000118955), and S (NM_001159289, ENSMUST00000109516), all of which are predicted to contain all or part of the putative N-terminal dimerization region.[8,53] At the transcript level, isoforms L and XS are the most abundant transcripts in both mouse cortex and hippocampus (Figure S10 and Table S4), with a small relative increase of L in proportion to XS in *Bcl11a*$^{+/-}$ mice. The S isoform expression level is low but fully preserved in both genotypes. We provide evidence for a previously underappreciated expression of the *Bcl11a*-XS isoform usage. Importantly, the apparent preservation of expression of the S isoform in haploinsufficient cortex and hippocampus suggests a fundamental role of this isoform in the adult brain.

Finally, given that both tissues share *Bcl11a* isoform usage and have well-correlated transcriptome-wide expression patterns, we compared the patterns of differential expression between the two brain loci (Figure S11). All 69 genes that showed significant differential expression in both structures are dysregulated in the same direction. Indeed, of the 981 DE genes in either the cortex or the hippocampus, 89.3% (876) show the same direction of change in both tissues (Figures 6C and S11). This shared gene expression repertoire in the brain regions implicated in morphological, cognitive, and behavioral phenotypes supports the biological significance of these DE genes underpinning the *Bcl11a*$^{+/-}$ phenotype.

Discussion

Here we identify and phenotypically characterize an ID syndrome in individuals with missense and truncating *BCL11A* mutations. We demonstrate that the missense variants have consistent deleterious effects on BCL11A localization, dimerization, and transcriptional regulatory activity. Together with the clinical observation that affected individuals with such mutations have elevated HbF similar to loss-of-function mutations and previously identified in individuals with chromosomal microdeletions encompassing *BCL11A*,[22,23] our data support the hypothesis that these N terminus missense mutations result in a loss of BCL11A function in vivo.

The functional studies of the *BCL11A* missense mutations suggest that they are hypomorphic alleles, where the loss of function may not be complete. This could explain the similar but milder phenotypes seen in the individuals with missense mutations, as compared to what is observed in individuals with truncations, where loss of function of the affected allele is expected to be complete. These experiments also support the hypothesis that the N terminus of BCL11A is involved in protein dimerization. Notably, the DNA-binding domains of BCL11A isoforms remain intact with these mutations but functional transactivation of transcription is impaired. Thus, cell-based assays and clinical and hematological features indicate the importance of protein-protein interactions for BCL11A's role in regulating gene expression. This implies that the N terminus region of BCL11A has an underappreciated significance for transcriptional repression of fetal hemoglobin.

The clinical and cellular findings are consistent with a dosage-mediated phenotypic spectrum, with increased severity and syndromic features in individuals with truncating mutations, including blue sclerae, microcephaly, and external ear dysplasia. Although the affected individuals do not present recognizable dysmorphic features, the presence of mild dysmorphism with ID and persistence of fetal hemoglobin define a clinical syndrome, with the latter providing a valuable diagnostic tool. *BCL11A* has previously been proposed as a susceptibility gene for ASD.[20] However, we note that despite a high frequency of behavior abnormalities, only 30% of affected individuals fulfill diagnostic criteria for ASD, although all have cognitive impairment. Although it is possible that this reflects an over-representation due to ascertainment bias, it is important to note that individuals ascertained through ASD also have ID,[20] whereby ASD is a variable feature of the BCL11A-intellectual disability syndrome.

Microdeletions at 2p15–p16.1 encompassing *BCL11A* and adjacent genes/genomic regions have been associated with ID accompanied by variable additional features absent in, or of greater severity than, those in individuals with missense and LoF mutations. Genitourinary anomalies are identified in half of patients,[65–70] and camptodactyly is also a common feature.[66,68,70–73] Telecanthus, a feature of almost all individuals with microdeletions, is observed in only one individual with a LoF mutation.[19,23,65–69,71–73] CNS anomalies are detected in half of individuals, and of greater severity than those seen in the individuals presented here, including cortical dysplasia,[69] abnormalities of the corpus callosum,[65,67,68,71] white matter involvement,[68] cerebellar hypoplasia,[19,71] hypoplasia of the pons,[19] and optic nerve hypoplasia.[66,69] The smallest microdeletions include, in one case, an adjacent miRNA gene,[19] and in the other, a region centromeric to *BCL11A* with a putative transcription enhancer site.[18] In the latter report, the individual had two additional CNVs (duplications at 2q13 and 6p25.3) of unknown significance. In individuals with microdeletions, the contribution of haploinsufficiency or disruption of contiguous genes, disruption of coding and non-coding regulatory elements, or positional effects is not clear. The lack of additional malformations in individuals with missense and LoF mutations in *BCL11A* (Table 1) indicates that adjacent genes or regulatory regions contribute to those features and that *BCL11A* may be a defining gene for the developmental delay/ID phenotypes of individuals with microdeletions. Mutations in *BCL11A* therefore cause a distinct ID syndrome.

The recapitulation of affected individual phenotypes in a mouse model with heterozygous loss of function of *Bcl11a* confirms our hypothesis that haploinsufficiency of this gene alone is sufficient to cause neurodevelopmental deficits, supporting its role in brain development.

Using social- and activity-based paradigms, we investigated potential effects on behavior, learning, and memory. We demonstrated that the *Bcl11a*$^{+/-}$ mouse model displays normal novelty-seeking behavior but shows long-term social memory defects, impaired sociability, and increased physical activity. In addition to decreased overall brain size, we identified an overrepresentation of the limbic system (namely hippocampus and amygdala) among specific mouse brain regions that are more severely affected. These data together indicate that the individuals' de novo *BCL11A* mutations underlie their cognitive and behavioral phenotypes.

Given *Bcl11a*'s established role as a known transcription factor[8,11] and its association with the BAF swi/snf chromatin remodeling complex,[7] we hypothesized that the phenotype could be mediated by transcriptional dysregulation. We combined cognitive assessment and morphometry to select brain structures most severely affected by haploinsufficiency for large-scale transcriptomic analysis to investigate the genes regulated by *Bcl11a* in these structures. We identified large-scale transcriptional dysregulation in both cortex and hippocampus of *Bcl11a*$^{+/-}$ mice, with broad over-representation of genes involved in ion transport, membrane trafficking, and neuronal signaling. Interestingly, even though BCL11A is better known for its transcriptional repressor properties,[11,13] we find more genes downregulated in haploinsufficient brains, particularly in the cortex. This may be the result of downstream effects on targets that are not directly regulated by BCL11A DNA binding, or transcriptional regulation through a compromise of BAF complex function.

Several members of the BAF swi/snf complex have been implicated in mammalian neurodevelopment.[74] In the mutant hippocampus, we find downregulation of *Smarcd1* of the embryonic stem cell and neural progenitors-specific BAF complexes (esBAf and npBAF, respectively), essential for embryonic stem cell differentiation to neuroectoderm,[75] and of *Actl6b*. Recruitment of Actl6b accompanies the differentiation of progenitors into neurons.[76] Actl6b is highly specific to (but not essential for) the neuronal BAF complex (nBAF), associated with post-mitotic neuron morphogenesis and maturation.[76] Deficiency of Actl6b has also been associated with long-term, but not short-term, memory defects in mouse models.[63] Given the microcephaly present in some of the affected individuals and the reduced brain size and skull width in the mouse model, this could be suggestive of decreased neuronal proliferation or reduced survival of the post-natal neurogenic population or of post-mitotic neurons as seen postnatally in biallelic loss of *Bcl11a*.[16] Further studies are required to distinguish between these possible mechanisms in the heterozygous brain.

The differentially expressed genes are significantly enriched for those annotated as involved in ASD, transmembrane transport, and ion channel genes (Table S3).[52] There is a considerable overlap of genes involved in ID and ASD.[6,20] These transcriptional changes in a *Bcl11a*$^{+/-}$ mouse model support the hypothesis that converging molecular mechanisms underlie both disorders.

Several molecular pathways function to control axonal growth and guidance, providing cues through cell surface and secreted molecules. These include ephrins, netrins, repulsive guidance molecules, and proteins of the semaphorin-plexin and slit-robo signaling pathways (reviewed by Van Battum et al.).[77] Importantly, they are also regulators of synaptic assembly and refinement, which highlights their role in postnatal brain regulation.

Our results further implicate regulation of class 3 semaphorins by BCL11A. Class 3 semaphorins are secreted molecules that interact with plexin transmembrane receptors to regulate axon guidance, with additional roles in cell migration, vascular and lymphatic development, tumor growth and suppression, and immunologic response.[77–80] BCL11A transcriptionally represses the semaphorin Sema3c in radially migrating neurons during embryonic cortical development.[16] However, *Bcl11a*-dependent postnatal differentiation and survival does not require

SEMA3C,[16] suggesting that time-sensitive interactions of BCL11A with other targets or proteins may contribute to developmental regulation of cortical migration. We find evidence for dysregulation of semaphorin-plexin signaling in the post-natal cortex and hippocampus of *Bcl11a*$^{+/-}$ mice. Upregulation of *Sema3d* (in cortex) and *Sema3e* (in hippocampus) of our haploinsufficient mice is accompanied by reciprocal downregulation of the plexin receptor *Plxnd1*. SEMA3D regulates axon-axon interaction,[79] and SEMA3E/Plexin-D1 signaling has been implicated in forebrain axonal guidance,[81] synapse formation of cortical neurons,[82] and regulation of Cajal-Retzius cell migration in the developing cortex.[83] Class 3 semaphorins act as Plexin-D1-mediated axonal repellents or attractants, requiring and dependent on the presence of different neuropilin co-receptors.[77] Unlike SEMA3C and SEMA3D, SEMA3E appears to be able to also bind Plexin-D1 in the absence of neuropilin-1.[80]

The slit-robo signaling pathway has equally been implicated in axon guidance and angiogenesis.[84] We find opposing differential expression of *Robo3* (up) and *Robo1* (down), consistent with previous observations of suppression of ROBO1 by ROBO3 in commissural axons. ROBO3 regulates the number, migration, and differentiation of cortical interneurons,[85] whereas ROBO1 has been shown to regulate migration of cortical neurons, notably through regulation of semaphorin signaling,[86] and has been implicated in language-related disorders.[87] Thus, we identify significant effects of *Bcl11a* haploinsufficiency on expression of guidance molecules. Although further studies are required to understand the role of the class3/semaphorin and slit/robo pathways in the post-natal brain, these findings suggest that BCL11A has a broader role in post-natal synaptic regulation and neuronal and/or vascular morphogenesis of the brain than previously recognized.

Overall, our data suggest that there is an ongoing molecular phenotype in *Bcl11a*-associated ID that is not restricted to early development and involves multiple post-natal molecular pathways underlying cognitive impairment and behavioral defects. These findings raise both enthusiasm and caution with regards to therapeutic opportunities. On the one hand, the identification of dynamic post-natal transcriptional dysregulation suggests that there may be therapeutic potential to modulate the ongoing phenotype. On the other, it raises concern with regards to the inactivation of BCL11A as a target for hemoglobinopathies.[88] Given the broad transcriptional effects of reducing BCL11A dose in the brain, any therapeutic, even if restricted to erythroid lineages, would require caution.

In summary, we identify genes and pathways altered in the *BCL11A* haploinsufficient post-natal brain, suggesting non-linear dosage sensitivity of transcriptional targets or of interacting protein complexes such as the BAF complex.[7] Together, our observations underscore the importance of BCL11A dosage in mammalian brain development and as a cause of a previously undescribed BAFopathy syndrome.

Accession Numbers

The European Genome-phenome Archive accession number for the DDD Study reported in this paper is EGAS00001000775. The European Nucleotide Archive accession numbers for the RNA-seq sequences reported in this paper are ERS1159407, ERS1159409, ERS1159414, ERS1159415, ERS1159416, ERS1159417, ERS1159418, ERS1159419, ERS1159420, ERS1159421, ERS1159422, ERS1159423, ERS1159424, ERS1159425, ERS1159426, ERS1159427, ERS1159428, ERS1159429, ERS1159430, ERS1159431, ERS1159432, ERS1159434, ERS1159435, ERS1159436, ERS1159437, ERS1159438, ERS1159439, ERS1159440, ERS1159441, and ERS1159442.

Supplemental Data

Supplemental Data include 11 figures, 4 tables, and 6 movies and can be found with this article online at http://dx.doi.org/10.1016/j.ajhg.2016.05.030.

Conflicts of Interest

M.E.H. is a cofounder of, shareholder in, and consultant to Congenica Ltd., a clinical diagnostics company.

Acknowledgments

We thank the individuals and families for their participation. We thank Yvette Hooks, Edward Ryder, the Mouse Genetics Project, WTSI Research Support Facility, and sequencing pipeline staff for excellent technical support. We thank the late John Tolmie for clinical observations and unwavering support, Juexuan Wang and Yong Yu for reagents, Swathi Mookonda Chinnappa for help in generating expression constructs, Christine M. Freitag for additional clinical information, Stanislas Lyonnet for recruitment, and Jean-François Deleuze, Rolph Pfundt, and Lisenka Vissers for diagnostic services. We thank Jan F. Friedman and William Skarnes for critical review of the manuscript. The authors acknowledge the CMHD Pathology Core for technical services.

This work was supported by the Wellcome Trust (grant number WT098051), the Max Planck Society (S.E.F.), The University of Cambridge Wellcome Trust PhD Programme for Clinicians (C.D.), and EMBO Young Investigator Programme (D.W.L.).

The DDD study presents independent research commissioned by the Health Innovation Challenge Fund (grant number HICF-1009-003), a parallel funding partnership between the Wellcome Trust and the Department of Health, and the Wellcome Trust Sanger Institute. The views expressed in this publication are those of the author(s) and not necessarily those of the Wellcome Trust or the Department of Health. The study has UK Research Ethics Committee approval (10/H0305/83 granted by the Cambridge South REC and GEN/284/12 granted by the Republic of Ireland REC). The research team acknowledges the support of the National Institute for Health Research, through the Comprehensive Clinical Research Network. For a complete list of DDD Study participants, please see Firth et al.[89]

The PARI2011 study was supported by the Regional Council of Burgundy and Dijon University Hospital.

Received: March 29, 2016
Accepted: May 30, 2016
Published: July 21, 2016

Web Resources

Allen Brain Atlas, http://www.brain-map.org/
Bioconductor – DESeq2, https://bioconductor.org/packages/release/bioc/html/DESeq2.html
Bioconductor – sva, https://bioconductor.org/packages/release/bioc/html/sva.html
Cufflinks, http://cole-trapnell-lab.github.io/cufflinks/
CummeRbund, http://compbio.mit.edu/cummeRbund/
DECIPHER, http://decipher.sanger.ac.uk/
denovonear, https://github.com/jeremymcrae/denovonear
Ensembl Genome Browser, http://www.ensembl.org/index.html
European Genome-phenome Archive (EGA), https://www.ebi.ac.uk/ega
European Nucleotide Archive, http://www.ebi.ac.uk/ena
GenBank, http://www.ncbi.nlm.nih.gov/genbank/
Genetrail, http://genetrail.bioinf.uni-sb.de/
hpo similarity, https://github.com/jeremymcrae/hpo_similarity
HTSeq, http://www-huber.embl.de/users/anders/HTSeq/
IUPHAR, http://www.guidetopharmacology.org/
mupit, https://github.com/jeremymcrae/mupit
OMIM, http://www.omim.org/
SFARI Gene, https://gene.sfari.org/autdb/
STAR Aligner, https://github.com/alexdobin/STAR/releases

References

1. Kochinke, K., Zweier, C., Nijhof, B., Fenckova, M., Cizek, P., Honti, F., Keerthikumar, S., Oortveld, M.A., Kleefstra, T., Kramer, J.M., et al. (2016). Systematic phenomics analysis deconvolutes genes mutated in intellectual disability into biologically coherent modules. Am. J. Hum. Genet. *98*, 149–164.
2. Vissers, L.E.L.M., Gilissen, C., and Veltman, J.A. (2016). Genetic studies in intellectual disability and related disorders. Nat. Rev. Genet. *17*, 9–18.
3. Pinto, D., Delaby, E., Merico, D., Barbosa, M., Merikangas, A., Klei, L., Thiruvahindrapuram, B., Xu, X., Ziman, R., Wang, Z., et al. (2014). Convergence of genes and cellular pathways dysregulated in autism spectrum disorders. Am. J. Hum. Genet. *94*, 677–694.
4. McCarthy, S.E., Gillis, J., Kramer, M., Lihm, J., Yoon, S., Berstein, Y., Mistry, M., Pavlidis, P., Solomon, R., Ghiban, E., et al. (2014). De novo mutations in schizophrenia implicate chromatin remodeling and support a genetic overlap with autism and intellectual disability. Mol. Psychiatry *19*, 652–658.
5. Hoyer, J., Ekici, A.B., Endele, S., Popp, B., Zweier, C., Wiesener, A., Wohlleber, E., Dufke, A., Rossier, E., Petsch, C., et al. (2012). Haploinsufficiency of ARID1B, a member of the SWI/SNF-a chromatin-remodeling complex, is a frequent cause of intellectual disability. Am. J. Hum. Genet. *90*, 565–572.
6. Deciphering Developmental Disorders Study (2015). Large-scale discovery of novel genetic causes of developmental disorders. Nature *519*, 223–228.
7. Kadoch, C., Hargreaves, D.C., Hodges, C., Elias, L., Ho, L., Ranish, J., and Crabtree, G.R. (2013). Proteomic and bioinformatic analysis of mammalian SWI/SNF complexes identifies extensive roles in human malignancy. Nat. Genet. *45*, 592–601.
8. Avram, D., Fields, A., Senawong, T., Topark-Ngarm, A., and Leid, M. (2002). COUP-TF (chicken ovalbumin upstream promoter transcription factor)-interacting protein 1 (CTIP1) is a sequence-specific DNA binding protein. Biochem. J. *368*, 555–563.
9. Liu, P., Keller, J.R., Ortiz, M., Tessarollo, L., Rachel, R.A., Nakamura, T., Jenkins, N.A., and Copeland, N.G. (2003). Bcl11a is essential for normal lymphoid development. Nat. Immunol. *4*, 525–532.
10. Satterwhite, E., Sonoki, T., Willis, T.G., Harder, L., Nowak, R., Arriola, E.L., Liu, H., Price, H.P., Gesk, S., Steinemann, D., et al. (2001). The BCL11 gene family: involvement of BCL11A in lymphoid malignancies. Blood *98*, 3413–3420.
11. Avram, D., Fields, A., Pretty On Top, K., Nevrivy, D.J., Ishmael, J.E., and Leid, M. (2000). Isolation of a novel family of C(2)H(2) zinc finger proteins implicated in transcriptional repression mediated by chicken ovalbumin upstream promoter transcription factor (COUP-TF) orphan nuclear receptors. J. Biol. Chem. *275*, 10315–10322.
12. Khaled, W.T., Choon Lee, S., Stingl, J., Chen, X., Raza Ali, H., Rueda, O.M., Hadi, F., Wang, J., Yu, Y., Chin, S.-F., et al. (2015). BCL11A is a triple-negative breast cancer gene with critical functions in stem and progenitor cells. Nat. Commun. *6*, 5987.
13. Sankaran, V.G., Menne, T.F., Xu, J., Akie, T.E., Lettre, G., Van Handel, B., Mikkola, H.K.A., Hirschhorn, J.N., Cantor, A.B., and Orkin, S.H. (2008). Human fetal hemoglobin expression is regulated by the developmental stage-specific repressor BCL11A. Science *322*, 1839–1842.
14. Kuo, T.-Y., Hong, C.-J., and Hsueh, Y.-P. (2009). Bcl11A/CTIP1 regulates expression of DCC and MAP1b in control of axon branching and dendrite outgrowth. Mol. Cell. Neurosci. *42*, 195–207.
15. Kuo, T.-Y., and Hsueh, Y.-P. (2007). Expression of zinc finger transcription factor Bcl11A/Evi9/CTIP1 in rat brain. J. Neurosci. Res. *85*, 1628–1636.
16. Wiegreffe, C., Simon, R., Peschkes, K., Kling, C., Strehle, M., Cheng, J., Srivatsa, S., Liu, P., Jenkins, N.A., Copeland, N.G., et al. (2015). Bcl11a (Ctip1) controls migration of cortical projection neurons through regulation of Sema3c. Neuron *87*, 311–325.
17. John, A., Brylka, H., Wiegreffe, C., Simon, R., Liu, P., Jüttner, R., Crenshaw, E.B., 3rd, Luyten, F.P., Jenkins, N.A., Copeland, N.G., et al. (2012). Bcl11a is required for neuronal morphogenesis and sensory circuit formation in dorsal spinal cord development. Development *139*, 1831–1841.
18. Peter, B., Matsushita, M., Oda, K., and Raskind, W. (2014). De novo microdeletion of BCL11A is associated with severe speech sound disorder. Am. J. Med. Genet. A. *164A*, 2091–2096.
19. Balci, T.B., Sawyer, S.L., Davila, J., Humphreys, P., and Dyment, D.A. (2015). Brain malformations in a patient with deletion 2p16.1: A refinement of the phenotype to BCL11A. Eur. J. Med. Genet. *58*, 351–354.
20. De Rubeis, S., He, X., Goldberg, A.P., Poultney, C.S., Samocha, K., Cicek, A.E., Kou, Y., Liu, L., Fromer, M., Walker, S., et al.; DDD Study; Homozygosity Mapping Collaborative for Autism; UK10K Consortium (2014). Synaptic, transcriptional and chromatin genes disrupted in autism. Nature *515*, 209–215.

21. Cánovas, J., Berndt, F.A., Sepúlveda, H., Aguilar, R., Veloso, F.A., Montecino, M., Oliva, C., Maass, J.C., Sierralta, J., and Kukuljan, M. (2015). The specification of cortical subcerebral projection neurons depends on the direct repression of TBR1 by CTIP1/BCL11a. J. Neurosci. *35*, 7552–7564.
22. Funnell, A.P.W., Prontera, P., Ottaviani, V., Piccione, M., Giambona, A., Maggio, A., Ciaffoni, F., Stehling-Sun, S., Marra, M., Masiello, F., et al. (2015). 2p15-p16.1 microdeletions encompassing and proximal to BCL11A are associated with elevated HbF in addition to neurologic impairment. Blood *126*, 89–93.
23. Basak, A., Hancarova, M., Ulirsch, J.C., Balci, T.B., Trkova, M., Pelisek, M., Vlckova, M., Muzikova, K., Cermak, J., Trka, J., et al. (2015). BCL11A deletions result in fetal hemoglobin persistence and neurodevelopmental alterations. J. Clin. Invest. *125*, 2363–2368.
24. Henrichsen, C.N., Vinckenbosch, N., Zöllner, S., Chaignat, E., Pradervand, S., Schütz, F., Ruedi, M., Kaessmann, H., and Reymond, A. (2009). Segmental copy number variation shapes tissue transcriptomes. Nat. Genet. *41*, 424–429.
25. de Ligt, J., Willemsen, M.H., van Bon, B.W.M., Kleefstra, T., Yntema, H.G., Kroes, T., Vulto-van Silfhout, A.T., Koolen, D.A., de Vries, P., Gilissen, C., et al. (2012). Diagnostic exome sequencing in persons with severe intellectual disability. N. Engl. J. Med. *367*, 1921–1929.
26. McLaren, W., Pritchard, B., Rios, D., Chen, Y., Flicek, P., and Cunningham, F. (2010). Deriving the consequences of genomic variants with the Ensembl API and SNP Effect Predictor. Bioinformatics *26*, 2069–2070.
27. Iossifov, I., O'Roak, B.J., Sanders, S.J., Ronemus, M., Krumm, N., Levy, D., Stessman, H.A., Witherspoon, K.T., Vives, L., Patterson, K.E., et al. (2014). The contribution of de novo coding mutations to autism spectrum disorder. Nature *515*, 216–221.
28. Gilissen, C., Hehir-Kwa, J.Y., Thung, D.T., van de Vorst, M., van Bon, B.W.M., Willemsen, M.H., Kwint, M., Janssen, I.M., Hoischen, A., Schenck, A., et al. (2014). Genome sequencing identifies major causes of severe intellectual disability. Nature *511*, 344–347.
29. Fromer, M., Pocklington, A.J., Kavanagh, D.H., Williams, H.J., Dwyer, S., Gormley, P., Georgieva, L., Rees, E., Palta, P., Ruderfer, D.M., et al. (2014). De novo mutations in schizophrenia implicate synaptic networks. Nature *506*, 179–184.
30. Appenzeller, S., Balling, R., Barisic, N., Baulac, S., Caglayan, H., Craiu, D., De Jonghe, P., Depienne, C., Dimova, P., Djémié, T., et al.; EuroEPINOMICS-RES Consortium; Epilepsy Phenome/Genome Project; Epi4K Consortium (2014). De novo mutations in synaptic transmission genes including DNM1 cause epileptic encephalopathies. Am. J. Hum. Genet. *95*, 360–370.
31. Zaidi, S., Choi, M., Wakimoto, H., Ma, L., Jiang, J., Overton, J.D., Romano-Adesman, A., Bjornson, R.D., Breitbart, R.E., Brown, K.K., et al. (2013). De novo mutations in histone-modifying genes in congenital heart disease. Nature *498*, 220–223.
32. Allen, A.S., Berkovic, S.F., Cossette, P., Delanty, N., Dlugos, D., Eichler, E.E., Epstein, M.P., Glauser, T., Goldstein, D.B., Han, Y., et al.; Epi4K Consortium; Epilepsy Phenome/Genome Project (2013). De novo mutations in epileptic encephalopathies. Nature *501*, 217–221.
33. Sanders, S.J., Murtha, M.T., Gupta, A.R., Murdoch, J.D., Raubeson, M.J., Willsey, A.J., Ercan-Sencicek, A.G., DiLullo, N.M., Parikshak, N.N., Stein, J.L., et al. (2012). De novo mutations revealed by whole-exome sequencing are strongly associated with autism. Nature *485*, 237–241.
34. Rauch, A., Wieczorek, D., Graf, E., Wieland, T., Endele, S., Schwarzmayr, T., Albrecht, B., Bartholdi, D., Beygo, J., Di Donato, N., et al. (2012). Range of genetic mutations associated with severe non-syndromic sporadic intellectual disability: an exome sequencing study. Lancet *380*, 1674–1682.
35. O'Roak, B.J., Vives, L., Girirajan, S., Karakoc, E., Krumm, N., Coe, B.P., Levy, R., Ko, A., Lee, C., Smith, J.D., et al. (2012). Sporadic autism exomes reveal a highly interconnected protein network of de novo mutations. Nature *485*, 246–250.
36. Iossifov, I., Ronemus, M., Levy, D., Wang, Z., Hakker, I., Rosenbaum, J., Yamrom, B., Lee, Y.H., Narzisi, G., Leotta, A., et al. (2012). De novo gene disruptions in children on the autistic spectrum. Neuron *74*, 285–299.
37. Akawi, N., McRae, J., Ansari, M., Balasubramanian, M., Blyth, M., Brady, A.F., Clayton, S., Cole, T., Deshpande, C., Fitzgerald, T.W., et al.; DDD study (2015). Discovery of four recessive developmental disorders using probabilistic genotype and phenotype matching among 4,125 families. Nat. Genet. *47*, 1363–1369.
38. Samocha, K.E., Robinson, E.B., Sanders, S.J., Stevens, C., Sabo, A., McGrath, L.M., Kosmicki, J.A., Rehnström, K., Mallick, S., Kirby, A., et al. (2014). A framework for the interpretation of de novo mutation in human disease. Nat. Genet. *46*, 944–950.
39. Deriziotis, P., O'Roak, B.J., Graham, S.A., Estruch, S.B., Dimitropoulou, D., Bernier, R.A., Gerdts, J., Shendure, J., Eichler, E.E., and Fisher, S.E. (2014). De novo TBR1 mutations in sporadic autism disrupt protein functions. Nat. Commun. *5*, 4954.
40. Deriziotis, P., Graham, S.A., Estruch, S.B., and Fisher, S.E. (2014). Investigating protein-protein interactions in live cells using bioluminescence resonance energy transfer. J. Vis. Exp. *87*, e51438.
41. Lee, S.-C., and Liu, P. (2009). Construction of gene-targeting vectors by recombineering. Cold Spring Harb. Protoc. *2009*, t5291.
42. Yu, Y., Wang, J., Khaled, W., Burke, S., Li, P., Chen, X., Yang, W., Jenkins, N.A., Copeland, N.G., Zhang, S., and Liu, P. (2012). Bcl11a is essential for lymphoid development and negatively regulates p53. J. Exp. Med. *209*, 2467–2483.
43. Sawiak, S.J., Wood, N.I., Williams, G.B., Morton, A.J., and Carpenter, T.A. (2013). Voxel-based morphometry with templates and validation in a mouse model of Huntington's disease. Magn. Reson. Imaging *31*, 1522–1531.
44. Ashburner, J. (2007). A fast diffeomorphic image registration algorithm. Neuroimage *38*, 95–113.
45. deCarlos, F., Alvarez-Suárez, A., Costilla, S., Noval, I., Vega, J.A., and Cobo, J. (2011). 3D-μCT Cephalometric Measurements in Mice. In Computed Tomography - Special Applications, D.L. Saba, ed. (InTech).
46. McIntyre, R.E., Lakshminarasimhan Chavali, P., Ismail, O., Carragher, D.M., Sanchez-Andrade, G., Forment, J.V., Fu, B., Del Castillo Velasco-Herrera, M., Edwards, A., van der Weyden, L., et al.; Sanger Mouse Genetics Project (2012). Disruption of mouse Cenpj, a regulator of centriole biogenesis, phenocopies Seckel syndrome. PLoS Genet. *8*, e1003022.
47. Yang, M., Silverman, J.L., and Crawley, J.N. (2001). Automated three-chambered social approach task for mice. Curr. Protoc. Neurosci. *8*, 8.26.

48. Dobin, A., Davis, C.A., Schlesinger, F., Drenkow, J., Zaleski, C., Jha, S., Batut, P., Chaisson, M., and Gingeras, T.R. (2013). STAR: ultrafast universal RNA-seq aligner. Bioinformatics *29*, 15–21.
49. Love, M.I., Huber, W., and Anders, S. (2014). Moderated estimation of fold change and dispersion for RNA-seq data with DESeq2. Genome Biol. *15*, 550.
50. Backes, C., Keller, A., Kuentzer, J., Kneissl, B., Comtesse, N., Elnakady, Y.A., Müller, R., Meese, E., and Lenhof, H.-P. (2007). GeneTrail–advanced gene set enrichment analysis. Nucleic Acids Res. *35*, W186–W192.
51. Pawson, A.J., Sharman, J.L., Benson, H.E., Faccenda, E., Alexander, S.P.H., Buneman, O.P., Davenport, A.P., McGrath, J.C., Peters, J.A., Southan, C., et al.; NC-IUPHAR (2014). The IUPHAR/BPS Guide to PHARMACOLOGY: an expert-driven knowledgebase of drug targets and their ligands. Nucleic Acids Res. *42*, D1098–D1106.
52. Basu, S.N., Kollu, R., and Banerjee-Basu, S. (2009). AutDB: a gene reference resource for autism research. Nucleic Acids Res. *37*, D832–D836.
53. Cunningham, F., Amode, M.R., Barrell, D., Beal, K., Billis, K., Brent, S., Carvalho-Silva, D., Clapham, P., Coates, G., Fitzgerald, S., et al. (2015). Ensembl 2015. Nucleic Acids Res. *43*, D662–D669.
54. Trapnell, C., Williams, B.A., Pertea, G., Mortazavi, A., Kwan, G., van Baren, M.J., Salzberg, S.L., Wold, B.J., and Pachter, L. (2010). Transcript assembly and quantification by RNA-Seq reveals unannotated transcripts and isoform switching during cell differentiation. Nat. Biotechnol. *28*, 511–515.
55. Yuan, H., Meng, Z., Zhang, L., Luo, X., Liu, L., Chen, M., Li, X., Zhao, W., and Liang, L. (2016). A rare de novo interstitial duplication of 15q15.3q21.2 in a boy with severe short stature, hypogonadism, global developmental delay and intellectual disability. Mol. Cytogenet. *9*, 2.
56. Liu, H., Ippolito, G.C., Wall, J.K., Niu, T., Probst, L., Lee, B.-S., Pulford, K., Banham, A.H., Stockwin, L., Shaffer, A.L., et al. (2006). Functional studies of BCL11A: characterization of the conserved BCL11A-XL splice variant and its interaction with BCL6 in nuclear paraspeckles of germinal center B cells. Mol. Cancer *5*, 18.
57. Cismasiu, V.B., Adamo, K., Gecewicz, J., Duque, J., Lin, Q., and Avram, D. (2005). BCL11B functionally associates with the NuRD complex in T lymphocytes to repress targeted promoter. Oncogene *24*, 6753–6764.
58. Nakamura, T., Yamazaki, Y., Saiki, Y., Moriyama, M., Largaespada, D.A., Jenkins, N.A., and Copeland, N.G. (2000). Evi9 encodes a novel zinc finger protein that physically interacts with BCL6, a known human B-cell proto-oncogene product. Mol. Cell. Biol. *20*, 3178–3186.
59. Mircsof, D., Langouët, M., Rio, M., Moutton, S., Siquier-Pernet, K., Bole-Feysot, C., Cagnard, N., Nitschke, P., Gaspar, L., Žnidarič, M., et al.; DDD Study (2015). Mutations in NONO lead to syndromic intellectual disability and inhibitory synaptic defects. Nat. Neurosci. *18*, 1731–1736.
60. Kogan, J.H., Frankland, P.W., and Silva, A.J. (2000). Long-term memory underlying hippocampus-dependent social recognition in mice. Hippocampus *10*, 47–56.
61. Silverman, J.L., Yang, M., Lord, C., and Crawley, J.N. (2010). Behavioural phenotyping assays for mouse models of autism. Nat. Rev. Neurosci. *11*, 490–502.
62. Srivastava, A.K., and Schwartz, C.E. (2014). Intellectual disability and autism spectrum disorders: causal genes and molecular mechanisms. Neurosci. Biobehav. Rev. *46*, 161–174.
63. Vogel-Ciernia, A., Matheos, D.P., Barrett, R.M., Kramár, E.A., Azzawi, S., Chen, Y., Magnan, C.N., Zeller, M., Sylvain, A., Haettig, J., et al. (2013). The neuron-specific chromatin regulatory subunit BAF53b is necessary for synaptic plasticity and memory. Nat. Neurosci. *16*, 552–561.
64. Pasterkamp, R.J. (2012). Getting neural circuits into shape with semaphorins. Nat. Rev. Neurosci. *13*, 605–618.
65. Bagheri, H., Badduke, C., Qiao, Y., Colnaghi, R., Abramowicz, I., Alcantara, D., Dunham, C., Wen, J., Wildin, R.S., Nowaczyk, M.J.M., et al. (2016). Identifying candidate genes for 2p15p16.1 microdeletion syndrome using clinical, genomic, and functional analysis. JCI Insight *1*, e85461.
66. de Leeuw, N., Pfundt, R., Koolen, D.A., Neefs, I., Scheltinga, I., Mieloo, H., Sistermans, E.A., Nillesen, W., Smeets, D.F., de Vries, B.B.A., and Knoers, N.V. (2008). A newly recognised microdeletion syndrome involving 2p15p16.1: narrowing down the critical region by adding another patient detected by genome wide tiling path array comparative genomic hybridisation analysis. J. Med. Genet. *45*, 122–124.
67. Hucthagowder, V., Liu, T.-C., Paciorkowski, A.R., Thio, L.L., Keller, M.S., Anderson, C.D., Herman, T., Dehner, L.P., Grange, D.K., and Kulkarni, S. (2012). Chromosome 2p15p16.1 microdeletion syndrome: 2.5 Mb deletion in a patient with renal anomalies, intractable seizures and a choledochal cyst. Eur. J. Med. Genet. *55*, 485–489.
68. Piccione, M., Piro, E., Serraino, F., Cavani, S., Ciccone, R., Malacarne, M., Pierluigi, M., Vitaloni, M., Zuffardi, O., and Corsello, G. (2012). Interstitial deletion of chromosome 2p15-16.1: report of two patients and critical review of current genotype-phenotype correlation. Eur. J. Med. Genet. *55*, 238–244.
69. Rajcan-Separovic, E., Harvard, C., Liu, X., McGillivray, B., Hall, J.G., Qiao, Y., Hurlburt, J., Hildebrand, J., Mickelson, E.C.R., Holden, J.J.A., and Lewis, M.E. (2007). Clinical and molecular cytogenetic characterisation of a newly recognised microdeletion syndrome involving 2p15-16.1. J. Med. Genet. *44*, 269–276.
70. Jorgez, C.J., Rosenfeld, J.A., Wilken, N.R., Vangapandu, H.V., Sahin, A., Pham, D., Carvalho, C.M.B., Bandholz, A., Miller, A., Weaver, D.D., et al. (2014). Genitourinary defects associated with genomic deletions in 2p15 encompassing OTX1. PLoS ONE *9*, e107028.
71. Florisson, J.M.G., Mathijssen, I.M.J., Dumee, B., Hoogeboom, J.A.M., Poddighe, P.J., Oostra, B.A., Frijns, J.P., Koster, L., de Klein, A., Eussen, B., et al. (2013). Complex craniosynostosis is associated with the 2p15p16.1 microdeletion syndrome. Am. J. Med. Genet. A. *161A*, 244–253.
72. Hancarova, M., Simandlova, M., Drabova, J., Mannik, K., Kurg, A., and Sedlacek, Z. (2013). A patient with de novo 0.45 Mb deletion of 2p16.1: the role of BCL11A, PAPOLG, REL, and FLJ16341 in the 2p15-p16.1 microdeletion syndrome. Am. J. Med. Genet. A. *161A*, 865–870.
73. Félix, T.M., Petrin, A.L., Sanseverino, M.T.V., and Murray, J.C. (2010). Further characterization of microdeletion syndrome involving 2p15-p16.1. Am. J. Med. Genet. A. *152A*, 2604–2608.
74. Son, E.Y., and Crabtree, G.R. (2014). The role of BAF (mSWI/SNF) complexes in mammalian neural development. Am. J. Med. Genet. C. Semin. Med. Genet. *166C*, 333–349.
75. Alajem, A., Biran, A., Harikumar, A., Sailaja, B.S., Aaronson, Y., Livyatan, I., Nissim-Rafinia, M., Sommer, A.G., Mostoslavsky, G., Gerbasi, V.R., et al. (2015). Differential association of chromatin proteins identifies BAF60a/SMARCD1 as a regulator of embryonic stem cell differentiation. Cell Rep. *10*, 2019–2031.

76. Yoo, A.S., Staahl, B.T., Chen, L., and Crabtree, G.R. (2009). MicroRNA-mediated switching of chromatin-remodelling complexes in neural development. Nature *460*, 642–646.
77. Van Battum, E.Y., Brignani, S., and Pasterkamp, R.J. (2015). Axon guidance proteins in neurological disorders. Lancet Neurol. *14*, 532–546.
78. Sakurai, A., Doçi, C.L., and Gutkind, J.S. (2012). Semaphorin signaling in angiogenesis, lymphangiogenesis and cancer. Cell Res. *22*, 23–32.
79. Wolman, M.A., Regnery, A.M., Becker, T., Becker, C.G., and Halloran, M.C. (2007). Semaphorin3D regulates axon axon interactions by modulating levels of L1 cell adhesion molecule. J. Neurosci. *27*, 9653–9663.
80. Aghajanian, H., Choi, C., Ho, V.C., Gupta, M., Singh, M.K., and Epstein, J.A. (2014). Semaphorin 3d and semaphorin 3e direct endothelial motility through distinct molecular signaling pathways. J. Biol. Chem. *289*, 17971–17979.
81. Chauvet, S., Cohen, S., Yoshida, Y., Fekrane, L., Livet, J., Gayet, O., Segu, L., Buhot, M.-C., Jessell, T.M., Henderson, C.E., and Mann, F. (2007). Gating of Sema3E/PlexinD1 signaling by neuropilin-1 switches axonal repulsion to attraction during brain development. Neuron *56*, 807–822.
82. Wang, F., Eagleson, K.L., and Levitt, P. (2015). Positive regulation of neocortical synapse formation by the Plexin-D1 receptor. Brain Res. *1616*, 157–165.
83. Bribián, A., Nocentini, S., Llorens, F., Gil, V., Mire, E., Reginensi, D., Yoshida, Y., Mann, F., and del Río, J.A. (2014). Sema3E/PlexinD1 regulates the migration of hem-derived Cajal-Retzius cells in developing cerebral cortex. Nat. Commun. *5*, 4265.
84. Blockus, H., and Chédotal, A. (2014). The multifaceted roles of Slits and Robos in cortical circuits: from proliferation to axon guidance and neurological diseases. Curr. Opin. Neurobiol. *27*, 82–88.
85. Barber, M., Di Meglio, T., Andrews, W.D., Hernández-Miranda, L.R., Murakami, F., Chédotal, A., and Parnavelas, J.G. (2009). The role of Robo3 in the development of cortical interneurons. Cereb. Cortex *19* (*Suppl 1*), i22–i31.
86. Hernández-Miranda, L.R., Cariboni, A., Faux, C., Ruhrberg, C., Cho, J.H., Cloutier, J.-F., Eickholt, B.J., Parnavelas, J.G., and Andrews, W.D. (2011). Robo1 regulates semaphorin signaling to guide the migration of cortical interneurons through the ventral forebrain. J. Neurosci. *31*, 6174–6187.
87. Graham, S.A., and Fisher, S.E. (2015). Understanding language from a genomic perspective. Annu. Rev. Genet. *49*, 131–160.
88. Xu, J., Peng, C., Sankaran, V.G., Shao, Z., Esrick, E.B., Chong, B.G., Ippolito, G.C., Fujiwara, Y., Ebert, B.L., Tucker, P.W., and Orkin, S.H. (2011). Correction of sickle cell disease in adult mice by interference with fetal hemoglobin silencing. Science *334*, 993–996.
89. Firth, H.V., and Wright, C.F.; DDD Study (2011). The Deciphering Developmental Disorders (DDD) study. Dev. Med. Child Neurol. *53*, 702–703.

The American Journal of Human Genetics, Volume *99*

Supplemental Data

BCL11A Haploinsufficiency Causes an Intellectual Disability Syndrome and Dysregulates Transcription

Cristina Dias, Sara B. Estruch, Sarah A. Graham, Jeremy McRae, Stephen J. Sawiak, Jane A. Hurst, Shelagh K. Joss, Susan E. Holder, Jenny E.V. Morton, Claire Turner, Julien Thevenon, Kelly Mellul, Gabriela Sánchez-Andrade, Ximena Ibarra-Soria, Pelagia Deriziotis, Rui F. Santos, Song-Choon Lee, Laurence Faivre, Tjitske Kleefstra, Pentao Liu, Mathew E. Hurles, DDD Study, Simon E. Fisher, and Darren W. Logan

Supplemental Figures

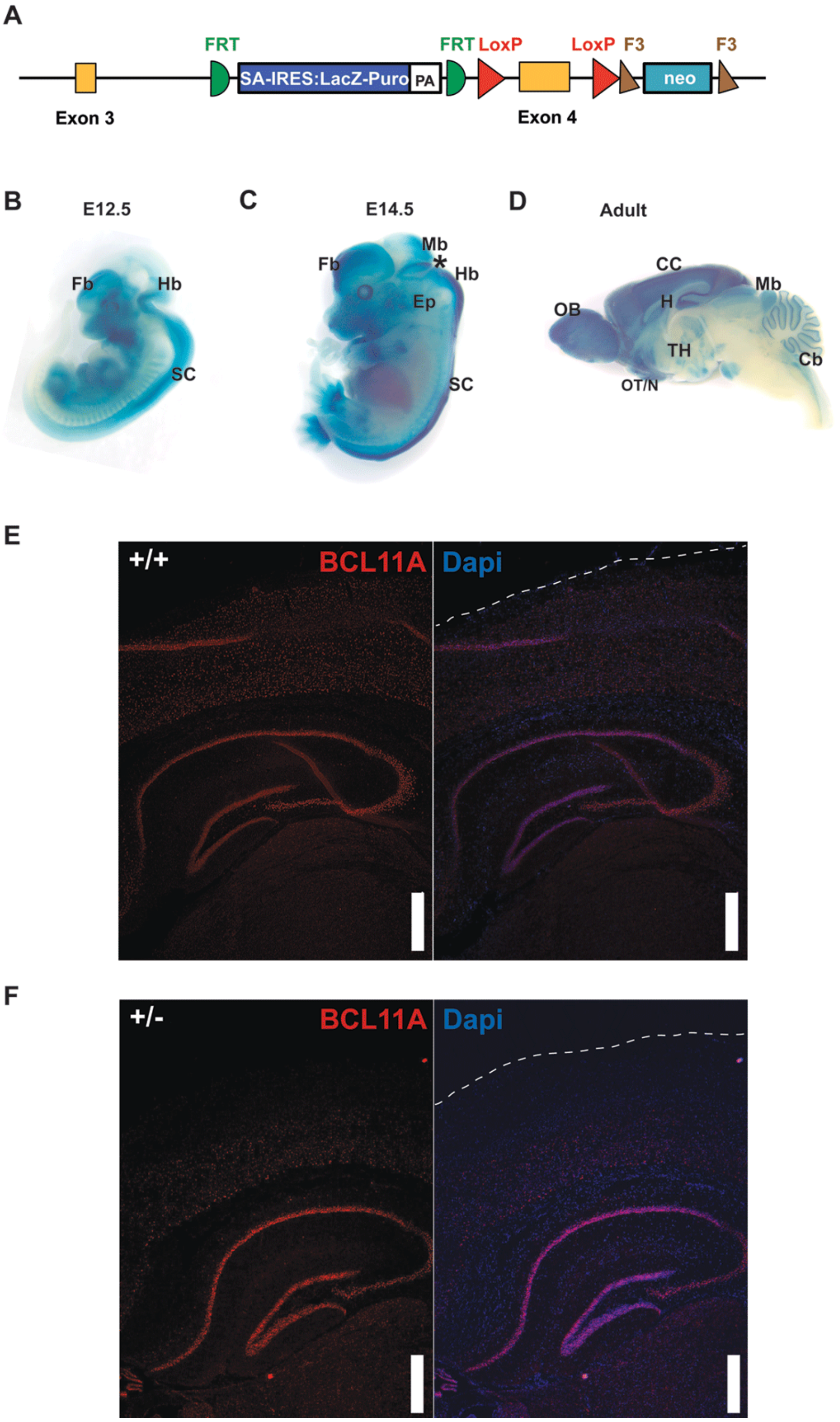

Figure S1. *Bcl11a* expression in embryo and adult brain of *LacZ* knockout first reporter mouse.

(A) Diagram of the knockout-first *LacZ* reporter allele with conditional potential. An *IRES:LacZ:Puro* promoter driven reporter cassette is inserted upstream of exon 4. **(B-D)** Whole mount images of X-gal staining patterns of *Bcl11a*$^{lacZ/+}$ mice at different developmental time

points. Expression of *Bcl11a* is first observed in the forebrain, derivatives of the first and second branchial arches and limb buds at E10.5 (not shown). **(B)** At E12.5 *Bcl11a* is high in the brain and neural tube. Specifically, it is highly expressed in the forebrain (Fb), hindbrain (Hb) and spinal cord (SC). *Bcl11a* is also seen in the somite derivatives, developing limbs, and maintains expression in the derivatives of the first two branchial arches. **(C)** At E14.5 *Bcl11a* maintains high expression in the brain, namely forebrain (Fb), midbrain (Mb) and hindbrain (Hb), with marked expression in the mid to hindbrain junction (*). Interestingly, high expression is seen in the ear pinnae (Ep). **(D)** In the adult brain, high expression of *Bcl11a* is detected in the cerebral cortex (CC), hippocampus (H), olfactory bulb (OB), olfactory tubercule and nucleus accumbens (OT/N). Parts of the thalamus (T) and a restricted area on the ventral aspect of the pons also show expression. The cerebellum (Cb), especially the purkinje cell layer, also show expression of *Bcl11a*.

E,F) Immunohistochemistry of wildtype ($Bcl11a^{+/GFP}$) and heterozygous mutant ($Bcl11a^{LacZ/GFP}$) GFP-reporter mice respectively. BCL11A localization is determined by immunolabelling using an anti-BCL11A antibody (red). Localization is identified throughout the cortical layers with exception of outer layer I, the hippocampus (specifically the pyramidal cell layer and dentate gyrus), and less intensely in the thalamus. Nuclei are counterstained with DAPI (blue). Bars: 500 µm.

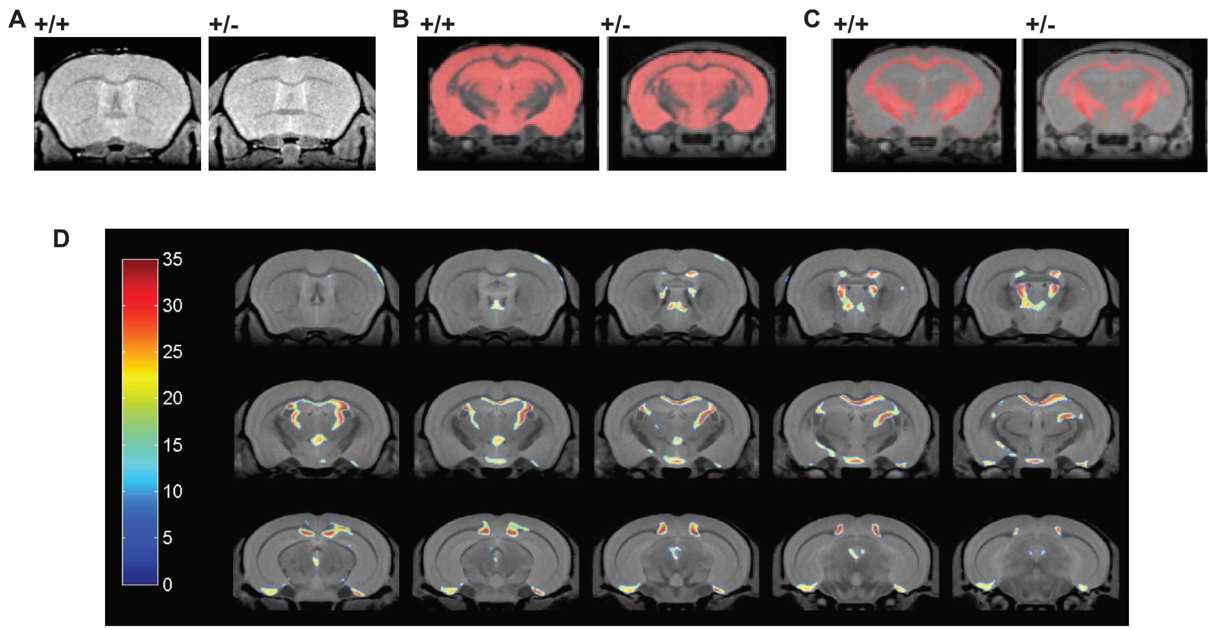

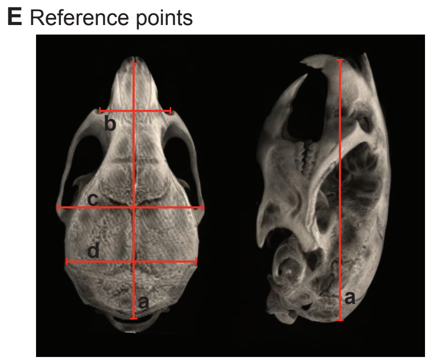

a. Cranial length: inter-nasal tip to the most distal point of the occipital bone

b. Inter-orbitary length: right to left infra-orbital foramina

c. Inter-zygomatic distance: both zygion points

d. Bi-temporal distance: measured in the more distant point of the jugal process of squamosal with respect to the sagittal plane.

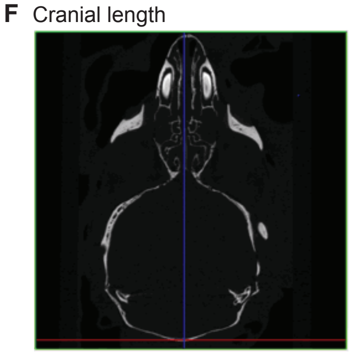

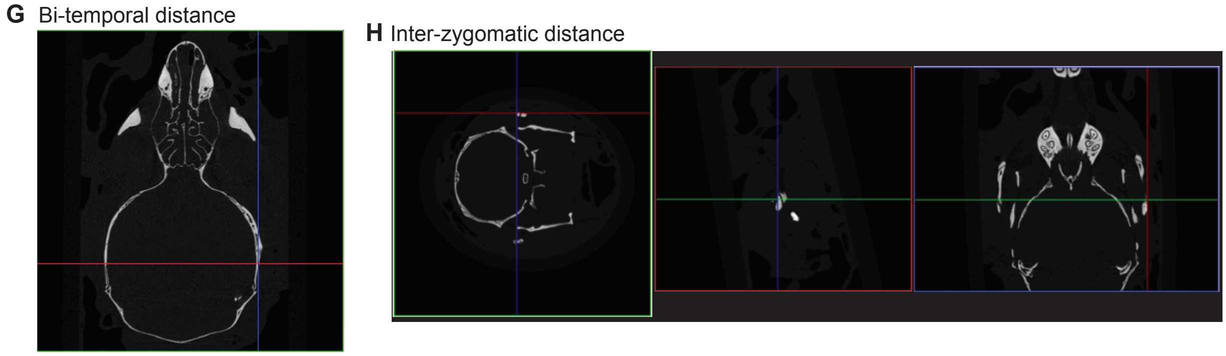

Figure S2. Neuroimaging shows microcephaly in *Bcl11a*$^{+/-}$ mice.

(A) MRI coronal sections of wildtype (**+/+**) and *Bcl11a*$^{+/-}$ **(+/-)** mice show decreased overall brain size in mutants. Representative images per genotype showing significant reduction in grey matter **(B)** and white matter **(C)** volume. **(D)** Significant changes in *Bcl11a*$^{+/-}$ mice after normalization of overall brain size are shown on representative pseudocoloured coronal

sections. Color bar indicates two-tailed F-test corrected for multiple comparisons by controlling the false-discovery rate at $q<0.05$. All changes shown are significant at $q<0.05$.

(E) to **(H)** represent 3D cranial μCT measurements methods. **E)** Landmarks used for measurements. **(F)** Cranial length, measured between the internasal (tip of the nose) and the occipital (the most distal point of the occipital bone) points. **(G)** Bi-temporal distance, measured in the most distant point of the jugal process of the squamosal bone with respect to the sagittal plane. **(H)** Inter-zygomatic distance, measured between both zygion points. As demonstrated in **(H)**, all measurements were corrected using a multiplanar visualization of reference points, in sagittal, axial and coronal planes.

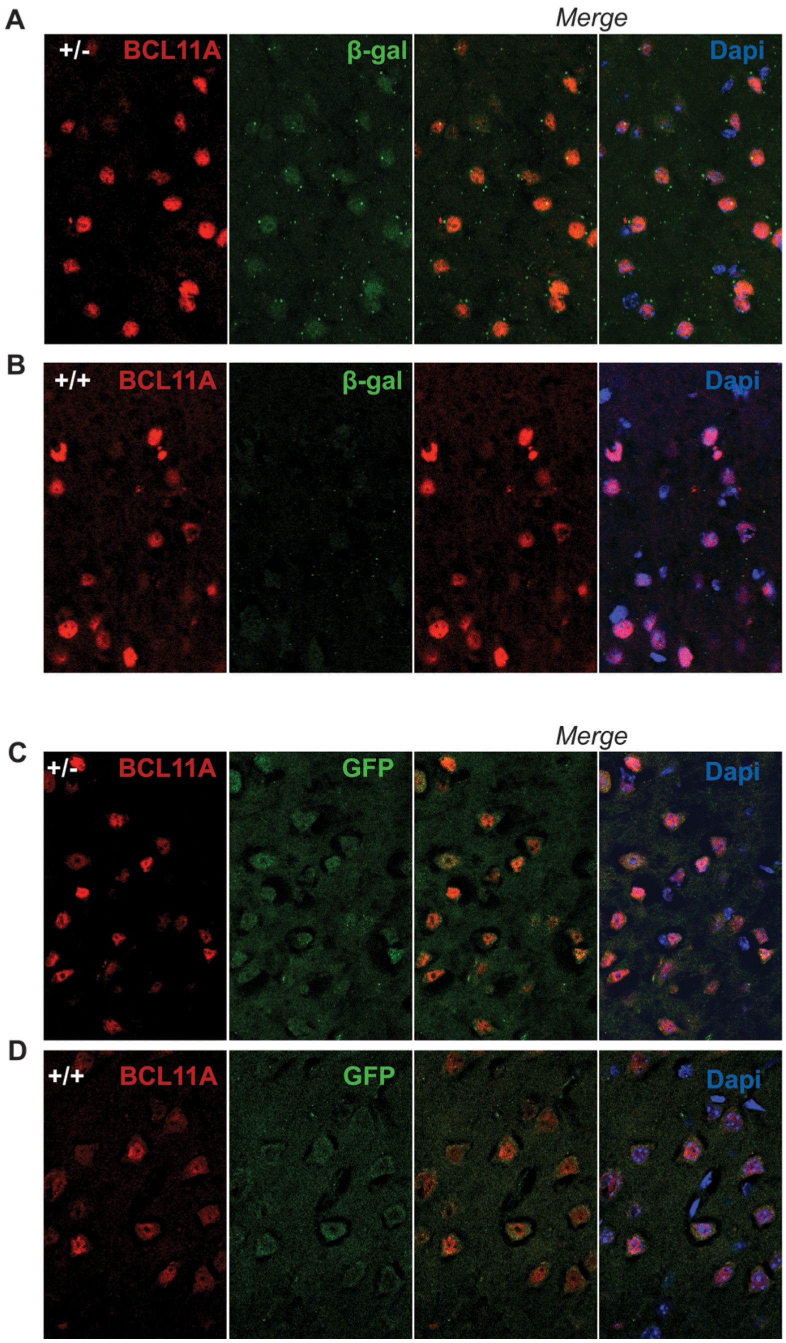

Figure S3. BCL11A antibody immunostaining.

Immunostaining of cortical cryosections in mutant (*Bcl11a*$^{LacZ/GFP}$, represented by +/-) and wildtype (*Bcl11a*$^{+/GFP}$, represented by +/+) GFP reporter mice (with a primary antibody predicted to detect the L and XL isoforms) shows nuclear staining. **(A)** An anti-BCL11A Ab (red) co-localizes with anti-β-GAL (β-GAL, green) in the mutant (heterozygote for the knockout-first *LacZ* reporter allele), which is absent in the wildtype **(B)**. Anti-GFP Ab (green) was employed to detect the functional GFP reporter allele **(C,D)**, which co-localizes with anti-BCL11A (red) in nuclei. Nuclei are counterstained with DAPI (blue).

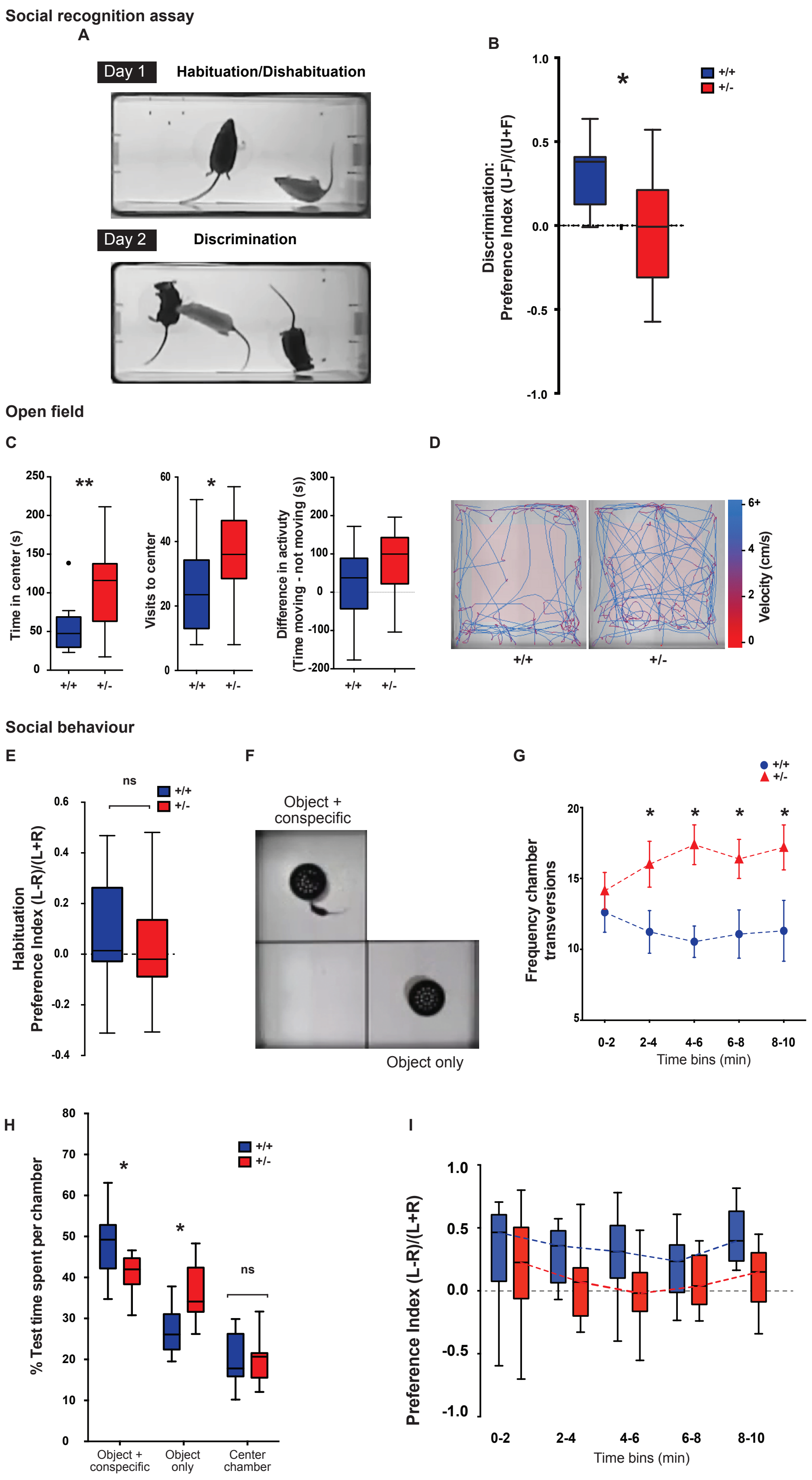

Figure S4. Cognitive and behavioral phenotyping of *Bcl11a*$^{+/-}$ mice.

(A) Overhead image of a representative test arena for days 1 (top), and 2 (bottom) social recognition assay. In the example presented, the test *Bcl11a*$^{+/-}$ mice are a light coat color (albino), and the stimuli are dark. On both days prior to testing mice are habituated for ≥1 hour in the behavioral test room and ≥ 10 minutes to the empty test cage; habituation and test are performed under red light. On day 1, test animals are presented with an anaesthetized conspecific for 1 minute, at 10-minute intervals, for 4 trials. On the 5th trial, a novel stimulus is presented. Investigation time, identified as close proximity sniffing, oronasal contact, or approaching within 1-2cm, is recorded over 2 minutes. **(B)** Social discrimination preference index (PI) values greater than 0.5 (dotted line) indicate that the test mice spent greater amount of time investigating the unfamiliar stimulus compared with the familiar stimulus. *Bcl11a*$^{+/+}$, *n*=11; *Bcl11a*$^{+/-}$, *n*=12. *Bcl11a*$^{+/-}$ mice have a significantly decreased preference for the unfamiliar stimulus compared to the wildtype (t-test, **p*=0.012). Boxes indicate 25th, median and 75th percentiles; whiskers indicate minimum and maximum values.

(C,D) Open field activity. *Bcl11a*$^{+/-}$ mice spent more time in the center (Mann-Whitney, ***p*=0.003) than wildtype littermates. The number of visits to the center was also increased (t-test, **p*=0.025). Increased difference in activity (defined as the difference between the time moving and not moving) was just below statistical significance (t-test, *p*=0.091), as was velocity (t-test, *p*=0.0892). Tukey boxplots; black dots indicate outliers (*Bcl11a*$^{+/+}$, *n*=14; *Bcl11a*$^{+/-}$, *n*=17). **(D)** Examples of trajectories (representative tracks closest to the mean) of a wildtype (+/+) and *Bcl11a*$^{+/-}$ mouse.

(E-G) Three chamber social behavior test. Mice are habituated for ≥1 hour in the behavioral test room; habituation and test are performed under red light. Test mice are habituated to the center chamber for 5 minutes, and to the three chambers (by opening doors to left and right chambers) for another 5 minutes. During habituation, movements are tracked with an automated recording system. The preference index for left vs. right chamber is calculated by the following equation: [PI=(time in left chamber – time in right chamber)/ (time in left chamber + time in right chamber)]. No significant difference between genotype was detected (t-test, *p*=0.414). Boxes indicate 25th, median and 75th percentiles; whiskers indicate minimum and maximum values. **(F)** A cylindrical metal container with holes that fit a mouse nose tip is placed in the two side chambers: one containing a live conspecific stimulus, the other empty. An automated recording system is used to track the movements of the test mouse and record time spent in each chamber during a 10 minute test. **(G)** Number of transversions from one chamber to another over the test time in 2 minute time bins is significantly increased after the first 2 minutes in *Bcl11a*$^{+/-}$ mice (t-test per time bin, *p*<0.05 for minutes 2 to 10; 2-way ANOVA *p*=0.0068 for genotype). Values are mean number of transversions ± S.E.M.

(H) The boxplot represents the percentage of time spent in each chamber (boxes indicate 25th, median and 75th percentiles; whiskers indicate minimum and maximum values). There is a significant difference between genotypes for time spent in the object + conspecific chamber (**p*=0.003, t-test) and for time spent in object only chamber (**p*=0.0007), but not for the empty center chamber (*p*=0.826). **(I)** Boxplots of preference index across 2 minute time bins (boxes indicate 25th, median and 75th percentiles; whiskers indicate minimum and maximum values; *Bcl11a*$^{+/+}$, *n*=12; *Bcl11a*$^{+/-}$, *n*=16. Black dots indicate outliers).

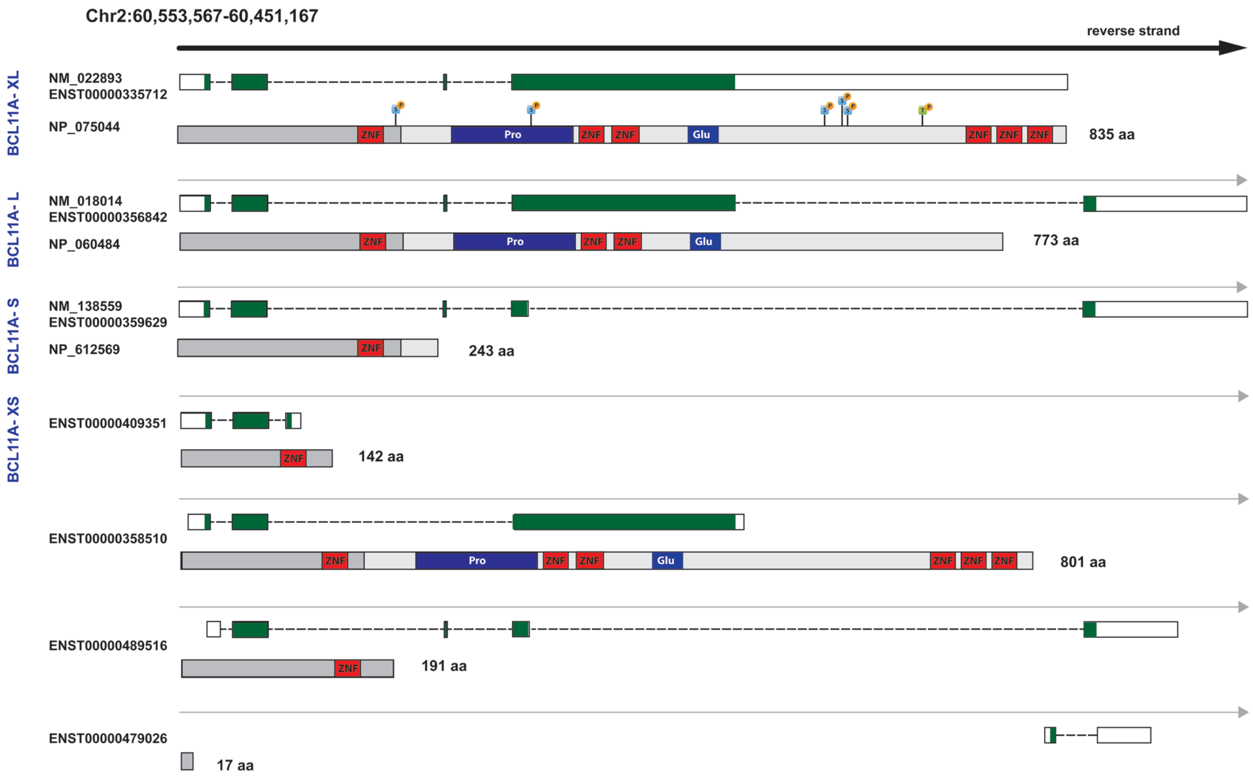

Figure S5. BCL11A isoforms. Isoforms putatively encoded by *BCL11A* and annotated in RefSeq and Ensembl. Accession numbers are indicated in black, and "common" name for the 4 isoforms reported are in blue. A schematic diagram of alternative exon usage is provided above each predicted protein. Top: Exons are shaded dark green; UTRs are white. Bottom: C2H2 zinc finger domains are represented in red. Putative region required for SUMO1 recruitment is shaded grey. Proline (Pro) and Glutamate (Glu) rich region are shaded blue. aa, aminoacids. On the XL isoform, post-translational modifications annotated in UniProt are indicated: phosphorylated serine in blue, phosphorylated threonine in green.

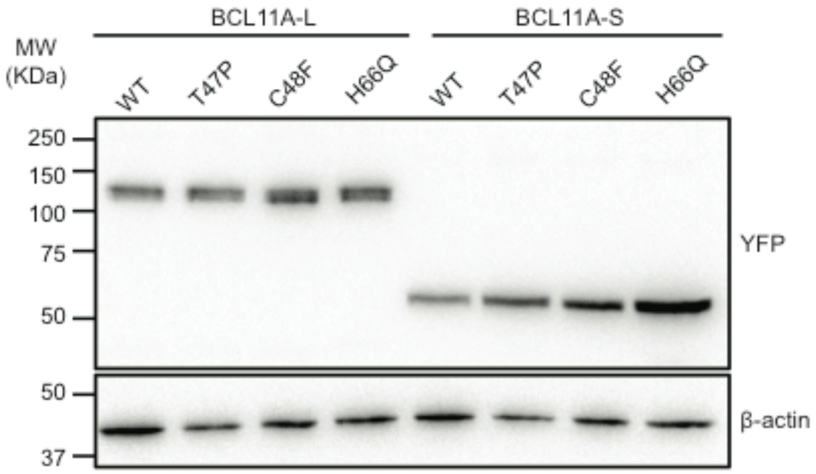

Figure S6. Western blot of mutant and wildtype BCL11A-L and BCL11A-S. HEK293 cells were transfected with mutant or wildtype (WT) *BCL11A* fused to YFP. Blots of whole cell lysates were probed with anti-YFP to detect BCL11A and with anti-β-ACTIN to confirm equal loading. BCL11A-L: NM_018014.3, ENST00000356842; BCL11A-S: NM_138559, ENST00000359629.

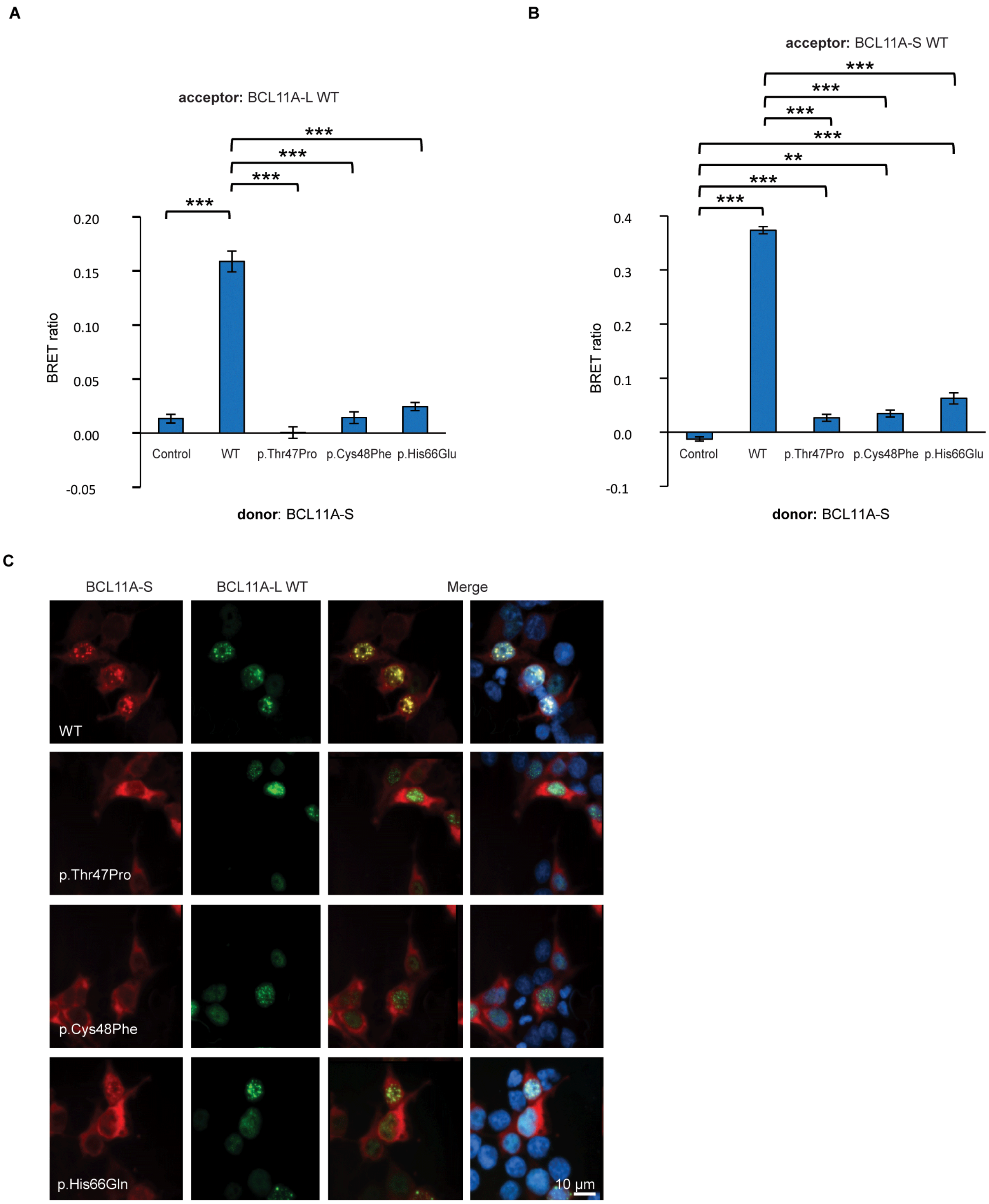

Figure S7. *BCL11A* missense mutations identified in affected individuals disrupt protein dimerization. (A) BRET assay for interaction of mutant BCL11A-S with wildtype (WT) BCL11A-L. HEK293 cells were transfected with wildtype or mutant BCL11A-S fused to *Renilla* luciferase (donor) and wildtype BCL11A-L fused to YFP (acceptor). Values are mean corrected BRET ratios ± S.E.M. (n= 3, *p<0.05, **p<0.01, ***p<0.001, *one-way ANOVA* followed by *Bonferroni* post-hoc correction). **(B)** BRET assay for interaction of mutant BCL11A-S with wildtype BCL11A-S. HEK293 cells were transfected with wildtype or mutant BCL11A-S fused to *Renilla*

luciferase (donor) and wildtype BCL11A-S fused to YFP (acceptor). Values are mean corrected BRET ratios ± S.E.M. (n= 3, *p<0.05, **p<0.01, ***p<0.001, *one-way ANOVA* followed by *Bonferroni* post-hoc correction). **(C)** Fluorescence micrographs of HEK293 cells transfected with wildtype or mutant BCL11A-S fused to mCherry and wildtype BCL11A-L fused to YFP. Nuclei were stained with Hoechst 33342 (blue). BCL11A-S: NP_612569.1; BCL11A-L: NP_060484.2.

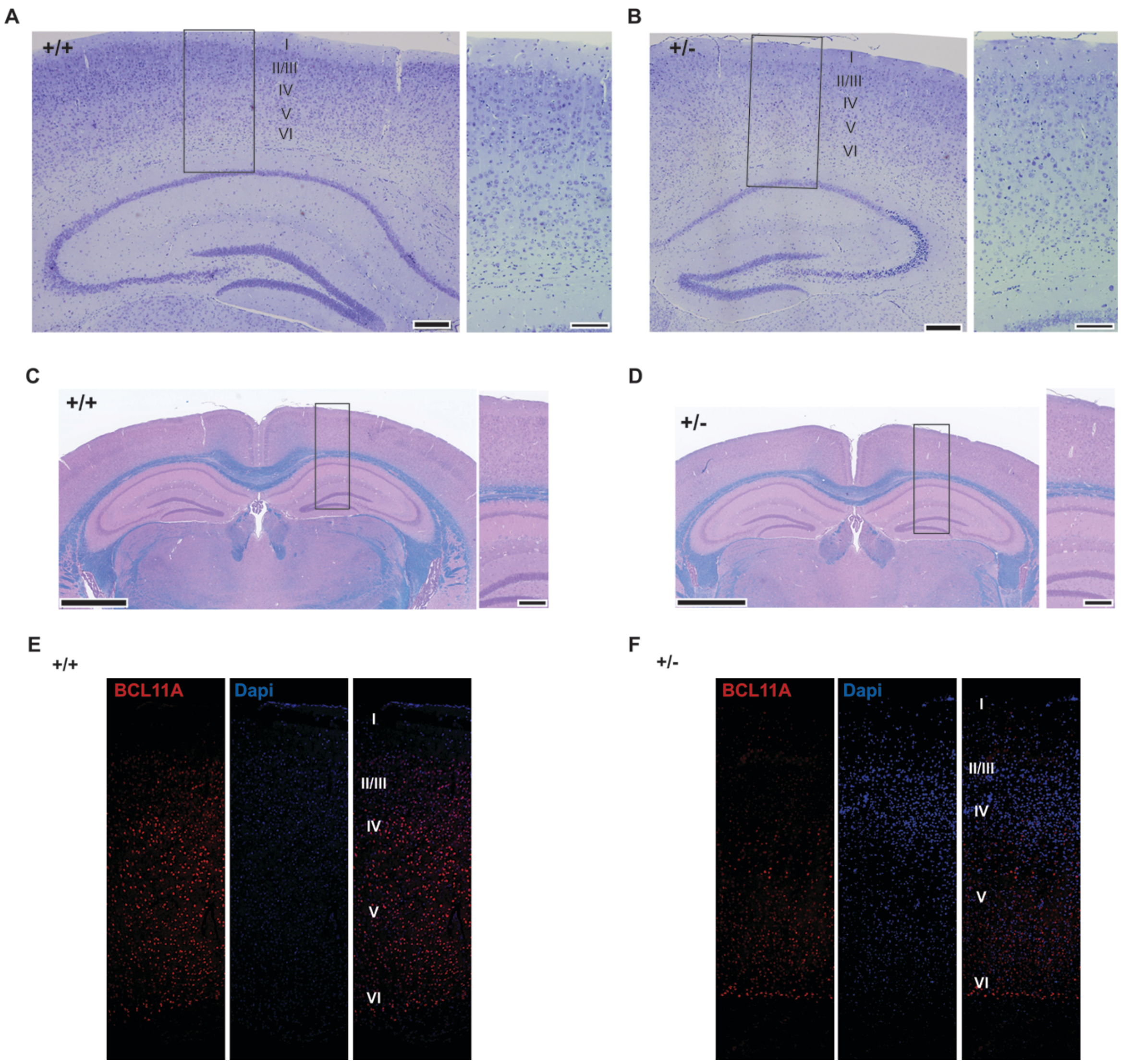

Figure S8. Cortical morphology in wildtype and *Bcl11a*$^{+/-}$ mice. *Bcl11a* haploinsufficient mice (+/-, **B,D**) do not present gross morphological defects in comparison to wildtype littermates (+/+, **A,C**). Nissl and luxol fast blue staining of paraffin embedded sections of brains of *Bcl11a*$^{+/-}$ mice **(B,D)** displayed brain architecture similar to wildtype littermates **(A,C)** at 16 weeks, specifically with normal structure of the neocortex and hippocampus. Higher magnifications are delimited by inset boxes. Bars: A,C: 200 µm (inset amplification, 100 µm); B,D: 1mm (inset amplification, 200 µm).

E,F: Immunostaining of the cortex for BCL11A (first panels, red) in mutants (+/-, **F**) mice and wildtype littermates (+/+, **E**) and DAPI nuclear counter-stain (second panels, blue); the third panels are the merged images. BCL11A localization is detected in layers II to VI in both genotypes. Unlike wildtype, *Bcl11a*$^{+/-}$ mice show more BCL11A stained nuclei in deep layers in comparison to upper layers.

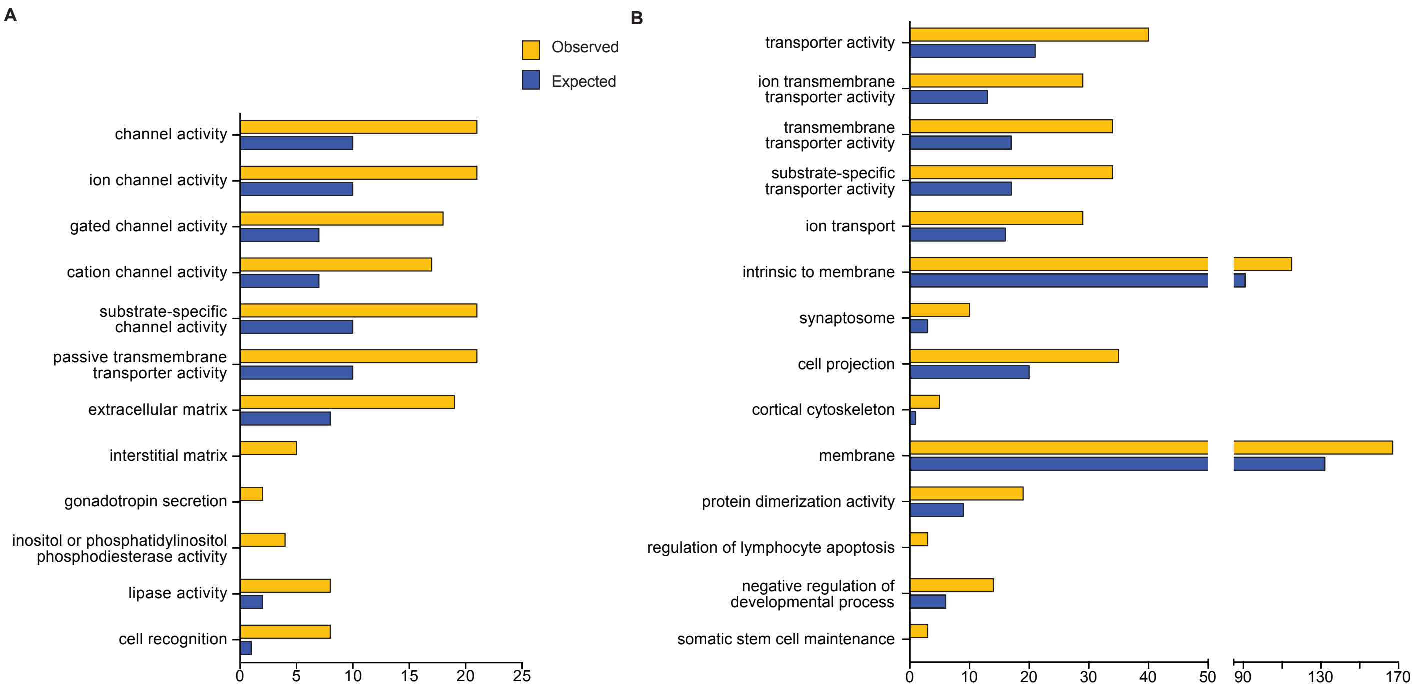

Figure S9. Gene ontology enrichment analysis of differentially expressed genes in *Bcl11a*$^{+/-}$ mice.

Selected categories of the GeneTrail over-representation analysis of differentially expressed genes in the cortex **(A)** and hippocampus **(B)** compared to a reference set of genes expressed in each wildtype tissue. The blue bar indicates the expected number of genes; the yellow bars indicate the observed number of genes for gene ontology terms. Significance threshold $p<0.1$ Hypergeometric test with FDR adjustment (Benjamini Hochberg, 1995) for multiple testing (complete analysis results and individual *p-value*s available in Supplemental Table 2).

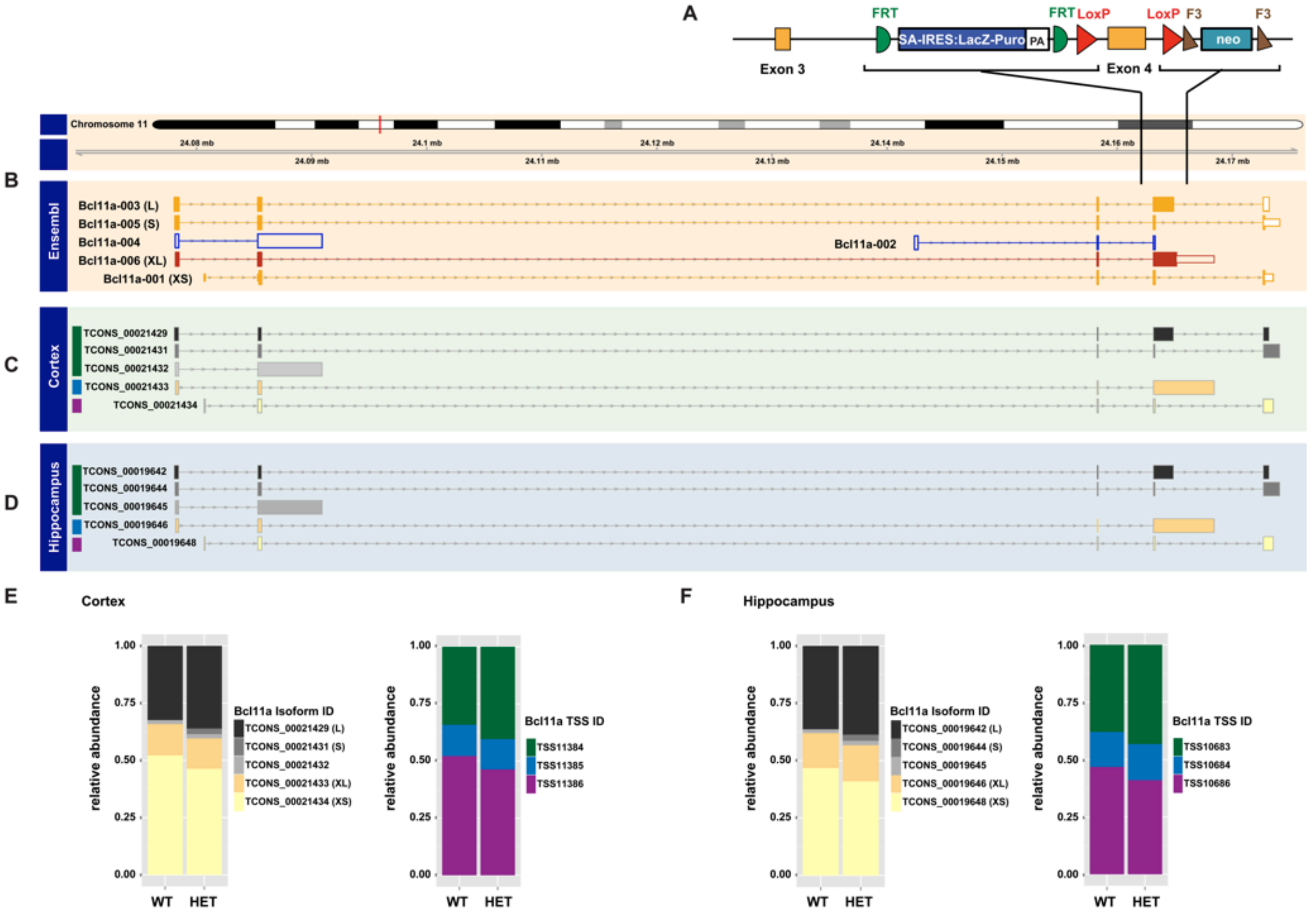

Figure S10. Bcl11a *LacZ* reporter allele and isoform expression.

(A) Diagram of the knockout-first *LacZ* reporter allele with conditional potential described in Figure S3. The cassette disrupts transcription of all reported protein-coding isoforms as represented in **(B)** (chromosome 2 ideogram and Ensembl release 82 isoforms shown; graphical representation of isoforms created with CummeRbund v2.12.0). Isoforms represented in yellow and red are protein coding; blue isoforms do not produce protein. Bcl11a-001: isoform XS, NM_001159290, ENSMUST00000118955; Bcl11a-002: processed transcript ENSMUST00000127494; Bcl11a-003: isoform L, NM_016707, ENSMUST00000000881; Bcl11a-004: retained intron ENSMUST00000124148; Bcl11a-005: isoform S, NM_001159289, ENSMUST00000109516; Bcl11a-006: isoform XL, NM_001242934, ENSMUST00000109514. Isoforms assembled in Cufflinks v.2.2.1 with fpkm≥0.1 in cortex and hippocampus (both genotypes pooled per tissue) are represented in **(C)** and **(D)** respectively. Differential expression (without correction for batch effect) of each isoform per genotype and respective transcription start site usage are represented in **(E)** and **(F)**. The four major mouse isoforms (L, S, XL and XS) are expressed in both tissues in similar proportions. There is a relative decrease of isoform XS in favor of L and S in mutants (HET) when compared to wildtype (WT).

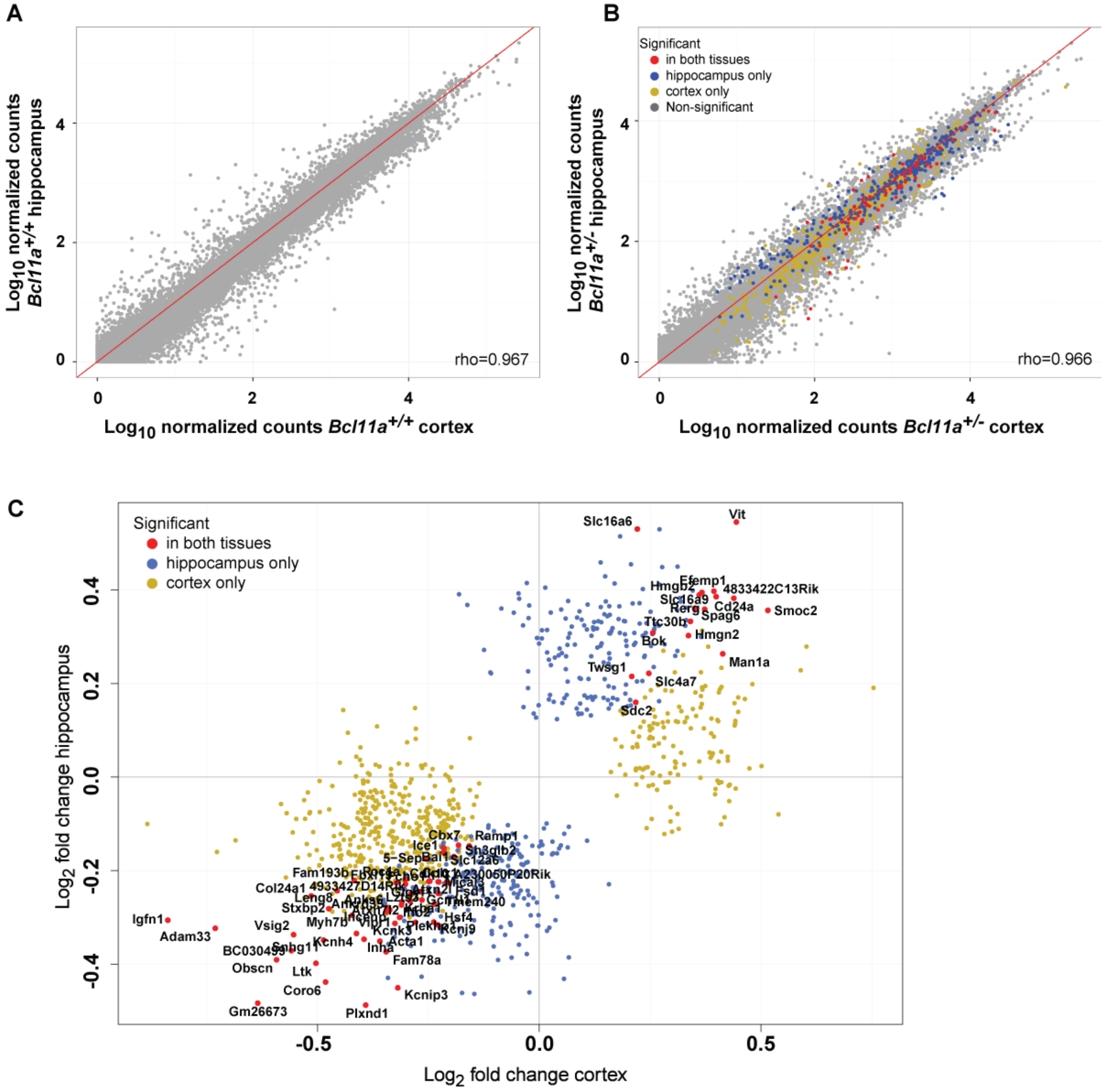

Figure S11. Comparison of gene expression per tissue.

A) Distribution of $\log_{10}$+1 normalized counts in wildtype cortex vs. hippocampus. Spearman's correlation value in bottom right corner ($p<2.2e^{-16}$). **B)** Distribution of $\log_{10}$+1 normalized counts in *Bcl11a*$^{+/-}$ cortex vs. hippocampus. Spearman's correlation value in bottom right corner ($p<2.2e^{-16}$). Differentially expressed genes are represented in color. **C)** Comparison of DESeq2's shrinkage estimation of log fold changes of genes in both tissues as in Figure 6. Red dots represent genes with significant differential expression in both tissues, labeled by gene name. Yellow dots represent genes differentially expressed in the cortex only, and blue in the hippocampus only (BH-adjusted *p*-value <0.1). Non-differentially expressed genes are not represented.

Gene	Forward primer	Site	Reverse primer	Site
BCL11A-S	GGATCCTGTCTCGCCGCAAGCAAGGC	BamHI	GCTAGCTCAAATTTTCTCAGAACTTAAGGGC	NheI
BCL11A-L	GGATCCTGTCTCGCCGCAAGCAAGGC	BamHI	GCTAGCTCAGAACTTAAGGGCTCTCG	NheI
NONO	GGATCCAGAGTAATAAAACTTTTAACTTGG	BamHI	TCTAGATTAGTATCGGCGACGTTTGTTTGGG	XbaI

Table S1. Primers used for human fetal brain cDNA amplification. Restriction sites are underlined. NheI and XbaI have compatible overhangs.

Variant	Primer 1	Primer 2
BCL11A T47P	GCACTGCCCACAGGGGAGGAGGTCATGAT	ATCATGACCTCCTCCCCTGTGGGCAGTGC
BCL11A C48F	GGCACTGCCCAAAGGTGAGGAGGTCATGATCC	GGATCATGACCTCCTCACCTTTGGGCAGTGCC
BCL11A H66Q	CTGCCATTGCATTGTTTCCGTTTTTGCTCGATAAAAATAAGAATGTC	GACATTCTTATTTTTATCGAGCAAAAACGGAAACAATGCAATGGCAG

Table S2. Primers used for introduction of missense mutations identified in affected individuals in DNA constructs.

Table S3. RNA-Seq of *Bcl11a*$^{+/-}$ *and Bcl11a*$^{+/+}$ mouse cortex and hippocampus.

Provided as a separate .xlsx file

Excel file with sheets containing RNASeq analysis of cortex and hippocampus: RNASeq normalized read counts, results tables from DESeq2 differential expression analysis, gene ontology enrichment analysis and *Bcl11a* isoform predictions determined with Cufflinks.

Table S4. Gene lists used in specific gene enrichment analysis.

Provided as a separate xlsx file

Excel file with sheets containing gene subset for each enrichment analysis.
